# Introgressed Variation in *TaMYB7‐A1* Drives Graded Dormancy and Climate‐Adaptive Pre‐Harvest Sprouting Resistance in Wheat

**DOI:** 10.1002/advs.202524067

**Published:** 2026-05-07

**Authors:** Hao Wang, Dongzhi Wang, Yafei Guo, Qi Zheng, Min Zhang, James Simmonds, Jian Hou, Xuemei Liu, Xuelei Lin, Xiaomin Bie, Xiansheng Zhang, Xiaohui Li, Yuan Chen, Xueyong Zhang, Cristobal Uauy, Fei Lu, Chengcai Chu, Zhixi Tian, Jun Xiao

**Affiliations:** ^1^ Laboratory of Advanced Breeding Technologies Institute of Genetics and Developmental Biology Chinese Academy of Sciences Beijing China; ^2^ University of Chinese Academy of Sciences Beijing China; ^3^ Yazhouwan National Laboratory Sanya China; ^4^ Key Laboratory for Enhancing Resource Use Efficiency of Crops in South China Ministry of Agriculture and Rural Affairs South China Agricultural University Guangzhou China; ^5^ John Innes Center Norwich Research Park Norwich UK; ^6^ Key Laboratory of Crop Gene Resources and Germplasm Enhancement Ministry of Agriculture/Institute of Crop Science Chinese Academy of Agricultural Sciences Beijing China; ^7^ State Key Laboratory of Wheat Improvement College of Life Sciences Shandong Agricultural University Tai'an China; ^8^ State Key Laboratory of Wheat Improvement Peking University Institute of Advanced Agricultural Sciences Weifang China; ^9^ CAS‐JIC Centre of Excellence for Plant and Microbial Science Institute of Genetics and Developmental Biology Chinese Academy of Sciences Beijing China

**Keywords:** breeding selection, geographic adaptation, pre‐harvest sprouting, wheat

## Abstract

Pre‐harvest sprouting (PHS) poses a major threat to wheat yield and quality, yet the genetic basis of seed dormancy underlying PHS resistance remains poorly understood. Here, through integrated genome‐wide association and transcriptomic analyses, we identify *TaMYB7‐A1* as a key regulator of seed dormancy and PHS resistance. *TaMYB7‐A1* encodes an R2R3‐MYB transcription factor that directly activates *TaABI5* to modulate abscisic acid (ABA) signaling and indirectly fine‐tunes ABA–gibberellin (GA) homeostasis to enforce dormancy. Evolutionary and haplotype analyses revealed that the superior allele, *TaMYB7‐A1^Hap−1^*, originated from wild einkorn and was introgressed into domesticated emmer and subsequently into modern bread wheat. A miniature inverted‑repeat transposable element (MITE) insertion in its promoter substantially elevates *TaMYB7‐A1* expression by increasing chromatin accessibility and facilitating the recruitment of upstream regulators, while two key amino acid substitutions (Gly23 and Gly92) strengthen its DNA‐binding and transcriptional activation capacity. Combinations of promoter and coding‐region variants generate graded PHS resistance across haplotypes, mirroring local adaptation to harvest‑season precipitation. Introgression of *TaMYB7‐A1^Hap−1^* into modern cultivar enhances PHS resistance without yield penalties. These findings elucidate a molecular and evolutionary framework for precipitation‐driven adaptation and provide a valuable genetic target for developing climate‐resilient wheat varieties.

## Graphical abstract

TaMYB7‐A1 directly activates TaABI5 to enhance ABA signaling and regulate ABA‐GA homeostasis, enforcing seed dormancy. Its superior allele, derived from wild einkorn introgression, harbors a MITE insertion that elevates expression and two amino acid substitutions that enhance transcriptional activity, collectively generating graded PHS resistance for local precipitation adaptation and offering a breeding target without yield penalty.

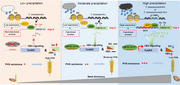

## 1 Introduction

Wheat (*Triticum aestivum* L.), a cornerstone of global agriculture, carries an evolutionary history marked by expansion and adaptation [1]. Its journey from the Fertile Crescent into diverse global environments required continuous genetic innovation to cope with climatic pressures, including varying temperature, photoperiod, and fluctuating harvest‐season precipitation [2, 3]. In this context, pre‐harvest sprouting (PHS)—the untimely germination of mature grain induced by rain—emerges as a critical vulnerability [4]. It severely compromises yield, nutritional quality, and end‐use functionality [4], with economic losses exceeding $1 billion annually [5]—a threat amplified by climate change‐induced weather instability [6].

At its core, PHS susceptibility reflects a breakdown in the balance between environmental cues and the intrinsic strength of seed dormancy [7]. Domestication often attenuated dormancy to ensure uniform germination [7, 8], yet this very trait is essential for resisting unpredictable harvest rains [9]. Thus, a central challenge in modern wheat breeding is the precision‐tuning of dormancy. Unlocking this balance requires a deep molecular understanding of dormancy regulation. In cereals, dormancy is primarily orchestrated by the antagonistic balance between abscisic acid (ABA), which enforces dormancy, and gibberellin (GA), which promotes germination [7, 8]. Key genes involved in ABA biosynthesis, perception, catabolism, and signal transduction, together with GA‐regulating genes, play central roles in this process. For instance, the stay‐green G protein in soybean modulates ABA accumulation in seeds through physical interactions with NINE‐CIS‐EPOXYCAROTENOID DIOXYGENASE 3 (NCED3) and PHYTOENE SYNTHASE (PSY), and its dormancy‐regulating function is conserved across multiple plant species [10]. In rice, SEED DORMANCY 6 (SD6) and INDUCER OF CBF EXPRESSION 2 (ICE2) co‐regulate seed ABA content and exhibit antagonistic effects on dormancy in a temperature‐dependent manner [11]. In wheat, however, the genetic and mechanistic basis of this trade‐off remains fragmented [11].

To date, only a limited number of wheat genes have been conclusively linked to PHS resistance in wheat, such as *VIVIPAROUS‐1* (*TaVP‐1*) and *PRE‐HARVEST SPROUTING 1* (*TaPHS1*, a synonym for *TaMFT*) that enhance dormancy by activating ABA signaling [12, 13], and *MYB DOMAIN PROTEIN 10* (*TaMYB10‐D1*) and ABSCISIC ACID INSENSITIVE4 (*TaABI4*) which confers resistance by upregulating the ABA biosynthesis genes [14, 15]. SIMILAR TO RCD‐ONE (TaSRO1) interacts with TaVP1 to suppress *TaPHS1* and *SEED DORMANCY GENE* (*TaSdr*), negatively regulating dormancy [16]. However, the translation of these genes into breeding has been faces two major constraints: pleiotropic effects on other essential agronomic traits, and the geographically restricted applicability of their superior haplotypes. For example, while *TaVP‐1* improves PHS‐resistance, it can adversely affects embryo development and plant structure [13]. Strong dormancy allele of *TaMFT‐3A* occurs at low frequency in modern wheat breeding lines [17], and the red grain color associated with the superior haplotype of *TaMYB10‐D1* and *DEHYDROFLAVONOL‐4 REDUCTASE* (*TaDFR*) is undesirable in Chinese breeding programs that prioritize white wheat for noodle and steamed bread production [14, 18].

Critically, wheat is cultivated across vast and highly heterogeneous agro‐climatic zones, where patterns of harvest‐season humidity and rainfall—the primary triggers of PHS—vary dramatically [1, 3]. This reality defines the essential criteria for the next generation of PHS‐resistance genes: they must confer strong, stable resistance without yield or quality penalties, and that are underpinned by allelic diversity suitable for tailoring adaptation to different agro‐climatic zones, particularly humidity and rainfall patterns during harvest. Ultimately, this raises a fundamental question: how does a polyploid crop like wheat fine‐tune such a key adaptive trait? The answer likely lies in the subtle, combinatorial variation within existing regulatory networks—variation shaped by evolutionary history, including introgression from wild relatives, and sculpted by environmental selection pressures [10, 11].

Here, we identify the R2R3‐MYB transcription factor *TaMYB7‐A1* as a key regulator of seed dormancy and PHS resistance in wheat. We show that natural variation at this locus, shaped by evolution and breeding, fine‐tunes dormancy through effects on ABA signaling and hormone homeostasis. A gradient of resistance is explained by haplotypes combining *cis*‐regulatory and coding variants. Evolutionary analysis reveals introgression of a strong‐dormancy haplotype from wild relatives, whose distribution mirrors adaptation to harvest‐season precipitation. Introduction of this elite haplotype enhances PHS resistance without a yield penalty, providing both a breeding target and a framework for engineering climate‐resilient wheat.

## 2 Results

### 2.1 *TaMYB7‐A1* is Associated with PHS‐Resistance in Wheat

**FIGURE 1:**
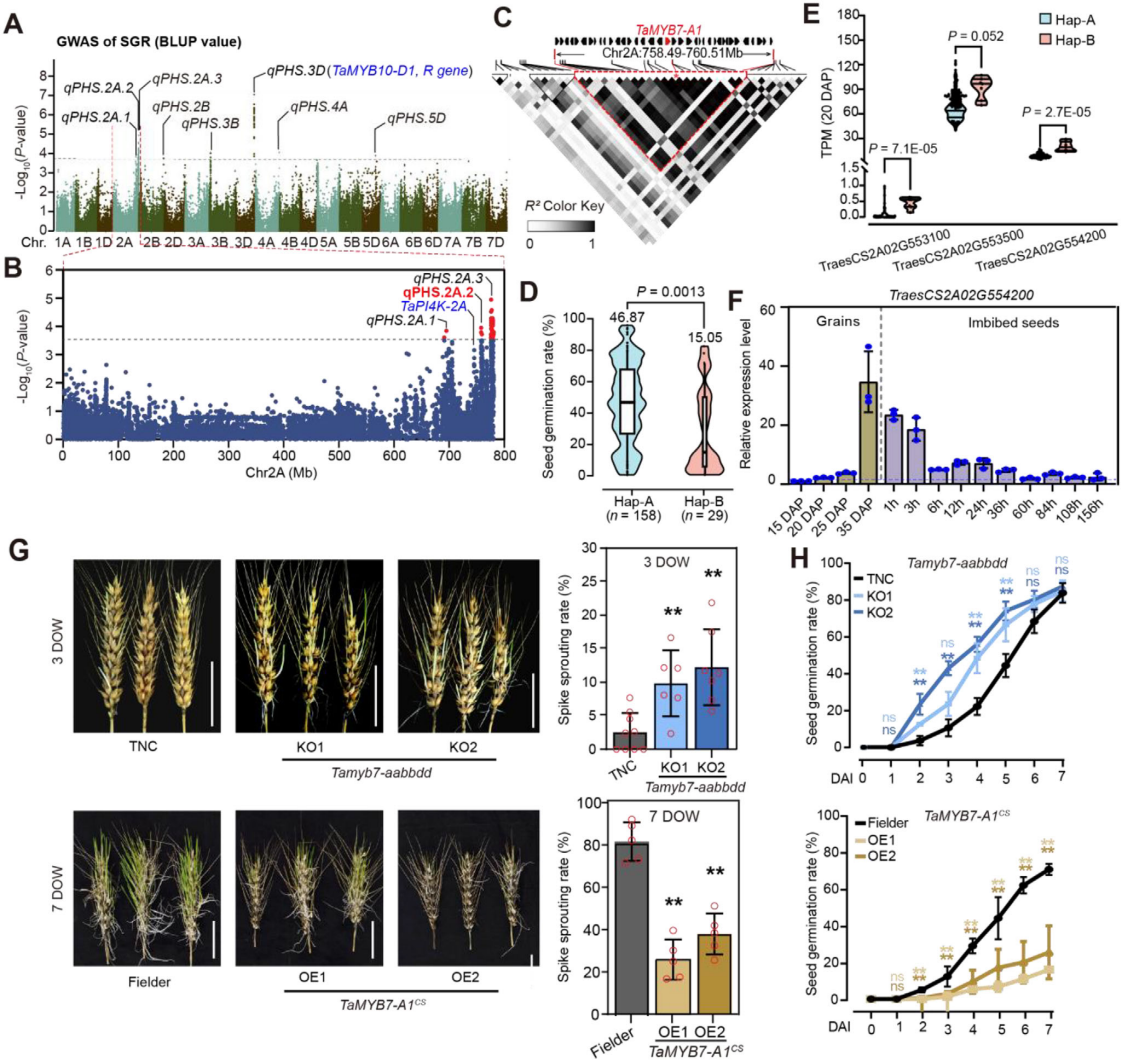
Genome‐wide association analysis unravels the genetic basis of pre‐harvest sprouting resistance in wheat. (A) Manhattan plot illustrating statistically significant association signals for best linear‐unbiased‐prediction (BLUP) values of seed‐germination rate (SGR) in the GWAS panel. Horizontal dashed line indicates the genome‐wide suggestive statistical‐significance threshold (*P* = 3.7E‐04) for marker–trait associations. Stable association signals for at least two environments are labeled, with known PHS‐resistance regulator *TaMYB10‐D1* marked in blue. (B) Local Manhattan plot showing significant peaks on chromosome 2A. The peak SNPs associated with SGR are marked in red and known PHS‐resistance regulators labeled in blue. (C) Heatmap of linkage disequilibrium (LD) between SNPs within the 3‐Mb region surrounding the peak marker for *qPHS.2A.2*. The intensity of color from white to black represents the range of *r^2^* values from 0–1. The LD block for the peak marker was indicate with a red inverted triangle dashed box, and *TaMYB7‐A1* is denoted in red. (D) Violin plot of SGR among wheat accessions with haplotypes Hap‐A and Hap‐B defined by SNPs in the *qPHS2.2* LD block. Wilcoxon rank‐sum test was used to determine the statistical significance. (E) Expression levels for candidate genes among wheat accessions carrying different *qPHS2.2* haplotypes. RNA‐seq data of grains harvested at 20 days after pollination (DAP) from 102 varieties were used for analysis. Statistical significance between means of the two groups was determined by Wilcoxon rank‐sum test. (F) RT‐qPCR of *TraesCS2A02G554200* (*TaMYB7‐A1*) dynamics during grain development (brown bars) and seed imbibition (purple bars). *TaTubulin* was used as the internal control, and expression levels were normalized with grains at 15 DAP set to 1 (blue dashed line). Data are represent mean ± S.D. of three biological replicates. (G) Germination performance of spike sprouting rate in*TaMYB7‐A1* overexpression (OE) lines versus wild‐type Fielder (the OE control), and *TaMYB7* knockout mutants (KO) versus TNC (the KO control). TNC refers to the negative control lines isolated from KO lines. Photos were taken at 7 days of wetting (DOW) for OE lines and 3 DOW for knockout lines. Scale bar = 5 cm. Data represent mean ± S.D. from 4–7 spikes. ^**^, *P* < 0.01 (Student's *t*‐test). (H) Germination time course of OE lines versus Fielder, and KO lines versus TNC over 7 days after imbibition (DAI). Data represent mean ± S.D. of three biological replicates (∼50 seeds per replicate). Significance at each time point (Student's *t*‐test, with corresponding control) is indicated by color‐matched markers: ^**^, *P* < 0.01; ns, *P* ≥ 0.05.

To dissect the genetic basis of PHS‐resistance and identify elite alleles, we performed a genome‐wide association study (GWAS) on 187 genetically and geographically diverse wheat accessions representing a wide spectrum of seed germination rate (SGR) but minimal population structure (Figures S1 and S2 and Table S1). We detected eight loci significantly associated with best linear‐unbiased‐prediction (BLUP) values of SGR (−Log_10_(*P*) > 3.7; Figure 1A; Table S2), which exhibited additive effects such that a higher number of resistance alleles correlated with lower SGR values (Figure S3 and Table S3). Among them, *qPHS.3D* (corresponding to the red grain color gene *TaMYB10‐D1*) [14] and a QTL cluster at the terminal region of chromosome 2A were the most prominent (Figure 1A; Figure S4). The 2A cluster contained the known PHS‐resistance gene *TaP14K‐2A* [19] along with three novel loci: *qPHS.2A.1, qPHS.2A.2*, and *qPHS.2A.3* (Figure 1B). Of these, *qPHS.2A.2*, and *qPHS.2A.3* were consistently detected across BLUP values and at least two out of three annual GWAS runs, underscoring their robust association with PHS‐resistance, whereas *qPHS.2A.1* was not (Figure 1A; Figure S4). Consequently, we focused subsequent analyses on *qPHS.2A.2* and *qPHS.2A.3*.

At *qPHS.2A.3* (Chr 2A: 776.49–778.42 Mb), we prioritized *TaAIRP2‐A1*, a homolog of *Arabidopsis* RING E3 ubiquitin‐ligase AIRP2 involved in ABA signaling [20], from among 29 annotated genes based on its expression profile and functional relevance (Figure S5A,B). As the only confident candidate showing seed‐specific expression, particularly during the late stages of endosperm development, *TaAIRP2‐A1* was upregulated during seed maturation but declined rapidly upon imbibition, consistent with a role in dormancy regulation (Figure S5C and Table S4). Although *TaAIRP2‐A1* haplotypes varied in germination, their expression was similar, suggesting functional divergence may primarily stems from amino acid variation (Figure S5D,E). Overexpressing the PHS‐resistant *TaAIRP2‐A1^Hap−II^* allele from Chinese Spring (CS) in the moderately resistant wheat variety Fielder significantly reduced the spike sprouting rate (SSR) from 81.44% (Fielder) to 44.33% (OE‐1, *p* = 0.0007) and 22.63% (OE‐2, *p* < 0.0001), demonstrating markedly enhanced PHS‐resistance (Figure S6A,B). However, transgenics exhibited reduced grain size, grain weight, and plant height (Figure S6C), diminishing breeding utility.

The *qPHS.2A.2* locus spans a 2.02‐Mb linkage‐disequilibrium (LD) block (Chr 2A: 758.49–760.51 Mb) containing 37 high‐confidence genes (Figure 1C). Single‐nucleotide polymorphism (SNP) variation within this region divided accessions into two *qPHS.2A.2* haplotypes (Hap‐A and Hap‐B) with contrasting SGR (46.87% versus 15.05%, *P* = 0.0013, Figure 1D). Among the expressed candidates (TPM > 1 across tissues), three genes, including *TraesCS2A02G554200* exhibited strong grain‐preferential expression, particularly during late development when dormancy is established [21] (Figure S7A–C and Table S5). For *TraesCS2A02G554200*, expression analysis across 102 accessions [22] revealed significant *qPHS.2A.2* haplotype‐dependent differences at 20 days after pollination (DAP) (Figure 1E; Table S6). Additionally, RT‐qPCR showed its upregulation during seed maturation and rapidly downregulation upon imbibition (Figure 1F). In contrast, *TraesCS2A02G553500* showed no significant *qPHS.2A.2* haplotype‐dependent differences (Figure 1E), and *TraesCS2A02G553100* exhibited no consistent association with dormancy (Figure S7D). Accordingly, *TraesCS2A02G554200*, an ortholog of rice *MYB transcription factor 6* [23], was identified as the putative causal gene for *qPHS.2A.2* and was designated *TaMYB7‐A1*, following the established systematic nomenclature for wheat R2R3‐MYB family [24].

To validate TaMYB7‐A1 function, we generated knockout (KO) and overexpression (OE) transgenic lines in Fielder (Figure S8A,B), which was selected for its high transformability and moderately PHS‐resistant phenotype, facilitating the detection of both enhanced and reduced PHS‐resistance. The coding sequence from Chinese Spring (CS, carring *qPHS.2A.2^Hap‐B^*) was used for overexpression. Two independent triple KO lines of all three homeologs showed accelerated germination compared to the transgenic negative control (TNC), with higher SSR at 3 days of wetting (DOW), and increased SGR at 2–5 days after imbibition (DAI) (Figure 1G,H; Figure S8C). The observed SSR difference diminished by 7 DOW as values approached saturation (∼80–95%) (Figure S8C), indicating that TaMYB7‐A1 acts primarily during the early phase of dormancy maintenance. In contrast, *TaMYB7‐A1^CS^* OE lines displayed significantly stronger PHS‐resistance than Fielder (Figure 1G,H; Figure S8D). Notably, *TaMYB7‐A1^CS^* overexpression did not affect germination of dormancy‐released seeds (Figure S8E), nor alter key agronomic traits such as plant height, spike length, spikelet number, and grain morphology (Figure S9). Conversely, KO lines exhibited pleiotropic effects, including reduced grain weight, shorter spikes, and decreased plant height (Figure S9).

Collectively, these findings demonstrate that elevated *TaMYB7‐A1* expression enhances PHS‐resistance without compromising yield or quality, while its complete loss may disrupt normal development, highlighting this gene as a promising target for breeding PHS‐resistant wheat.

### 2.2 TaMYB7‐A1 Enhances PHS‐Resistance by Directly Promoting ABA Signaling and Indirectly Modulating ABA–GA Homeostasis

To dissect the regulatory pathways of TaMYB7‐A1, we performed RNA‐seq on developing seeds of Fielder and the *TaMYB7* KO line at 15, 25, and 35 DAP, covering the critical transitions of seed dormancy from initiation to maintenance. Principal component analysis revealed clear separation of transcriptomes by both genotype and stage, indicating stage‐specific transcriptional reprogramming in the absence of TaMYB7‐A1 (Figure S10A).

**FIGURE 2:**
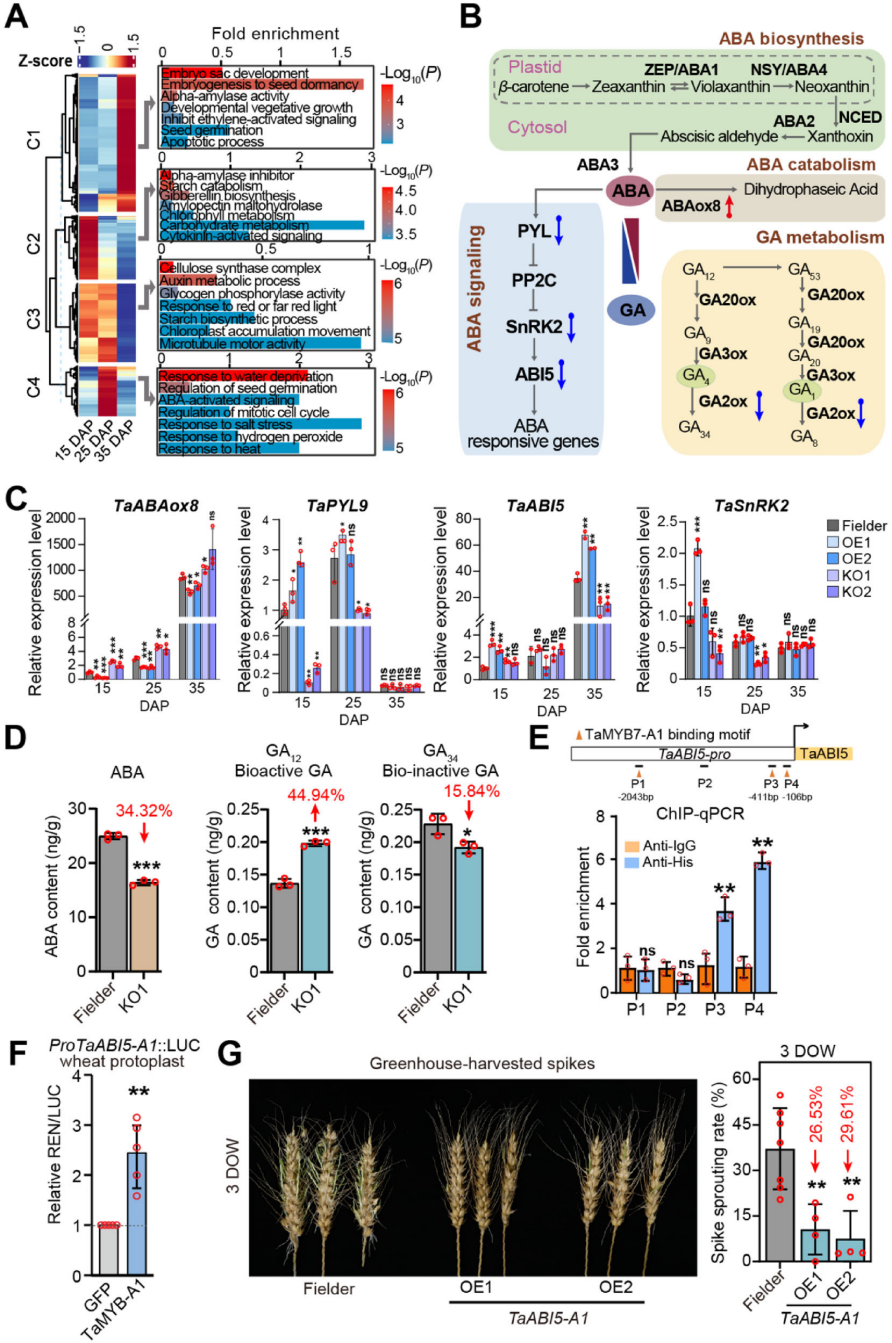
*TaMYB7‐A1* enhances PHS‐resistance by modulating ABA signaling and ABA–GA homeostasis in grains. (A) Hierarchical clustering of differentially expressed genes (DEGs) across three developmental stages (15, 25, and 35 DAP) in Fielder. Gene Ontology (GO) enrichment analysis was performed for each co‐expression cluster. (B) Key genes involved in ABA biosynthesis, signaling and metabolism, and in GA conversion. Red and blue arrows indicate DEGs up‐ and down‐ regulated in KO1 compared to Fielder, respectively. (C) RT‐qPCR validation of selected genes related to ABA metabolism and signaling in Fielder, *TaMYB7‐A1* overexpression lines and *TaMYB7* knockout lines. *TaTubulin* was used as the internal control, and expression levels were normalized to Fielder at 15 DAP (set to 1). Data represent mean ± S.D. of three biological replicates. ^*^, *P* < 0.05; ^**^, *P* < 0.01; ^***^, *P* < 0.001, ns, *P* ≥ 0.05 (Student's *t*‐test). (D) ABA, GA_12_ and GA_34_ contents in 35 DAP grains from Fielder and KO1. Data represent mean ± S.D. of three biological replicates. ^*^, *P* < 0.05, ^***^, *P* < 0.001 (Student's *t*‐test). (E) TaMYB7‐A1 binds to the *TaABI5* promoter. Chromatin immunoprecipitation followed by qPCR (ChIP‐qPCR) was performed in wild‐type (WT) and *TaMYB7‐A1* overexpression (OE) lines using an anti‐His antibody; non‐specific rabbit IgG served as the control. The enrichment of each target genomic fragment (P1–P3, as shown in the schematic) was calculated as percentage of input (% Input) and is presented as fold enrichment relative to the corresponding IgG control. Data are shown as mean ± S.D. of three independent biological replicates (individual points). ^*^, *P* < 0.05; ^**^, *P* < 0.01; ns, *P* ≥ 0.05 (Student's *t*‐test). (F) TaMYB7‐A1 activates the *TaABI5* promoter. Dual‐luciferase reporter assays were performed in wheat protoplast. The promoter regions of *TaABI5‐A1* were fused to firefly luciferase reporter and co‐expressed with either TaMYB7‐A1 (effector) or GFP (negative control). The relative LUC/REN ratios was shown. For each biological replicate, the LUC/REN ratios were normalized to GFP control transfections (set to 1). Data are presented as mean ± S.D. of five independent transfections. ^**^, *P* < 0.01 (Student's *t*‐test). (G) Spike sprouting performance of Fielder and *TaABI5* overexpression lines after 3 days of wetting (DOW). Greenhouse‐harvested spikes from Fielder and homozygous T_2_ transgenic lines were used to evaluate spike sprouting rate. Data represent mean ± S.D. from 4–7 spikes. ^**^, *P* < 0.01 (Student's *t*‐test).

Temporal clustering of Fielder expression profiles identified four stage‐specific transcriptional modules: (i) 15 DAP–high genes enriched for GA‐dependent processes, cytokinin‐activated signaling and carbohydrate metabolism (C2); (ii) 25 DAP–high genes linked to ABA signaling and germination control (C4); (iii) 35 DAP–high genes associated with dormancy maintenance and auxin signaling (C1); and (iv) genes with sustained expression across 15–25 DAP (C3), enriched for starch biosynthesis and auxin regulation (Figure 2A; Table S7). These dynamics align with physiological transitions from grain filling to dormancy [21, 25].

Comparative analysis revealed 33,442 differentially expressed genes (DEGs) potentially regulated by TaMYB7‐A1 (Figure S10B and Table S8). Integration with seed‐specific Assay for Transposase‐Accessible Chromatin with high throughput sequencing (ATAC‐seq) [26] and motif data refined this to 18,147 high‐confidence candidates harboring putative TaMYB7‐A1 binding sites (Figure S10B,C and Table S9). Many known dormancy‐ and ABA‐related genes were included (Figure S10D,E). Hormone pathway analysis reveals a coherent ABA homeostasis disruption cascade in the KO line: ABA biosynthetic genes (*TaNCED1*, *TaABA1, TaABA2*, and *TaABA3*) maintained stable expression, whereas the catabolic gene *TaABAox8* was significantly upregulated (Figure 2B and C). RT‐qPCR further validated the opposite expression trends of these genes in multiple OE and KO lines compared to Fielder (Figure 2C; Figure S11). Moreover, direct quantification confirmed a 34.32% reduction of ABA in 35 DAP KO seeds, consistent with the biosynthetic/catabolic related genes’ imbalance expression (Figure 2D). Under 50 µM ABA treatment, the OE line exhibited a stronger inhibition of seed germination than Fielder, confirming its ABA‐hypersensitive phenotype (Figure S12A). Notably, dual‐luciferase assays indicated that TaMYB7‐A1^CS^ could not directly transactivate *TaABAox8‐A1*—suggesting an indirect on ABA catabolism—the observed gene upregulation likely results from perturbed ABA signaling (Figure S12B). Uncovering the intermediary regulators linking these events remains a key objective for future studies.

For GA metabolism, expression analysis revealed that among the tested GA biosynthetic genes, only GA catabolic gene *TaGA_2_ox* expression consistently correlated with *TaMYB7‑A1* activity, with a marked downregulation in KO lines, while other biosynthesis and signaling genes remained largely unaffected (Figure 2B; Figure S11). Although bioactive GA_1_, GA_3_, GA_4,_ and GA_7_ were not detected in mature seeds [27], we observed a marked shift toward precursor accumulation (GA_12_, GA_53_, and GA_19_) accompanied by reduced derivatives such as GA_34_ (Figure 2D; Figure S12C,D). Together, these results indicate that TaMYB7‐A1 fine‐tunes GA homeostasis not through broad pathway regulation, but specifically by enhancing TaGA2ox‐mediated catabolism, thereby lowering active GA levels and shifting the ABA/GA balance toward dormancy.

Beyond hormone metabolism, ABA signaling components were also modulated. Key components including *TaPYL9* (*TraesCS7A02G358200*, *TraesCS7B02G269600*, *TraesCS7D02G364600*), *TaSnRK2* (*TraesCS1B02G157800*, *TraesCS1A02G358300*, *TraesCS1D02G082600*) and *TaABI5* (*TraesCS3A02G371800*, *TraesCS3B02G404200*, *TraesCS3D02G364900*) were downregulated in KO lines, accompanied by moderate but non‐significant reductions of *TaPP2C* (TraesCS6A02G193100, TraesCS6B02G229600, TraesCS6D02G181000) expression (Figure 2B,C; Figure S11). In silico motif analysis and Electrophoretic mobility shift assay (EMSA) confirmed that TaMYB7‐A1^CS^ direct binds to conserved CATAA motifs within *TaABI5* and *TaPYL9* promoters in vitro (Figure S13A–C). Consistent with this, dual‐luciferase reporter assays in *N. benthamiana* leaves confirmed that TaMYB7‐A1^CS^ activates both promoters (Figure S13D,E). In light of the narrow expression window of *TaPYL9*, we prioritized *TaABI5* for downstream functional analysis (Figure C). Chromatin immunoprecipitation followed by qPCR (ChIP–qPCR) confirmed specific binding of TaMYB7‐A1 at the *TaABI5‐A1* promoter in developing wheat seeds (15 DAP) (Figure 2E), and dual‐luciferase reporter assays in wheat protoplasts further verified its transactivation activity (Figure 2F).

To validate the functional hierarchy, *TaABI5‐A1* (*TraesCS3A02G371800*) overexpression lines were generated in the Fielder background, and greenhouse‐harvested T_2_ generation transgenic‐positive individuals exhibited significantly enhanced PHS‐resistance (26.53% and 29.61% reduction of SSR in OE lines compared to control), confirming that *TaABI5‐A1* acts downstream of TaMYB7‐A1 in controlling PHS (Figure 2G). Collectively, these results demonstrate that TaMYB7‐A1 directly enhances ABA signaling by upregulating key factors such as TaABI5.

To explore upstream regulation of TaMYB7‐A1, we leveraged ATAC‐seq data from developing Chinese Spring seed [26], which revealed a conserved proximal accessible region in its promoter containing motifs for ABI4, HBs, and MYBs (Figure S14A). Among these, expression of *TaABI4* was significantly positively correlated with that of *TaMYB7‐A1*, whereas *TaHB20* exhibited a significant negative correlation with *TaMYB7‐A1* (Figure S14B and Table S10). Dual‐luciferase assays further demonstrated that TaABI4 was activated, while TaHB20 repressed, *TaMYB7‐A1^CS^* promoter activity (Figure S14C). Moreover, EMSA confirmed that the TaABI4 protein directly binds to the *TaMYB7‐A1* promoter region in vitro (Figure S14D). Reduced *TaMYB7‐A1* expression in multiple *Taabi4* ethyl methane sulfonate mutants in the KN9204 background [28] further supports TaABI4 as an upstream activator (Figure S14E).

Together, these findings place TaMYB7‐A1 within a regulatory module, working in conjunction with TaABI4 and TaABI5 to enhance seed dormancy and PHS resistance by coordinating the regulation of ABA signaling and ABA–GA homeostasis.

### 2.3 Natural Variations in TaMYB7‐A1 Confer a Gradient of PHS‐Resistance

**FIGURE 3:**
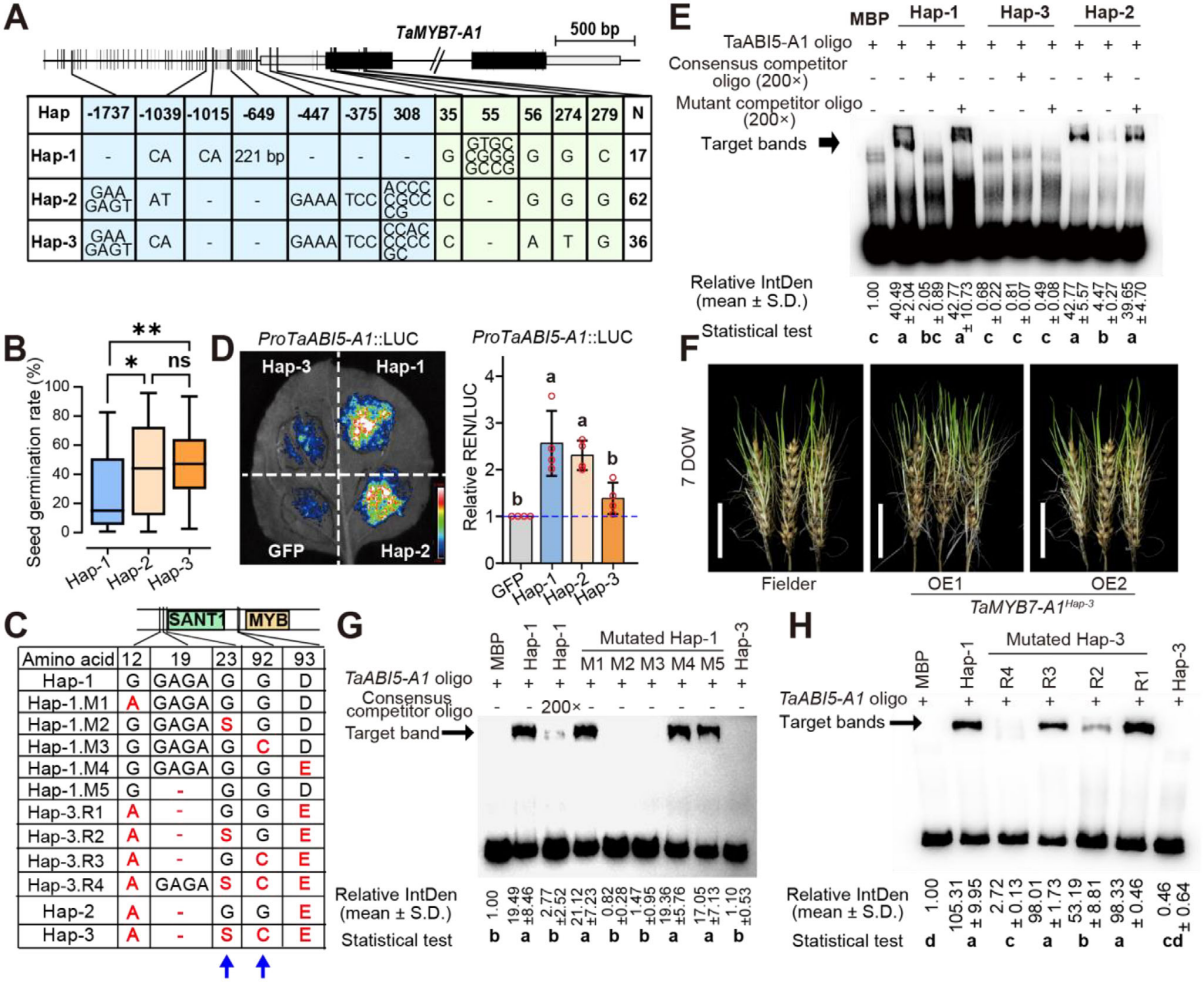
Natural variations in *TaMYB7‐A1* underlies PHS resistance, with Gly23 and Gly92 essential for its function (A) Summary of polymorphisms defining the major *TaMYB7‐A1* haplotypes. Only promoter InDels and variations causing amino acid changes are detailed. N indicates the number of sequenced varieties carrying each haplotype. (B) Germination rate of wheat accessions grouped by *TaMYB7‐A1* haplotypes. Best linear unbiased prediction values of seed germination rate from GWAS panel are shown. The box denotes the 25^th^, median, and 75^th^ percentiles, while the whiskers indicate the 1.5× interquartile range.^*^, *P* < 0.05; ^**^, *P* < 0.01 (Wilcoxon rank‐sum test). (C) Schematic of amino‐acid variations in TaMYB7‐A1 haplotypes. Recombinant TaMYB7‐A1–MBP proteins were generated for natural haplotypes and for engineered variants of TaMYB7‐A1^Hap−1^ (M1–5) and TaMYB7‐A1^Hap−3^ (R1–4). Variation sites are highlighted in red font; key residues are marked with blue arrows. (D) Dual‐luciferase reporter assays in *N. benthamiana* leaves. The *TaABI5‐A1* promoter was fused to luciferase and co‐expressed with indicated TaMYB7‐A1 haplotype or GFP (negative control). LUC/REN ratios were normalized to GFP (set to 1). Different letters indicating significance at *P* < 0.05 (Tukey's HSD multiple comparisons test). (E) EMSA showing binding of recombinant TaMYB7‐A1–MBP haplotypes to a *TaABI5‐A1* promoter‐fragment probe containing the GATAA motif. “+” and “–” denote the presence/absence of the probe or protein, respectively. 200× denotes 200‐fold molar excess of the unlabeled competitor and mutant probe. The arrow marks the protein‐bound probe. A representative gel is shown. The bar graph (mean ± S.D., *n* = 3 technical replicates) quantifies bound probe, normalized to the MBP‐only control (set to 1). Different letters indicates significance at *P* < 0.05 (Tukey's HSD multiple comparisons test). (F) Germination performance of Fielder and *TaMYB7‐A1^Hap−3^* OE lines after 7 days of wetting (DOW). Scale bar, 5 cm. (G, H) EMSA assessing the contribution of key residues to DNA binding for TaMYB7‐A1^Hap−1^ (G) and TaMYB7‐A1^Hap−3^ (H). “+” and “–” denote the presence and absence of the probe or protein, respectively. The arrow depicts the bound probe. 200× denotes 200‐fold excess of unlabeled wild‐type probe. Average band ratio displayed below the gel. The bar graph (mean ± S.D., *n* = 3 technical replicates) shows relative band intensity normalized to the MBP control (set to 1). Different letters indicate significant differences among variants (Tukey's HSD test, *P* < 0.05).

To capture the natural genetic variants underlying phenotypic variation across *TaMYB7‐A1* alleles, we resequenced its genic region (including the coding sequence and ∼2.5 kb promoter) in 115 wheat accessions selected from two complementary diversity panels—41 from our GWAS panel and 74 from the Chinese wheat mini‐core collection—ensuring a broad representation of allelic diversity (Table S11). We detected eleven SNPs and one Insertion/Deletion (InDel) in the coding region, along with seven InDels and multiple SNPs in the promoter (Figure 3A; Table S11). These variations delineated three major gene haplotypes of *TaMYB7‐A1*: *TaMYB7‐A1^Hap−1^*, *TaMYB7‐A1^Hap−2^*, and *TaMYB7‐A1^Hap−3^* (abbreviated Hap‐1, Hap‐2, Hap‐3), which showed statistically significant differences in SGR (Figure 3A, B). Among them, Hap‐1 conferred the strongest PHS‐resistance, significantly outperforming both Hap‐2 and Hap‐3; Hap‐3 was the most susceptible, whereas Hap‐2 exhibited an intermediate phenotype that was statistically closer to Hap‐3.

Five polymorphisms within the coding sequence resulted in four amino acid substitutions and a Gly‐Ala‐Gly‐Ala InDel (Figure 3A‐C). Functional assays revealed a clear functional hierarchy: both Hap‐1 and Hap‐2 proteins bound to and activated the *TaABI5* promoter in vitro and *in planta*, whereas the Hap‐3 protein lacked detectable DNA‐binding and transactivation activity (Figure 3D,E; Figure S15A,B), suggesting it is a non‐functional allele. This functional divergence was consistently reflected in vivo: overexpression of Hap‐3 failed to alter the sprouting phenotype (SSR or SGR) or upregulate *TaABI5* expression (Figure 3F; Figure S15D–F), whereas Hap‐1 overexpression lines showed significantly reduced sprouting rate (Figure 1G and H), thereby confirming the loss‐of‐function nature of Hap‐3.

To pinpoint the causal residues responsible for functional variation, we generated recombinant proteins corresponding to Hap‐1 and Hap‐3 and constructed a series of site‐directed mutants in the Hap‐1 background (M1–M5, Figure 3C). Combined EMSA and dual‐luciferase assays revealed that Gly23 and Gly92 are indispensable for DNA binding to the CATAA motif and for transcriptional activation of the *TaABI5* promoter. Specifically, the M2 (Gly23Ser) and M3 (Gly92Cys) mutations completely abolished DNA‐binding activity, whereas mutations at other sites (M1, M4, and M5) had no detectable effect (Figure 3G; Figure S15G). These two residues distinguish the functional Hap‐2 from the non‐functional Hap‐3 allele. Functional complementation further demonstrated that simultaneous reversion of both substitutions in Hap‐3 fully restored DNA‐bindingy, whereas individual single revertant conferred only partially recovery, indicating an additive contribution of each glycine residue (Figure 3H). Notably, the homeologous copies TaMYB7‐B1 and TaMYB7‐D1 lack the critical Gly23 despite otherwise conserved sequences, which likely explains their markedly weaker activation of *TaABI5* compared with TaMYB7‐A1 (Figure S16A–C), reflecting partial functional divergence among subgenomic copies.

Taken together, natural polymorphisms in TaMYB7‐A1, particularly Gly23Ser and Gly92Cys, directly alter its DNA‐binding affinity and transcriptional activation of ABA signaling components such as *TaABI5*. These molecular changes underpin haplotype‐dependent variation in dormancy strength and PHS‐resistance.

### 2.4 A MITE Insertion Enhances TaMYB7‐A1 Expression in Hap‐1 Accessions

**FIGURE 4:**
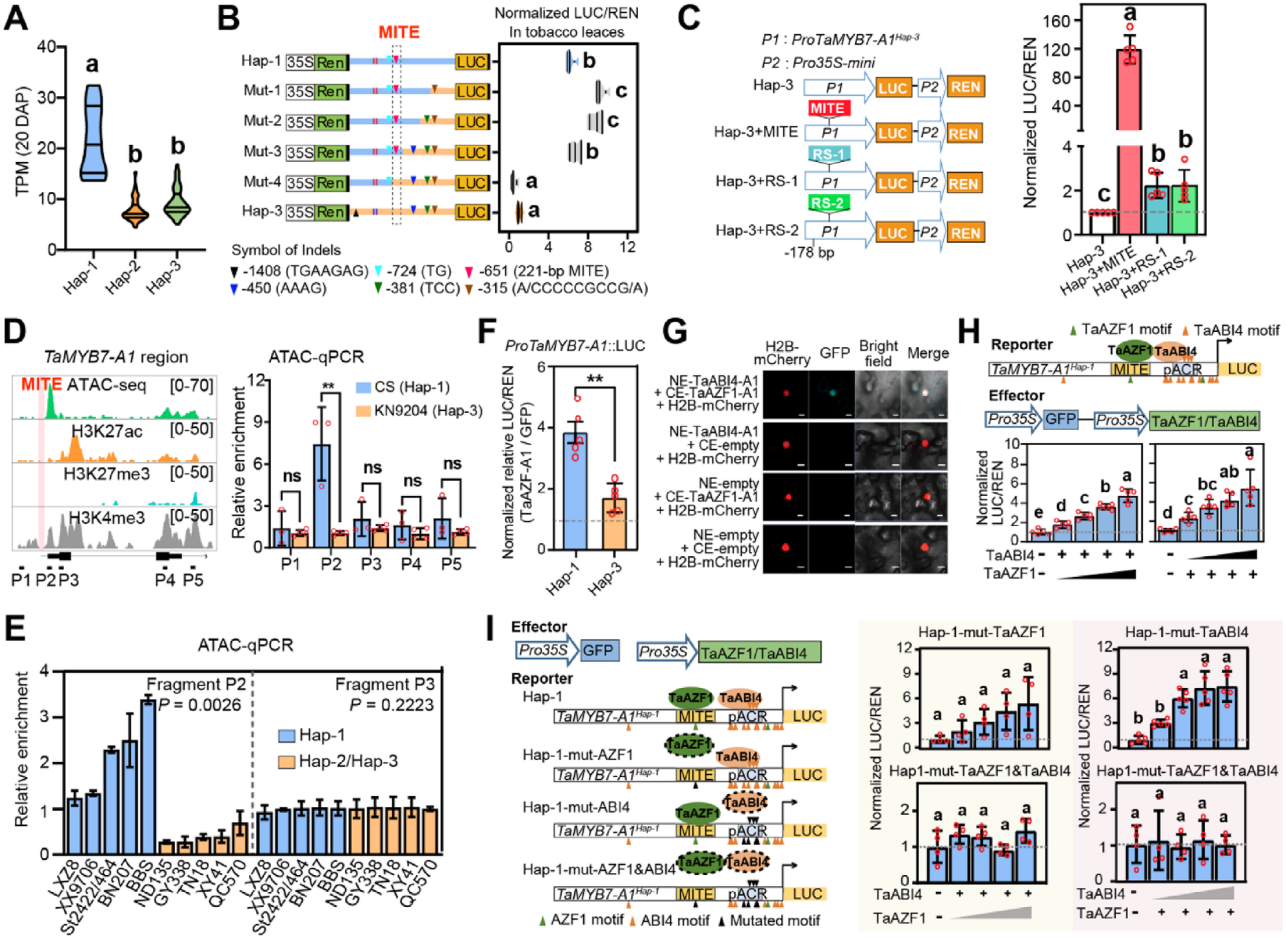
A MITE insertion boosts *TaMYB7‐A1^Hap−1^* expression. (A) *TaMYB7‐A1* expression levels in 20 days after pollination (DAP) grains for accessions with Hap‐1, Hap‐2 and Hap‐3 haplotypes. Different letters indicate a significant difference at *P* < 0.05 (Tukey's HSD multiple‐comparisons test). (B) Dual‐luciferase reporter assays for *TaMYB7‐A1* promoter activity in *N. benthamiana* leaves. Schematics depict promoter fragments from Hap‐1 (blue) and Hap‐3 (orange); Inverted triangles mark InDel positions. The 221‐bp MITE, indicated by a dashed frame, is the only difference between Mut‐3 and Mut‐4. Data are mean ± S.D of five biological replicates. Different letters indicate significant differences at *P* < 0.05 (Tukey's HSD multiple comparisons test). (C) Reporter assays comparing the Hap‐3 promoter with the same promoter containing the MITE or a random sequence of equal length inserted at the native MITE position.LUC/REN values were normalized to Hap‐3 (set to 1, gray dashed line). Different letters indicate significant differences at *P* < 0.05 (Tukey's HSD multiple comparisons test). (D) Chromatin accessibility and histone‐modification profiles across the TaMYB7‐A1 region in Chinese Spring developing seeds at 22 DAP. The pink box indicates the MITE insertion site. P1–5 denote to regions analyzed by ATAC‐qPCR evaluation. Data represent mean ± S.D. of three biological replicates. ns, *P* ≥ 0.05; ^**^, *P* < 0.01 (Student's *t*‐test). (E) ATAC‐qPCR assessing chromatin accessibility at the promoter (P2) and first exon (P3) in ten cultivars carrying different *TaMYB7‐A1* haplotypes. Hap‐1 accessions are shown in blue, while Hap‐2 or Hap‐3 in orange columns. Student's *t*‐test was used for the statistical significance between Hap‐1 and Hap‐2/Hap‐3. (F) Reporter assays testing activation of the Hap‐1 or Hap‐3 promoter by TaAZF1‐A1. Promoter regions from *TaMYB7‐A1^Hap−1^* and *TaMYB7‐A1^Hap−3^* were fused to firefly luciferase reporter and co‐expressed with either TaAZF1‐A1 (effector) or GFP (negative control). LUC/REN values are normalized to rhe GFP control (set to 1, gray dashed line). ^**^, *P* < 0.01 (Student's *t*‐test). (G) Bimolecular fluorescence complementation (BiFC) in *N. bethamiana* leaves showing the physical interaction between TaAZF1 and TaABI4. H2B‐mCherry marks nuclear. Scale bar, 10 µm. (H) Reporter assays measuring synergistic activation of the Hap‐1 promoter by TaAZF1 and TaABI4. Effectors were co‐expressed in the indicated ratios, indicated by “‐”, “+”, or gradient black triangles. Relative LUC/REN values were normalized with the GFP control (set to 1). Different letters indicate significant differences at *P* < 0.05 (Tukey's HSD multiple comparisons test). (I) Reporter assays using Hap‐1 promoter reporters with mutations in the TaAZF1 and/or TaABI4 binding sites, co‐expressed with TaAZF1 and TaABI4 effectors. Dual‐luciferase reporter assays were conducted in *N. benthamiana* leaves using the effector constructs Pro35S::TaAZF1 and Pro35S::TaABI4 (co‐transformed in different proportions as indicated) alongside a series of ProTaMYB7‐A1::LUC reporters bearing mutations in the TaAZF1 and/or TaABI4 binding motifs. Within each biological replicate, LUC/REN ratios were normalized to a GFP controls (set to 1). Different letters indicate significance at *P* < 0.05 (Tukey's HSD multiple‐comparisons test).

In addition to coding differences, *TaMYB7‐A1* haplotypes harbour distinct promoter variants (Figure 3A; Table S11). Pan‐transcriptome data from 20 DAP grains [22] and RT‐qPCR in 25 DAP and 35 DAP grains confirmed that Hap‐1, which confers stronger PHS‐resistance, exhibits significantly higher *TaMYB7‐A1* expression than Hap‐2 and Hap‐3 (Figure 4A; Figure S17).

Promoter‐swapping experiments pinpointed a 221‐bp insertion unique to Hap‐1 as a causal element (Figure 4B; Figure S18). This insertion was identified as a Stowaway‐like miniature inverted‐repeat transposable element (MITE) [29]. Dual‐reporter assays in wheat protoplasts and tobacco leaves demonstrated that the MITE substantially enhanced promoter activity compared to Hap‐3 or random sequence insertions (Figure 4C; Figure S19A,B). Its enhancer‐like role required the intact MITE sequence and was sensitive to its distance from the transcription start site (Figure S19C,D). Unlike many MITEs that influence expression through DNA methylation, the MITE in TaMYB7‐A1 showed no differential methylation around its insertion site, suggesting a methylation‐independent mode of regulation (Figure S20).

MITE insertions are known to modulate local chromatin states [30]. Epigenomic profiling of developing endosperm [26] revealed that the MITE is situated within a region of open chromatin, marked by active histone modifications (H3K27ac and H3K4me3) (Figure 4D). Accordingly, ATAC‐qPCR confirmed significantly higher chromatin accessibility in Hap‐1 compared with Hap‐2 and Hap‐3 accessions (Figure 4D and E). Motif analysis predicted several transcription factor binding sites within the MITE, including an additional TaAZF1‐binding motif unique to Hap‐1 (Figure S21A). Co‐expression analysis and dual‐luciferase assays demonstrated that TaAZF1 strongly activates Hap‐1 but only weakly activates Hap‐3 promoter (Figure 4F; Figure S21B–D). Furthermore, protein interaction assays revealed that TaAZF1 physically interacts with TaABI4 in the nucleus (Figure 4G; Figure S21E), and the two factors act synergistically to transactivate the Hap‐1 reporter (Figure 4H). Disruption of either the TaAZF1 or TaABI4 binding site reduced this activation, and the combined mutation nearly abolished transcriptional activation of *TaMYB7‐A1* (Figure 4I).

Together, these results demonstrate that the Hap‐1‐specific MITE acts as a functional enhancer‐like element by promoting chromatin accessibility and recruiting transcription factors such as TaAZF1, thereby elevating *TaMYB7‐A1* expression and boosting PHS‐resistance.

### 2.5 TaMYB7‐A1^Hap−1^ Derives from Einkorn Introgression Rather Than Urartu

**FIGURE 5:**
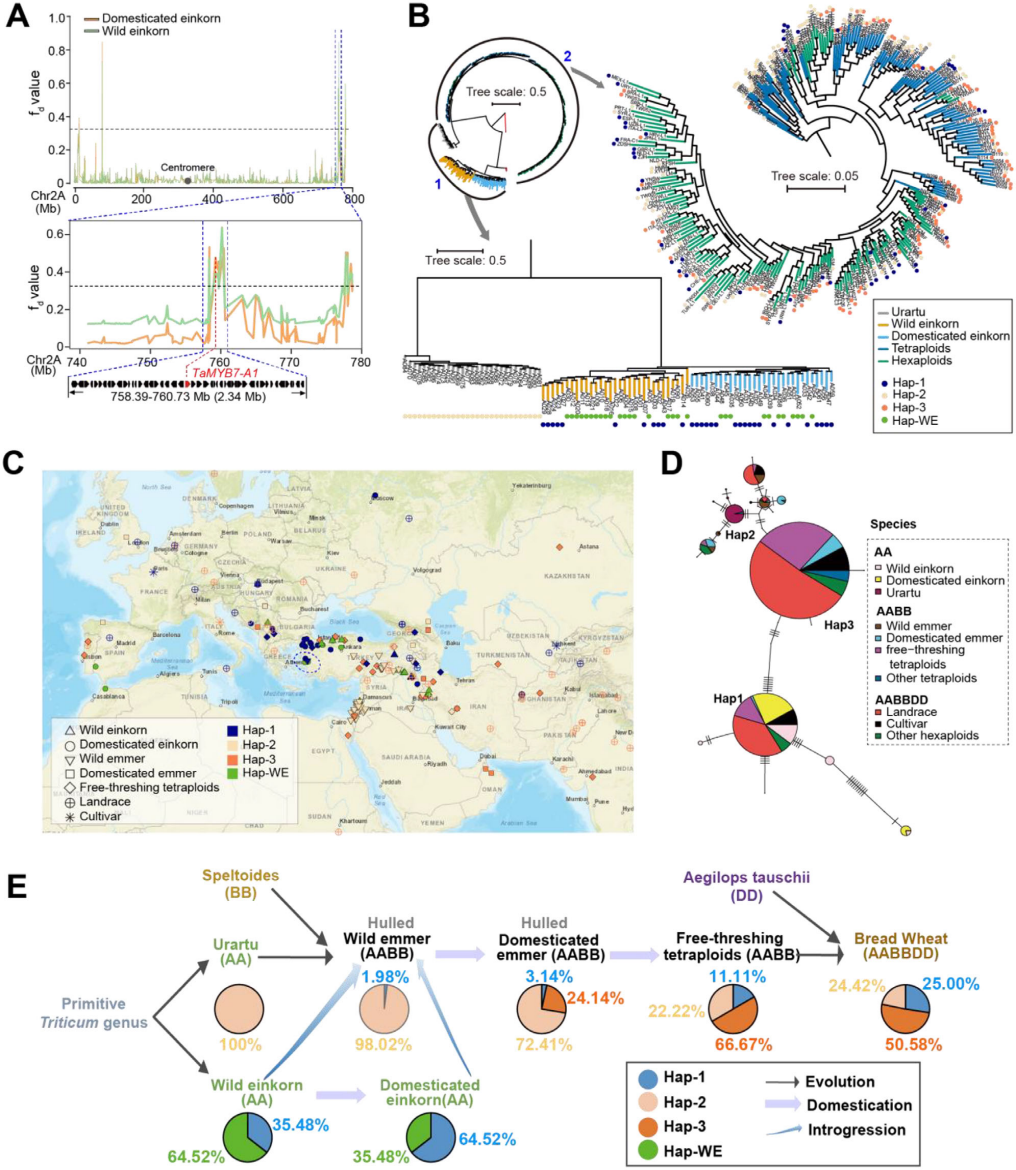
*TaMYB7‐A1^Hap−1^* originates from wild einkorn. (A) Introgression signals on chromosome 2A (top) and the genomic region flanking *TaMYB7‐A1* (bottom) from wild and domesticated einkorn. Introgression signals from wild einkorn and domesticated einkorn to common wheat are indicated by green and orange lines, respectively. Horizontal vertical lines represent the whole‐genome cutoff for defining the introgression signals. (B) Phylogeny of the wheat A‐genome lineage showing the allele distribution of *TaMYB7‐A1* in diploid, tetraploid and hexaploid wheat. Two clusters in the phylogenetic tree was featured in part 1 and part 2, respectively. The *TaMYB7‐A1* haplotypes of each accession were shown in different tip colors. Branch colors represent ploidy levels. Tip colors: blue, Hap‐1; yellow, Hap‐2; orange, Hap‐3; green, Hap‐WE (Hap‐WE was detected in einkorn but not in hexaploid wheat). Branch colors: gray, Urartu; orange, wild einkorn; blue, domesticated einkorn; dark blue, tetraploids; green, hexaploid wheat. (C) Geographical distribution of *TaMYB7‐A1* haplotype in bread wheat and its progenitors. Shapes denote species; colors represent haplotypes. Dashed circles highlight regions where *TaMYB7‐A1^Hap−1^* may have introgressed into hexaploid wheat. (D) Haplotype network of TaMYB7‐A1 in wheat and related species. Each circle represents a haplotype, with area proportional to its frequency. Lines between haplotypes indicate single‐nucleotide changes; small ticks on lines denote the number of mutations. (E) *TaMYB7‐A1* allele‐frequency changes during wheat evolutionary and domestication. Lightblue arrows indicate hypothesized introgression events.

To trace the origin of the superior *TaMYB7‐A1^Hap−1^* allele, we analyzed resequencing data from a global wheat diversity panel [31], encompassing Urartu (*T. urartu*, the AA genome donor of polyploid wheat), wild einkorn (*T. boeoticum*), domesticated einkorn (*T. monococcum*), wild emmer (*T. dicoccoides*), and hexaploid bread wheat (*T. aestivum*). Our analysis detected introgression fragments from wild einkorn on chromosome 2AL, precisely coinciding with the GWAS signals for *qPHS.2A.2* (Figure 5A).

Haplotype surveys confirmed that Hap‐1 occurs exclusively in einkorn, present in 11 of 31 wild and 19 of 30 domesticated accessions, but completely absent in all 29 Urartu accessions (Figure 5B; Table S12). Given its distinct and conserved haplotype structure, together with unique promoter variants, we propose that Hap‐1 has an exotic (non‐urartu) origin, likely introduced via introgression. This closer affinity to einkorn was further supported by sequence comparisons (Figure S22). Using kompetitive allele specific PCR (KASP) markers and Sanger sequencing, we identified Hap‐1 in 2 of 71 wild emmer accessions (Table S12), indicating its introgression into tetraploid wheat. An additional resequencing dataset [32] revealed a wild einkorn accession (*TA10585*) with high sequence similarity to the Hap‐1 allele in hexaploid wheat (Figure S23). Geographical distribution analyses suggest point to a likely origin in Serbia or northwestern Turkey, where domesticated einkorn may have served as the donor for Hap‐1 introgression into wild emmer (Figure 5C).

By contrast, Hap‐2 was fixed in all Urartu accessions, confirming its origin from the AA genome donor. This haplotype, along with the derived Hap‐3, was subsequently transmitted into tetraploid and hexaploid wheat. Hap‐3 itself arose within domesticated emmer through key nucleotide changes (G56A, G274T) that cause the amino acid substitutions Gly23Ser and Gly92Cys (Figure 5D and E). Another haplotype, Hap‐WE, remained exclusive to einkorn and was not incorporated into the polyploid wheat gene pool (Figure 5B‐E).

Haplotype network analyses delineates two distinct evolutionary trajectories: (i) Hap‐1 introgressed from einkorn into wild emmer and was retained in hexaploid wheat with minimal subsequent variation, and (ii) Hap‐2, inherited directly from Urartu, diversified into Hap‐3 during emmer domestication before both were maintained in modern cultivars. Phylogenetic evidence indicates domesticated einkorn as the most probable source of the Hap‐1 allele (Figure 5E).

Collectively, these findings reveal a complex evolutionary history for *TaMYB7‐A1*, shaped both by ancient introgression from wild einkorn and by natural selection acting on standing variation derived from Urartu during wheat domestication and diversification.

### 2.6 Precipitation‐Associated Diversity of TaMYB7‐A1 and Other PHS‐Resistance Loci

As wheat spread globally, diverse climatic factors such as temperature, photoperiod, and precipitation shaped its adaptive genetic variation [33]. *TaMYB7‐A1* shows a strong signature of introgression (Figure S24) and a notable association with seasonal precipitation (Figure S25). Its haplotype frequencies shifted dynamically along major dispersal routes: Hap‐1 increased into humid Southern Europe (westward), decreased in arid Southwest Asia, and rose again in moist East Asia (eastward) (Figure S25A). The geographic distribution of Hap‐1 displayed a moderate but significant correlation with annual precipitation (*R* = 0.57, *P* = 0.035; Figure S25B and Table S13), suggesting rainfall‐associated selection.

**FIGURE 6:**
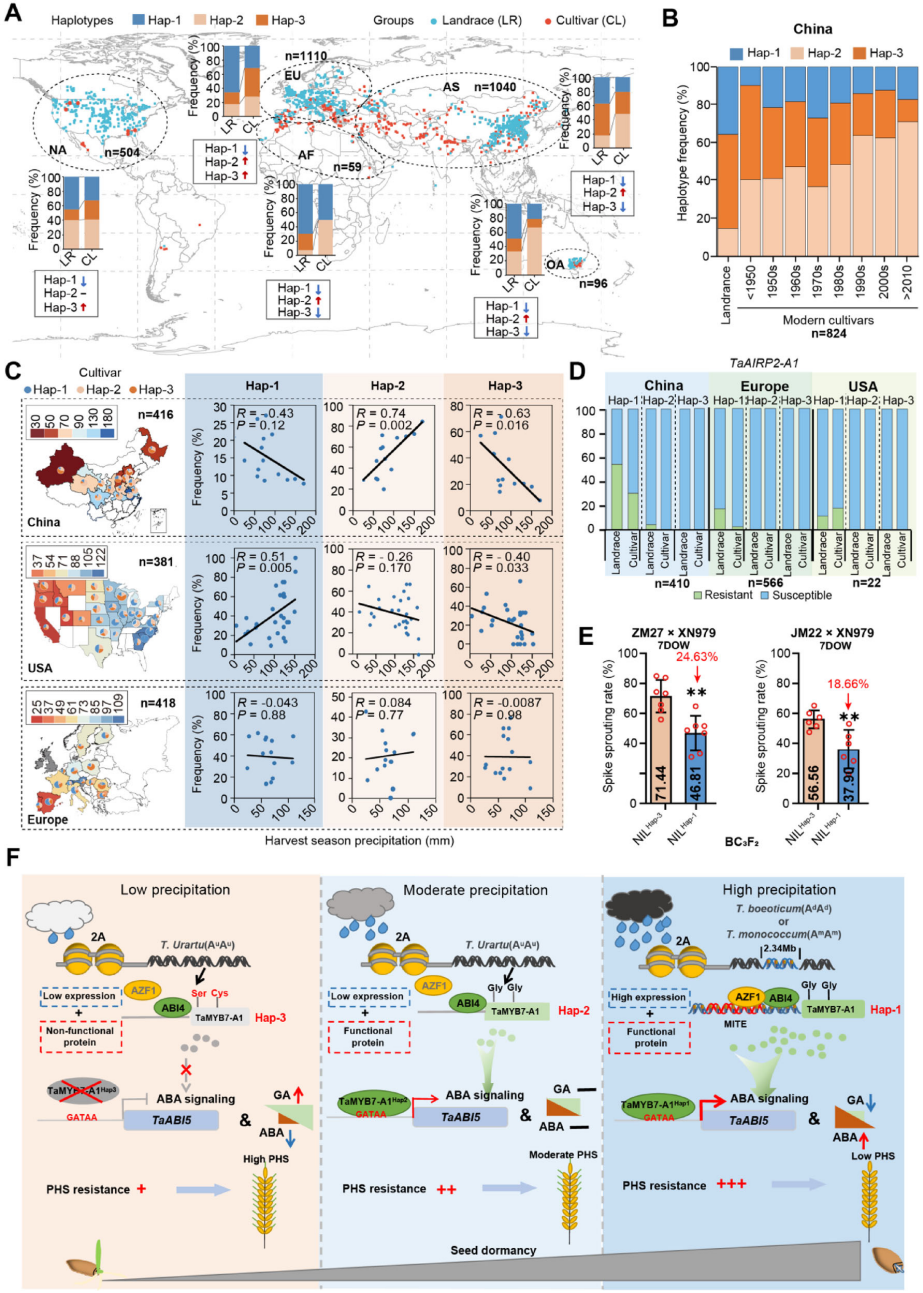
Global distribution of *TaMYB7‐A1* alleles correlates with seasonal precipitation. (A) *TaMYB7‐A1* allele frequency in landraces (blue dots) and cultivars (red dots) across the world's major wheat‐producing regions. Stacked bars show haplotype frequencies within each region (LR: Landrace; CL: Culitivar; AS: Asia; EU: Europe; NA: North America; AF: Africa; OA: Oceania). A novel GG insertion found in some Hap‐1 accessions lies outside conserved domains and does not alter haplotype classification. (B) Temporal changes in *TaMYB7‐A1* allele frequency during wheat breeding in China. (C) Geographical distribution of *TaMYB7‐A1* alleles and their correlation with local precipitation harvest season precipitation in China, Europe and USA. Harvest season precipitation is color‐coded from blue (high) to red (low); white indicates regions without data or outside major wheat areas. Pie‐chart size reflects sample number per region. Scatter plots show the correlation between haplotype frequency and precipitation across sub‐regions; trend lines were fitted using a general‐linear model. (D) Frequency of *TaAIRP2‐A1* resistant (green) and susceptible (blue) alleles among accessions carrying each *TaMYB7‐A1* haplotype in China, Europe and USA. (E) NILs were generated by introgressing the TaMYB7‐A1 locus from donor parent XN928 (Hap‐1) into the recurrent parents JM22 and ZM27 (both Hap‐3). Spike sprouting rate at 7 days of wetting (DOW) (n = 6 or 7 spikes), for NIL^Hap−3^ (in orange) and NIL^Hap−1^ (in blue). Data represent mean ± S.D. Germination performance of spike sprouting rate in NIL^Hap−1^ and NIL^Hap−3^ lines after 7 days of wetting (DOW). Significance was evaluated by Student's t‐test. ^**^, *P* < 0.01. (E) Spike sprouting rate after 7 days of wetting (DOW) for near‐isogenic lines (NILs) carrying Hap‐1 (blue) or Hap‐3 (orange) in the genetic backgrounds of JM22 and ZM27. Data are mean ± S.D. (*n* = 6–7 spikes). ^**^, *P* < 0.01 (Student's *t*‐test). (F) Summary model illustrating how combinatorial promoter (MITE) and coding‐sequence variations generate a gradient of PHS resistance. The einkorn‐introgressed Hap‐1 allele (high expression, functional protein) confers strong resistance in high‐rainfall regions (e.g., Europe); Hap‐2 (lower expression, functional protein) provides moderate resistance suited to multi‐cropping systems (e.g., Northern China); and Hap‐3 (low expression, impaired protein) confers susceptibility and persists in drier areas (e.g., Eastern USA). TaMYB7‐A1 activates ABA signaling via TaABI5 and fine‐tunes hormone homeostasis, enabling local adaptation of dormancy to regional precipitation regimes.

To explore this relationship, we genotyped *TaMYB7‐A1* across 3,132 accessions (Table S14), including 1,160 landraces, 1,842 cultivars, and 130 breeding or semi‐wild lines, using re‐sequencing data [34, 35, 36, 37, 38, 39] and KASP markers (Figure S26). Compared to landraces, modern cultivars generally carry lower Hap‐1 frequencies (Figure 6A), reflecting selection for rapid, uniform germination during domestication [8]. Regionally, Hap‐2 predominated in Asia, Australia, and northern Africa, whereas Hap‐3 is more common in Europe and North America (Figure 6A). In China, Hap‐1 has remained rare since the 1950s, with breeding gradually shifted from weak‐resistance Hap‐3 to moderate‐resistance Hap‐2 (Figure 6B).

Correlations between *TaMYB7‐A1* haplotypes and harvest‐season precipitation reveal distinct regional patterns. In China and the USA, weak‐resistance Hap‐3 is negatively correlated with precipitation, whereas moderate‐resistance Hap‐2 (China) and strong‐resistance Hap‐1 (USA) showed positive correlations (Figure 6C; Figure S27 and Table S15). No such association was detected in Europe, likely due to reliance on alternative PHS‐resistance loci. Notably, this precipitation correlation is unique among environmental variables such as altitude, temperature, and light intensity (Figure S28 and Table S16), underscoring the specific role of harvest season rainfall in shaping *TaMYB7‐A1* diversity.

Haplotype analyses of *TaMYB7‐A1* together with *TaAIRP2‐A1* and *TaMYB10‐D1* revealed additive effects on germination, with accessions carrying multiple resistant alleles showing stronger PHS resistance (Figure S29). Co‐selection patterns vary regionally: the resistant *TaAIRP2‐A1^R^* allele often co‐occurred with *TaMYB7‐A1^Hap−1^* in landraces across continents but persisted mainly in China and the USA cultivars (Figure 6D). In contrast, *TaMYB10‐D1^R^* has become the dominant resistance source in European cultivars (Figures S30 and S31), indicating that different genetic routes have been selected for regional adaptation to precipitation.

### 2.7 Breeding Potential of *TaMYB7‐A1* ^Hap−1^

Unlike other known PHS‐resistance loci, whose deployment in breeding has often been constrained by pleiotropic effects on grain color (*TaDFR* and *TaMYB10‐D1*) or yield (*TaVP1*), *TaMYB7‐A1^Hap−1^* confers robust PHS resistance without these associated trade‐offs. To evaluate its breeding value, we introgressed *TaMYB7‐A1^Hap−1^* from the resistant cultivar Xinong979 (XN979) into the susceptible cultivar Zhoumai 27 (ZM27) and Jimai22 (JM22). The resulting near‐isogenic lines, which share ∼90% genetic identity with their recurrent parents, exhibited significantly enhanced PHS resistance while maintaining normal growth and grain yield (Figure 6E; Figure S32). This unique combination of efficacy and agronomic neutrality positions *TaMYB7‐A1^Hap−1^* as an ideal genetic resource for developing PHS‐resistant, high‐yielding wheat cultivars, offering a powerful resource and a targeted breeding solution to mitigate rainfall‐induced pre‐harvest losses under increasingly variable climates.

## 3 Discussion

Wheat geographic adaptation is a complex process shaped by multiple interacting traits, including flowering time, vernalization requirements, and PHS‐resistance, which collectively determine the ecological fitness of cultivars across diverse environments [40, 41]. The rapid onset of climate change has altered precipitation patterns, increasing the risk of PHS events and posing a growing threat to global wheat production [42]. Here, we identify TaMYB7‐A1 as a central regulator of seed dormancy and demonstrate how natural variation at this locus has been shaped by evolution to fine‐tune PHS resistance across diverse precipitation regimes. Our study moves beyond gene discovery to provide a comprehensive case study—from causal variants and molecular mechanisms to evolutionary origin and environmental adaptation—offering a framework for understanding how genetic variation underpins climate resilience in polyploid crops.

### 3.1 Integration of Cis‐Regulatory and Coding Variation Generates Adaptive Phenotypic Gradients

Allele variations in both protein‐coding and regulatory regions, generating functional diversity [43]. A key finding of our work is that the phenotypic differences among *TaMYB7‐A1* haplotypes arise from the concerted effects of both promoter and coding‐sequence variation. We show that a haplotype‐specific MITE insertion acts as a *cis*‐regulatory enhancer, elevating *TaMYB7‐A1* expression by increasing chromatin accessibility and recruiting transcription factors such as TaAZF1. In parallel, non‐synonymous polymorphisms, particularly Gly23Ser and Gly92Cys, directly alter the protein's DNA‐binding affinity and transactivation capacity. This integration of *cis*‐ and *trans*‐variation creates a functional continuum: Hap‐1 (strong enhancer, functional protein) confers high resistance suited to high‐rainfall regions (Western Europe and parts of North America [44, 45]); Hap‐2 (weak enhancer, functional protein) provides moderate resistance optimal for China's intensive double‐cropping systems where rapid post‐harvest germination is advantageous [46]; and Hap‐3 (weak enhancer, non‐functional protein) confers susceptibility and persists in drier areas. This mechanism exemplifies how combinatorial variation at a single locus can generate finely tuned phenotypic gradients that mirror environmental selection pressures, in this case, harvest‐season precipitation.

### 3.2 TaMYB7‐A1 Enhances Dormancy Through a Defined Genetic Hierarchy within the ABA Pathway

Our study delineates a clear genetic module through which TaMYB7‐A1 exerts its effect. We provide multiple lines of genetic evidence, including knock‐out/OE phenotyping, ChIP‐qPCR, and complementation assays with downstream targets, that firmly establish TaMYB7‐A1 enhances PHS resistance by directly activating *TaABI5*, a core component of ABA signaling [47]. This places TaMYB7‐A1 upstream in a regulatory cascade. Furthermore, we confirm that *TaMYB7‐A1* itself is activated by the transcription factor TaABI4, evidenced by promoter binding, transactivation assays, and reduced expression in *Taabi4* mutants. This TaABI4‐TaMYB7‐A1‐TaABI5 module defines a coherent transcriptional pathway that amplifies ABA signaling to enforce dormancy. While additional genetic evidence to further solidify this hierarchy is being pursued, our focus here is on how natural variation modulates this pre‐existing regulatory module. This approach reveals the evolutionary mechanism by which a complex agronomic trait is fine‐tuned in a polyploid crop.

### 3.3 Adaptive Introgression from Wild Relatives as a Source of Elite Alleles

Phylogenetic and haplotype analyses reveal that the superior *TaMYB7‐A1^Hap−1^* allele was introgressed into the wheat gene pool from wild einkorn (*T. boeoticum*) or domesticated einkorn (*Triticum monococcum*), not inherited from its direct A‐genome progenitor *T. urartu* [31]. This finding underscores the importance of adaptive introgression in crop evolution. The associated MITE enhancer likely arose in the einkorn lineage prior to introgression, suggesting that wild relatives can contribute not only novel coding sequences but also potent regulatory innovations. While deletion of the MITE in stable transgenic lines through CRISPR‐Cas9 would provide a more solid causal link, this work remains ongoing due to technical challenges inherent to wheat.

Introgression from wild relatives has long provided valuable genetic resources for wheat improvement [31]. Alleles such as *Yr34*, *Sr21*, and *Sr22b* introduced from einkorn and other wild species have conferred durable resistance to stripe and stem rust [48, 49, 50]. This study adds PHS resistance to this list, demonstrating a viable evolutionary route to recover adaptive variation that may have been narrowed during domestication bottlenecks. Unlike many domestication‐derived alleles that incur yield or developmental penalties [10, 12, 14], *TaMYB7‐A1^Hap−1^* enhances resilience without compromising agronomic performance. The persistence and geographic correlation of Hap‐1 with high rainfall regions highlight its role in local adaptation.

In addition to PHS resistance, our recent work indicates that TaMYB7‐A1 also contributes to drought resilience and water‐use efficiency [51], likely through modulating root architecture and reactive oxygen species homeostasis. This dual functionality, conferring resistance to both pre‐harvest sprouting and drought stress, highlights its value as a versatile genetic resource for adapting to climate instability, particularly under unpredictable rainfall patterns. As climate change continues to destabilize rainfall patterns [46], utilizing such pleiotropic wild‐derived alleles will be crucial for sustaining crop productivity and quality in stress‐prone environments [33].

### 3.4 Breeding Implications: A Superior Allele for Climate Resilience

A major constraint in deploying PHS‐resistance genes has been the pleiotropic effects on other agronomic traits. TaVP‐1 regulates seed dormancy through ABA signaling but negatively affect embryo development and plant vigor [13]. TaMYB10‐D1, while enhancing dormancy via activation of *TaNCED* and increased ABA synthesis, confers red grain pigmentation, an undesirable trait for noodles and steamed bread in East Asia [14]. Similarly, alleles at *TaAIRP2‐A1* contribute to PHS resistance but are associated with trade‐offs in grain yield.

By contrast, *TaMYB7‐A1^Hap−1^* confers strong PHS resistance without detectable pleiotropic penalties. The development of near‐isogenic lines confirms its efficacy in enhancing PHS resistance while maintaining performance. The development of KASP markers specific to *TaMYB7‐A1* haplotypes now enables precise selection of favourable alleles in breeding populations (Figure S26). Pyramiding of *TaMYB7‐A1^Hap−1^* with other complementary alleles (e.g., *TaMYB10‐D1^R^* in Europe, *TaAIRP2‐A1^R^* in China/USA) enables of tailor dormancy strength to regional climates. This provides breeders with a versatile and “clean” genetic tool to mitigate harvest losses under increasingly unpredictable rainfall, embodying a strategy to harness naturally selected, wild‐derived variation for climate‐smart agriculture.

## 4 Methods

### 4.1 Plant Materials

The genome‐wide association study (GWAS) included 187 wheat accessions, predominantly winter and semi‐winter types, with a small number of spring wheat accessions, all exhibiting similar maturity periods to the winter wheat varieties. Complete passport data for all accessions, including geographical origins and growth habits, are provided in Table S1. This deliberate selection minimized variation in heading and maturation timing, improving the reliability and precision of post‐maturation dormancy assessments. All accessions were evaluated at the experimental field of the Institute of Genetics and Developmental Biology, Chinese Academy of Sciences, Beijing (39.91°N, 116.39°E), over three growing seasons: 2017–2018 (E1), 2018–2019 (E2), and 2020–2021 (E3). A completely randomized design with two replicates per accession was employed. Rows were 1.5 m in length, with 25 cm row spacing and 10 cm plant spacing. Agronomic management followed local cultivation practices, and representative main spikes from inner rows were harvested for phenotyping of PHS‐resistance, agronomic, and yield traits.

Transgenic lines and introgression lines were multiplied at Zhangjiakou, China (40.82°N, 114.88°E) and planted in the experimental field of the Institute of Genetics and Developmental Biology, Chinese Academy of Sciences. The *TaABI5‐A1* OE lines were cultivated in the greenhouse of the Peking University Institute of Advanced Agricultural Sciences (36.50°N, 119.42°E) under controlled wheat growth conditions (22°C, 70% humidity).

### 4.2 Bacterial Strains

Escherichia *coli* DH5α was cultured in LB medium at 37°C for plasmid DNA extraction. *E. coli* BL21 (DE3) was cultured in LB medium at 37°C for recombinant protein expression. *Agrobacterium tumefaciens* GV3101 was cultured in LB medium at 28°C for *N. benthamiana* transient expression. *Agrobacterium tumefaciens* EHA105 was cultured in LB medium at 28°C for transgenic plant transformation.

### 4.3 Evaluation of Pre‐Harvest Sprouting (PHS) Resistance

For the seed germination assay, fully developed, healthy, and morphologically uniform seeds at 35 days after pollination (DAP) were selected and dried at 37°C for 6 h to reduce moisture content below 10%. Threshed seeds were surface‐sterilized in 1% (m/v) NaClO for 10 min, followed by thorough rinsing with sterile distilled water to remove residual disinfectant. Seeds were plated on filter paper moistened with distilled water (fully saturated but without free water) and incubated in complete darkness at 25°C ± 1°C in temperature‐controlled chambers to eliminate environmental variation. Germination was strictly defined as the simultaneous emergence of both the radicle (> 2 mm) and the coleoptile (visible shoot tip). Daily monitoring was performed under aseptic conditions, moldy seeds were removed, and the remaining seeds were rinsed. Final germination percentage was determined after 7 days of incubation. Three independent biological replicates were conducted, each with ∼50 seeds, and results were reported as mean germination rates ± standard deviation.

For the spike sprouting assay, intact spikes were harvested at physiological maturity (35 DAP) and dried at 37°C for 6 h to reduce moisture content below 10%, standardizing baseline conditions. Spikes were fully immersed in distilled water for 6 h to achieve uniform hydration, then affixed vertically and densely to boards simulating natural field conditions. They were incubated at 25°C under >80% relative humidity, maintained with plastic covers and supplemental misting three times daily. To reduce positional bias, spike placement was initially randomized, and positions were rotated regularly throughout the incubation period. Germination was defined by the concurrent emergence of both radicle (> 2 mm) and coleoptile (longer than half the glume). Germination rate was calculated as the proportion of germinated grains per spike, with measurements taken after 7 days of incubation. Three independent biological replicates were conducted, with more than four spikes per line, and data were reported as mean germination rates ± standard deviation.

For the ABA treatment assay, seeds harvested at 35 DAP were partially released from dormancy by storage at room temperature for 15 days. Prior to treatment, seeds were dried at 37°C for 6 h to standardize moisture content. Surface‐sterilized seeds were placed on filter paper moistened with 50 µM ABA solution or distilled water (control) and incubated at 25°C in complete darkness. Germination was assessed after 7 days using the same criteria as above. Three biological replicates were performed for each treatment, with ∼50 seeds per replicate.

### 4.4 Generation of Transgenic Lines

The coding sequences of *TaMYB7‐A1* and *TaAIRP2‐A1* from Chinese Spring were amplified or synthesized and cloned into the pLGY‐UBI vector to generate overexpression constructs using the Infusion HD Cloning Kit (SC612, Genes and Biotech Co., Ltd, China), with gene expression driven by the *Ubiquitin* promoter. For targeted knock‐out of *TaMYB7‐A1*, two single‐guide RNAs (sgRNA1, 5′‐AGACGCCCGAGGAGGTCCGGCGG‐3′; sgRNA2, 5′‐AAGATAGCCGACGCCGTCGAGGG‐3′) were designed to target homologous conserved, genome‐specific sequences within the first exon.

Constructs were transformed into calli derived from immature embryos of *cv*. Fielder to generate transgenic plants. Identification and expression analyses were performed using primers listed in Table S17.

### 4.5 Genome‐Wide Association Studies (GWAS)

The 187 accessions GWAS panel was genotyped using Affymetrix Wheat660K SNP arrays (Capital Bio Corporation, Beijing, China). After filtering for minor‐allele frequency ≤ 5%, missing rate ≥ 10%, and hybrid rate ≤ 5%, 277358 high‐quality SNPs uniquely mapped to the IWGSC RefSeq v1.0 reference genome were retained for analysis. Seed germination rates (SSR) from each environment, as well as best‐linear‐unbiased prediction (BLUP) values across environments, were used for GWAS in Tassel v5.2 employing a mixed linear model. Manhattan and quantile–quantile (Q–Q) plots were generated using R package “CMplot” (https://github.com/YinLiLin/CMplot), while pairwise linkage‐disequilibrium (LD) values (*r^2^*) were calculated and visualized in Haploview 4.2. The number of LD blocks (N) was estimated using Plink 1.9 (https://www.cog‐genomics.org/plink/), and a *P*‐value of 3.7E‐04 (*P* = 1/N) was adopted as the suggestive whole‐genome significance threshold according to the adjusted Bonferroni method. SNPs significantly associated in the BLUP dataset and at least two individual environments were defined as stable QTLs (*P*‐value < 3.7E‐04).

### 4.6 RNA‐seq and RT–qPCR

Developing grains at 15, 25, and 35 DAP from Fielder, overexpression lines (OE1 and OE2), and knockout lines (KO1 and KO2) were collected in three independent biological replicates. Total RNA was extracted using the Quick RNA isolation Kit (HuaYueYang, ZH120). First‐strand cDNA was synthesized from 2 µg total RNA using a reverse‐transcription kit (Vazyme, R211‐01) according to the manufacturer's instructions.

Fielder and KO1 were conducted to RNA‐seq analysis. RNA‐seq libraries were prepared using standard protocols and sequenced on the BGISEQ‐500 platform at BGI Technology. Raw reads were filtered using fastp v0.20.1 [52] to remove low‐quality reads and adapters. Clean reads were aligned to the IWGSC RefSeq v1.0 genome using HISAT2. Gene‐level raw read counts were calculated and normalized to transcripts per million (TPM) [53]. Differentially expression genes (DEGs) were identified using DESeq 2 [54] with a threshold of adjusted p‐value (*P.adj*) < 0.05 and |log_2_Foldchange| > 0.5. Gene ontology (GO) enrichment analysis was performed using http://geneontology.org/ and visualized with ggplot2 (https://ggplot2.tidyverse.org/). Transcriptome data used for candidate‐gene screening were obtained from WheatOmics (http://202.194.139.32/), and raw data were filtered to retain genes with TPM ≥ 1. Binding‐motif information for transcription factors was obtained from PlantTFDB (https://planttfdb.gao‐lab.org/).

Fielder, KO1, KO2, OE1, and OE2 were conducted to RT‐qPCR validation. Real‐time quantitative PCR (RT–qPCR) was performed using a QuantStudio5 instrument (Applied Biosystems) with SYBR Green Master Mix (Vazyme, Q111‐02), with three biological replicates per sample. Relative expression was calculated via the 2^−ΔΔCT^ method, with *TaTubulin* (*TraesCS1D01G353100*) as the internal control. Gene‐specific primers are listed in Table S17.

Developing (15, 20, 25, and 35 DAP) and imbibed (1, 3, 6, 12, 24, 36, 60, 84, 106, and 156 h after imbibition) seeds of Chinese Spring (CS) were used for expression profiling of candidate genes. Methods and controls same as described above. Gene‐specific primers are listed in Table S17.

### 4.7 Assay for Transposase‐Accessible Chromatin with RT–qPCR (ATAC–qPCR)

We performed ATAC–qPCR on 15 DAP embryos following established protocols [55]. Input was used for normalization, and *TaTubulin* (*TraesCS1D01G353100*) served as the control. Primers used for ATAC–qPCR are listed in Table S17.

### 4.8 Sanger Sequencing of TaMYB7‐A1

To precisely characterize natural variation within TaMYB7‐A1, we performed Sanger sequencing of its genic region (including ∼2.5 kb promoter and the entire coding sequence) in 115 wheat accessions. This set comprised 41 accessions selected from the original GWAS panel to retain its population structure and the full spectrum of pre‑harvest sprouting (PHS) variation, plus 74 randomly chosen accessions from the Chinese wheat mini‑core collection to maximize germplasm representation and allelic diversity. Primers were designed to amplify overlapping fragments covering the target region; primer sequences are listed in Table S17. PCR products were purified and sequenced by BGI Genomics (Shenzhen, China) using the Sanger method. Sequences were assembled, aligned, and manually curated to define haplotypes and polymorphisms.

### 4.9 Yeast Two‐Hybrid Assay

Coding sequences of *TaABI4* and *TaAZF1* were amplified from cDNA derived from 15 DAP grains and cloned into prey (pGADT7) and bait (pGBKT7) vectors. Yeast two‐hybrid assays were performed using the Frozen‐EZ Yeast Transformation II kit (Zymo, T2001) according to the manufacturer's instructions, with combinations of TaABI4–AD, TaABI4–BD, TaAZF1–AD, and TaAZF1–BD constructs. Transformed Y2HGold yeast strains were selected on double dropout (‐Trp/‐Leu) and quadruple dropout (‐Trp/‐Leu/‐His/‐Ade) media. Primers sequences are provided in Table S17.

### 4.10 Chromatin Immunoprecipitation (ChIP) qPCR

Chromatin immunoprecipitation was performed as described previously with some modifications [56]. To investigate the in vivo binding of TaMYB7‑A1 to the promoters of its downstream target genes, ChIP assays were performed using developing seeds (15 days after pollination) collected from Fielder and homozygous *pUbi*::TaMYB7‑A1‑His transgenic bread wheat lines. Chromatin was cross‑linked with 1% formaldehyde, extracted, and sonicated to an average fragment size of 200–500 bp.

ChIP with non‐specific rabbit IgG and anti‐His antibody for WT and OE lines. After reverse cross‐linking, the immunoprecipitated DNA was purified and subjected to RT‐PCR using SYBR Green Master Mix on a LightCycler 96 Real‐Time PCR system. Primers were designed to amplify fragments containing the predicted TaMYB7‐A1 binding sites in the promoters of four selected target genes (primer sequences are listed in Table S17).

Enrichment of each target fragment was calculated using the percent input method. The fold enrichment relative to the IgG control was then calculated by dividing the % Input of the specific antibody (anti‐IgG and anti‐His) by the % Input of the corresponding IgG control. Data are presented as the mean ± SD of three independent biological replicates. Individual biological replicates are shown as dots in the figures.

### 4.11 Dual‐Reporter Assays

∼2.5‐Kb promoter regions of *TaABI5‐A1*, *TaABI5‐B1*, *TaABI5‐D1*, and *TaPYL9‐D1* were amplified from Chinese Spring genomic DNA, while ∼2.5‐Kb promoter regions of *TaMYB7‐A1* were either mutated from Chinese Spring or amplified from varieties carrying different haplotypes. These fragments were inserted into the CP461‐LUC vector to generate reporter constructs. Coding sequences of *TaMYB7‐A1* (from different haplotype), mutated *TaMYB7‐A1*, *TaABI4*, and *TaAZF1* from Chinese Spring were cloned into the PTF101 vector as effectors.

For the transformation in *N. benthamiana* leaves, effector and reporter plasmids were transformed into *A. tumefaciens* GV3101 and co‐infiltrated into *N. benthamiana* leaves at the 6–8‐leaf stage. At 2–3 days post‐infiltration, firefly luciferase (LUC) and Renilla luciferase (REN) activities were measured using a Dual‐luciferase Assay System (Promega, E1910). Chemiluminescence was quantified with a microplate reader (SpectraMax iD3, Molecular Devices), and relative LUC activity was calculated as the LUC/REN ratio. Primers used for vector construction are listed in Table S17.

For the transformation in wheat protoplasts, effector and reporter plasmids were transfected into wheat protoplasts. The protoplasts were isolated from the etiolated shoots of 7–10‐day‐old Chinese Spring seedlings by enzymatic digestion. After transfection, the protoplasts were incubated in the dark at 23°C for 16–20 h. The luminescence activities of firefly luciferase (LUC) and Renilla luciferase (REN) were then measured using a Dual‐Luciferase Assay System (Promega, E1910), and the relative LUC activity was calculated as the ratio of LUC to REN. The primers used for plasmid construction are listed in Table S17.

### 4.12 Bimolecular Fluorescence‐Complementation (BiFC) Assay

Coding sequences of *ABI4* and *AZF1* were amplified from cDNA prepared from RNA isolated from 15 DAP Chinese Spring grains and cloned into the SCYNE R and SCYCE R vectors to generate ABI4–NE, ABI4–CE, AZF1–N, and AZF1–CE constructs. Relevant vector pairs were co‐injected into *N. benthamiana* leaves using *A. tumefaciens* GV3101 (OD600 = 0.5). After injection, the plants were maintained in a greenhouse for 48 h. Fluorescence signals were then detected by confocal laser‐scanning microscopy (Leica TCS SP5). Primers used are listed in Table S17.

### 4.13 Electrophoretic Mobility Shift Assay (EMSA)

EMSA was performed as previously described [57] with modifications to reduce non‐specific binding. Recombinant TaMYB7‐A1^Hap−1^, TaMYB7‐A1^Hap−2^, TaMYB7‐A1^Hap−3^ proteins and their respective mutants, fused to MBP tags, were expressed in *E. coli* BL21 and purified using MBP beads following induction with 0.5 mM IPTG at 28°C for 16 h.

For the binding reaction, competitor or mutant probes were pre‐incubated with purified protein at 25°C for 10 min before addition of the biotin‐labeled probe (5′‐biotin, BGI; sequences in Table S17), followed by 20 min incubation. Protein‐DNA complexes were separated and transferred to a nylon membrane, and signals were detected using the Chemiluminescent EMSA Kit (Thermo Scientific, 20148) according to the manufacturer's instructions.

### 4.14 Quantification of Endogenous ABA and GAs from Plant Tissues

Approximately 100 mg fresh weight (FW) of plant tissue was ground in liquid nitrogen, weighed, and extracted for 24 h with methanol containing ^2^H_6_‐ABA as an internal standard. Endogenous ABA was purified and quantified as described previously [58] with modifications to detection conditions. LC‐MS/MS was performed on a UPLC system (Waters) coupled to a 5500 Qtrap system (AB SCIEX). LC separation used a BEH C18 column (1.7 µm, 100 × 2.1 mm; Waters) with mobile phase A, 0.05% (v/v) acetic acid in water, and B, 0.05% (v/v) acetic acid in acetonitrile. The gradient was set with an initial 20% B and increased to 70% B within 6 min. ABA was detected in multiple reaction monitoring (MRM) mode with a transition. The MRM transitions for ABA and ^2^H_6_‐ABA were 263.0 > 153.1 and 269.2 > 159.2.

Endogenous GAs were extracted and quantified as previously described [58]. Briefly, 100 mg FW tissues were homogenized in liquid nitrogen and extracted with 90% methanol (MeOH) containing ^2^H_2_‐GAs as internal standards. Extracts were centrifuged at 20 000 *g* for 15 min at 4°C, and the supernatant was subjected to purification on coupled MCX–WAX cartridges (3cc, 60 mg) (Waters). After washing with 90% MeOH, the WAX cartridge was separated, rinsed with 5% formic acid, and eluted with MeOH. Samples were dried and re‐dissolved in 40% MeOH for UPLC–MS/MS analysis. Detections were performed on a UPLC instrument (Waters) coupled to a 6500 Qtrap MS system (AB SCIEX) equipped with an electrospray ionization (ESI) source. The UPLC methods and MS parameters were set as reported previously [58]. The MRM transitions for quantification of GAs were as follows: GA_12_, 331.2 > 313.2; ^2^H_2_‐GA_12_, 333.2 > 315.2; GA_19_, 361.2 > 273.2; ^2^H_2_‐GA_19_, 363.2 > 275.2; GA_34_, 347.2 > 259.2; ^2^H_2_‐GA_34_, 349.2 > 261.2; GA_53_, 347.2 > 303.2 and ^2^H_2_‐GA_53_, 349.2 >305.2.

Hormone assay service was supported by the Plant Hormone Analysis Platform of the Institute of Genetics and Developmental Biology, Chinese Academy of Sciences.

### 4.15 Pyrosequencing Analysis of DNA Methylation (BS‐seq)

DNA methylation was analyzed by CENCEFE Biotechnology Co., Ltd (Jiangsu, China) using quantitative pyrosequencing. Genomic DNA was extracted from plant tissues using the DNeasy Plant Mini Kit (Qiagen, 69104) following the manufacturer's protocols. Bisulfite conversion of 500 ng genomic DNA was performed with the EpiTect Bisulfite Kit (Qiagen, 59104) using the following thermal profile: 95°C for 5 min; 60°C for 25 min; 95°C for 5 min; 60°C for 85 min; 95°C for 5 min; and 60°C for 175 min. PCR amplification was carried out with specifically designed biotinylated primers (Table S17) under the following parameters: 95°C for 3 min; 40 cycles of 94°C for 30 sec, 50°C for 30 sec, and 72°C for 1 min; followed by a final extension at 72°C for 7 min. Nested PCR was applied when necessary to improve amplification specificity. Pyrosequencing was performed on a PyroMark Q48 Autoprep system (Qiagen) using the PyroMark Q48 Advanced CpG Reagents (Qiagen). In brief, 10 µL of biotinylated PCR product was immobilized on streptavidin‐coated beads and processed following the manufacturer's workflow. Methylation at individual CpG sites was quantified with PyroMark Q48 Software v2.5.8, applying quality‐control thresholds of >95% bisulfite conversion efficiency and >90% sequence similarity to the reference. All data were obtained from Sanger sequencing of amplicons with subsequent methylation analysis.

### 4.16 TaMYB7‐A1 Haplotype Analysis

A total of 3,132 wheat accessions were analyzed to assess the distribution and breeding dynamics of *TaMYB7‐A1* haplotypes, including 1,160 landraces, 1,842 varieties, 58 breeding lines, and 72 Tibetan semi‐wild accessions (Table S14). Of these, haplotype for 1,878 accessions were identified using whole‐genome re‐sequencing data (http://wheat.cau.edu.cn/WheatUnion/), and 1,254 accessions were genotyped using KASP markers. Detailed polymorphism and haplotype information are provided in Table S14, with KASP marker sequences listed in Table S17.

A haplotype network was constructed based on 36 SNPs and one InDel within the *TaMYB7‐A1* coding sequence, using a previously reported *Triticum* population [31]. Missing and heterozygous variants were imputed with Beagle v5.4 [59], and haplotype construction was performed using the R package pegas v1.3. The haploNet function was used to define the number of haplotypes and mutation steps among linked haplotypes.

### 4.17 Phylogenetic Analysis

*TaMYB7‐A1* sequences and homologous proteins from wheat (*Triticum aestivum*), Urartu (*T. urartu*), *Aegilops tauschii*, barley (*Hordeum vulgare*), sorghum (*Sorghum bicolor*), maize (*Zea mays*), rice (*Oryza sativa*), and *Arabidopsis thaliana* were retrieved from PlantTFDB. Protein sequences were aligned using ClustalW, and a phylogenetic tree was constructed using the neighbor‐joining method in MEGA 11 with default parameters. Phylogeny testing was computed using the bootstrap method with 10,000 replications, and evolutionary distances were calculated using a Poisson model.

To specifically resolve the A‐genome lineage of *TaMYB7‐A1*, 200,000 randomly selected SNPs were used to infer a phylogeny with RAxML v8.2.12 under the GTRGAMMA model, with 100 bootstrap replicates. The resulting tree was visualized in the iTOL [60].

### 4.18 Detection of Introgression from Einkorn

A four‐taxon *f_d_* statistic [61] was used to identify genomic segments introgressed from wild einkorn and domesticated einkorn wheat to common wheat, using barley as an outgroup. The *f_d_* values and Tajima's *D* statistic across the A subgenome were estimated using Python code (available at https://github.com/simonhmartin/genomics_general), with a sliding window size of 100 SNPs and step size of 5 SNPs. *F_d_*‐statistic values for windows with *D* < 0 were converted to 0, as negative *D*‐statistic values are meaningless. The top 5% sliding windows of each population were considered as introgression genomic regions. P1, Indian dwarf wheat; P2, Landraces; P3, wild einkorn or domesticated einkorn wheat; O, Barley.

### 4.19 Detection of Environmental‐Association Signals

Eight precipitation‐ and 11 temperature‐related bioclimatic factors were extracted from WorldClim (http://www.worldclim.org/) and their association with genomic variants was determined using BayPass v2.1. The EXTRACT function of the R package RASTER v.3.3.13 was used to extract climate data for geographical coordinates according to wheat accessions. Population structure was estimated using 30,000 randomly selected SNPs under the BayPass core mode, resulting 13 populations. Bayes factor was calculated under the standard covariate model to evaluate the association of SNP frequencies of populations with environmental variables, and the median of five independent Baypass runs was used. Pseudo‐observed data with random 10,000 SNPs were used for the core model, and a 1% observed Bayes factor was considered as the threshold for identification of statistically significant association signals.

### 4.20 Selective‐Sweep Analysis

The cross‐population composite likelihood ratio (XP‐CLR) v1.0 was used to detect selective sweeps during the dispersal of landraces by calculating pairwise selective sweeps between wheat groups (West Asia, Europe, Inner Asia, East Asia, and Southern Himalaya) using parameters of ‐w1 0.005 500 10000 ‐p1 0.95. The genetic distance was estimated using previously reported recombination‐rate data. The R package GenWin was used to normalize XP‐CLR statistics and detect the boundary of genomic regions with smoothness = 2,000 and method = 4. The top 5% statistic results from each population were considered as selective sweeps.

### 4.21 Construction of Near‐Isogenic Lines (NIL) for TaMYB7‐A1

The PHS‐resistant cultivar Xinong979 (XN979) carrying the *TaMYB7‐A1^Hap−1^* allele was crossed with PHS‐susceptible cultivar Zhoumai27 (ZM27) and Jimai22 (JM22) as recurrent parents, the resulting F_1_ plants were backcrossed three times with ZM27 and JM22 to generate the BC_3_F_1_ population. In each successive generation, *TaMYB7‐A1* was genotyped using KASP markers (Table S17), and heterozygous hybrids were used for backcrossing. Heterozygous BC_3_F_1_ plants were self‐pollinated, and the resulting BC_3_F_2_ progenies were used to score PHS‐resistance and agronomic traits.

### 4.22 Statistics and Data Visualization

R version 4.0.2 and GraphPad Prism 8.0.2 were used to compute statistics and generate plots, unless specified. The sample size (n) for each figure is indicated either in the figures and in the corresponding figure legends. Data in column charts are expressed as the mean ± standard error of the mean (SEM), unless otherwise specified. Data in box plots show median with min‐to‐max whiskers. A p‐value less than 0.05was considered statistically significant. For comparisons of two groups of data that fit a normal distribution, Student's t‐test was used to compare means (Figure 1G,H and 2C–G and 4D,F and 6E; Figures S6A–C and S8B–D and S9 and S11 and S12A–C and S13D,E and S14C,E and S15C–F and S16C and S19A,B and S20 and S32). For comparisons of two groups of data that do not fit a normal distribution, the Wilcoxon rank‐sum test was used (Figures 1D,E and 3B; Figures S5D,E). For three or more comparisons of independent groups of data, Tukey's HSD multiple‐comparisons test was used (Figures 3D,E,G,H and 4A,B,C,H,I; Figure S3 and S15B,G and S17A,B and S18 and S19C,D). Pearson's correlation coefficient was used in Figure 6C and Figures S1 and S14B and S21B and S25B and S28 and S31.

## Author Contributions

J.X., Z.‐X.T., and C.‐C.C. designed and supervised the research, H. W. did most of the experiments, Q.Z. provides the natural population with genotypes and some agronomic traits, M. Z. did two years phenotyping and GWAS analysis, H.W. and D.‐Z.W. did one year phenotyping and GWAS analysis; Y.‐F.G. did most of the introgression analysis; J.S., J.H., X.‐Y.Z., and C.U. did haplotype analysis and partial introgression analysis of UK wheat varieties; H.W. and D.‐Z.W. analyzed the distribution and correlation of *TaMYB7‐A1* haplotypes, and its combinations with *TaMYB10‐D1* and *TaAIRP2‐A1*; X.‐M. L. performed RNA‐seq analysis and identified potential targets of TaMYB7‐A1; X.‐M.B. and X.‐S.Z. generated multiple TaMYB7‐A1‐OE and genome editing plants; X.‐H.L. and Y.C. provided TaABI5‐OE plants and help with SSR analysis; D.‐Z.W., H.W., and J.X. prepared all the Figures; J.X., D.‐Z. W., and H.W. wrote the manuscript; X.‐L.L., F.L., Z.‐X.T., and C.‐C.C. polished the manuscript. All authors discussed the results and commented on the manuscript.

## Funding

This research was supported by the National Key Research and Development Program of China (2024YFE0115100, 2021YFD1201500), the National Natural Science Foundation of China (U22A6009, 32572353, 32388201, 32130095, 32225038), the Beijing Natural Science Foundation Outstanding Youth Project (JQ23026), the Strategic Priority Research Program of the Chinese Academy of Sciences (XDA24010204), and the National Key Research and Development Program of China (2022YFF1002904, 2023YFF1000604).

## Conflicts of Interest

The authors declare no conflicts of interest.

## Code Availability

Code used for RNA‐seq analysis is available in GitHub (https://github.com/WangHao‐BOOp/TaMYB7‐A1_PHS_resistance_RNA‐seq).

## Supporting information




**Supporting File 1**: advs75522‐sup‐0001‐SuppMat.docx.

**Supporting File 2**: advs75522‐sup‐0002‐Supplementary‐Table1‐18.xlsx.

## Data Availability

Sequence data generated in this study are found in the EMBL library (http://plants.ensembl.org/index.html) under the following accession numbers: TaAIRP2‐A1, TraesCS2A02G586800; TaMYB7‐A1, TraesCS2A02G554200. The raw‐sequence data of RNA‐seq in this study were deposited in the Genome Sequence Archive (https://bigd.big.ac.cn/gsa) under accession number CRA030125.
