# Supplementary material for: Introgressed Variation in TaMYB7‐A1 Drives Graded Dormancy and Climate‐Adaptive Pre‐Harvest Sprouting Resistance in Wheat
Source: Adv Sci (Weinh). 2026 May 7;13(42):e24067. doi: 10.1002/advs.202524067 (PMC13335674; doi:10.1002/advs.202524067)
Supplement: Supplementary file 1 — Supporting File 1: advs75522‐sup‐0001‐SuppMat.docx. [file ADVS-13-e24067-s001.docx]

**
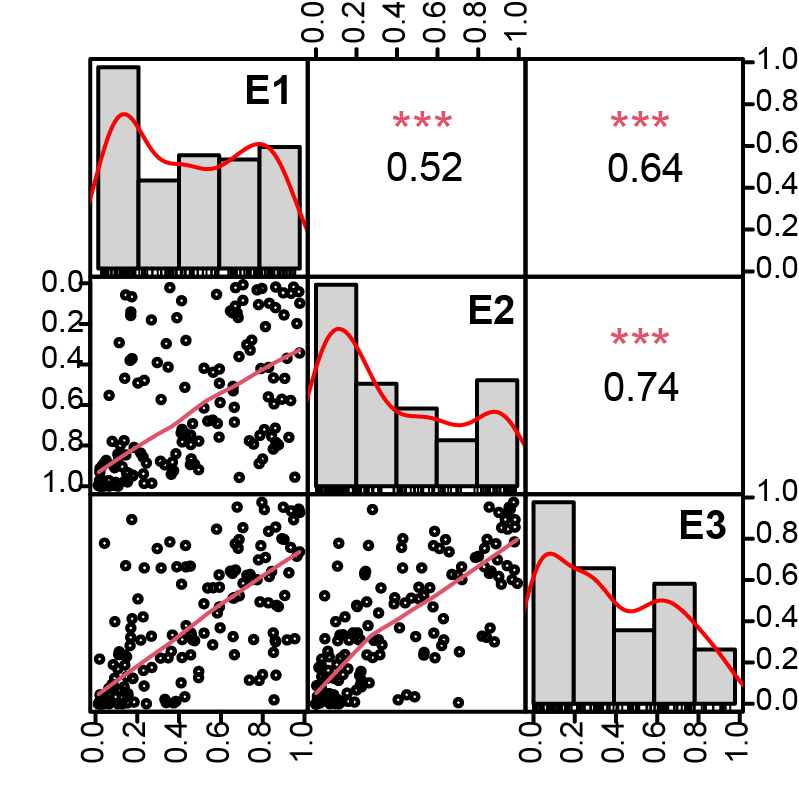
**

**Figure S1 Distribution and pairwise correlation of germination rate among environments**

The frequency distribution of germination rate at each environment (E1–3) is shown in the histogram at the diagonal cells. The X–Y scatter plot of correlations between environments is in the lower-triangle panel and the corresponding Pearson’s correlation coefficients between each trait are shown in the upper-triangle panel. ***, *P* < 0.001. Environments: E1, year 2018; E2, year 2019; E3, year 2021.


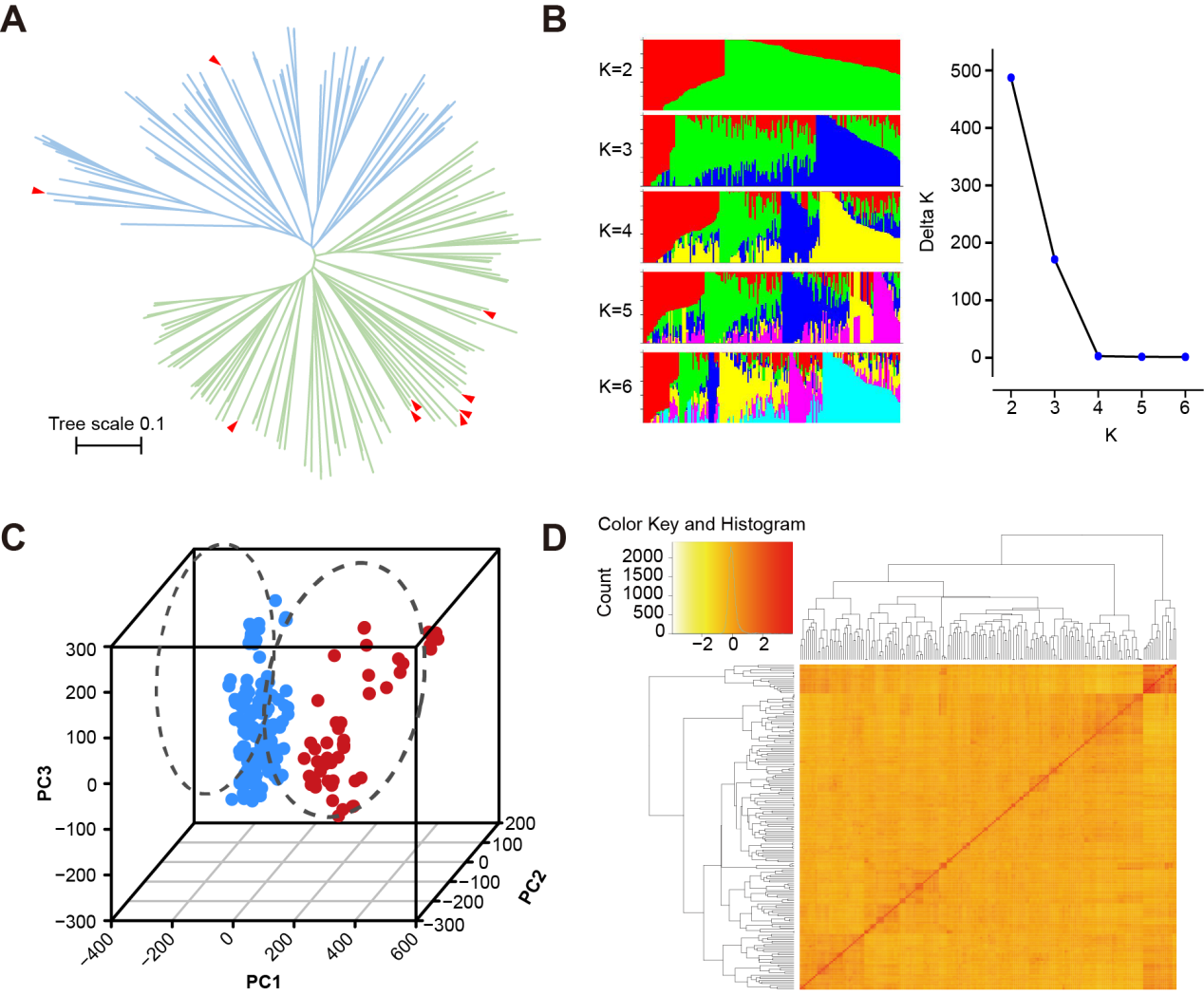


**Figure S2 Phylogenetic analysis and population structure of the GWAS panel**

(A) Phylogenetic relationships of 187 wheat accessions in the GWAS panel. The neighbor-joining tree delineates two subpopulations (blue and green branches), with spring wheat cultivars highlighted by red triangles.

(B) Histogram of the STRUCTURE analysis of GWAS panel for the model with K = 2 to K = 6. The max estimated ΔK values for the given sub-population number (K) was identified at K = 2.

(C) Principal-component analysis of the population structure for the 187 accessions in the GWAS panel. The population formed two subgroups depicted in blue and red dots.

(D) K-matrix clustering of genetic-relationship values among the 187 wheat accessions.


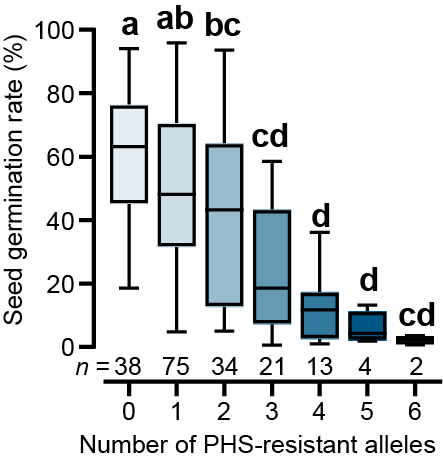


**Figure S3 Additive effects of eight PHS-resistance loci identified by GWAS**

Box plot demonstrates progressively enhanced PHS resistance (reduced germination rates) in accessions accumulating favorable haplotypes across these loci. BLUP value of seed germination rate in GWAS panel were used for analysis. The box denotes the 25^th^, median, and 75^th^ percentiles, and the whiskers indicate the 1.5× interquartile range. Different lowercase letters indicate significant differences (*P* < 0.05, Tukey's HSD test).


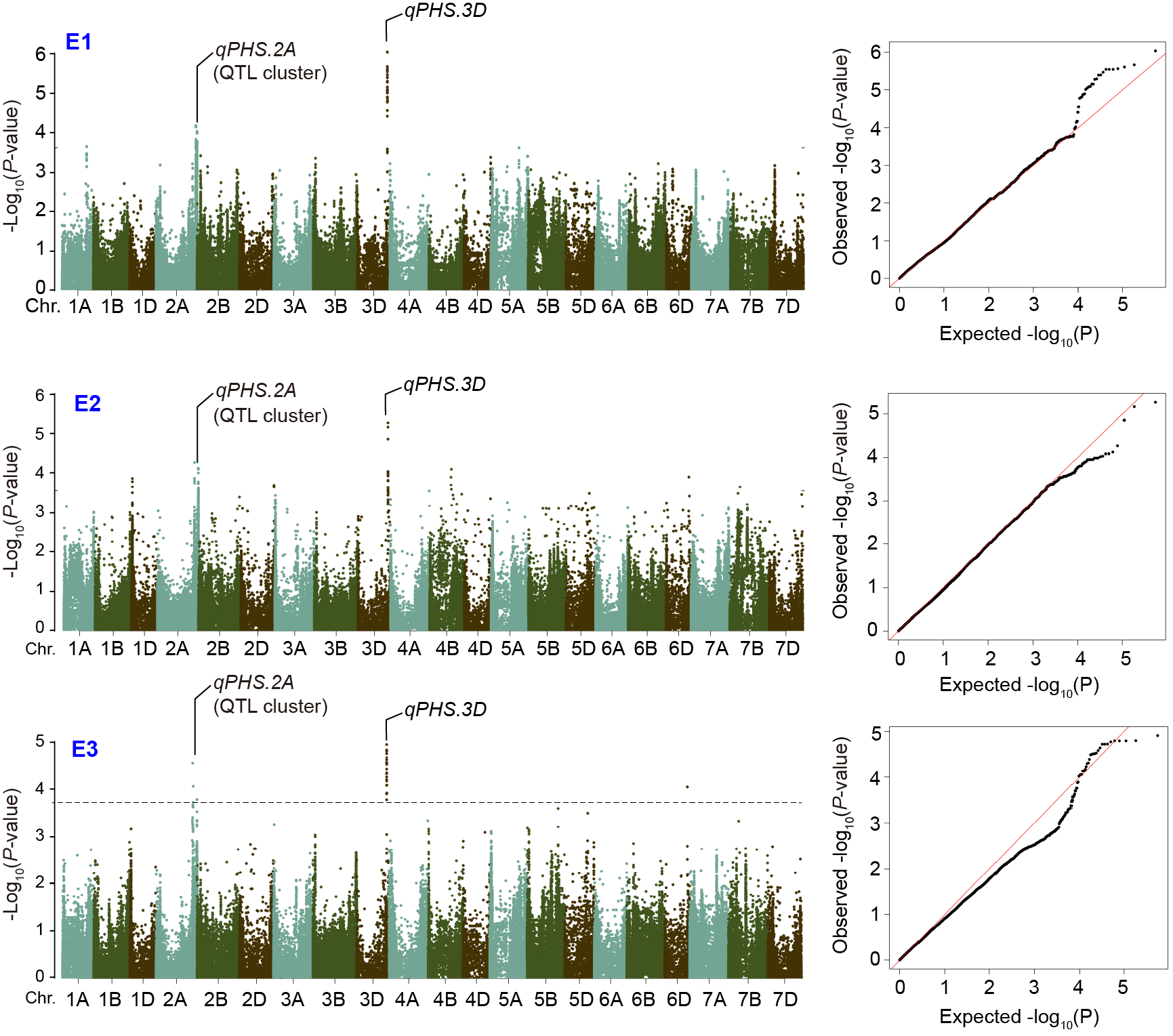


**Figure S4 Significant association signals for seed-germination rate across environments**

Manhattan plot and quantile-quantile plot were used for illustrating statistically significant association signals for seed-germination rate in the GWAS panel. y-axis, -Log_10_(*P*-value) of SNPs against their chromosomes location in the x-axis. The horizontal dashed line indicates the genome-wide suggestive statistical-significance threshold (*P* = 3.7E-04) for marker–trait associations.


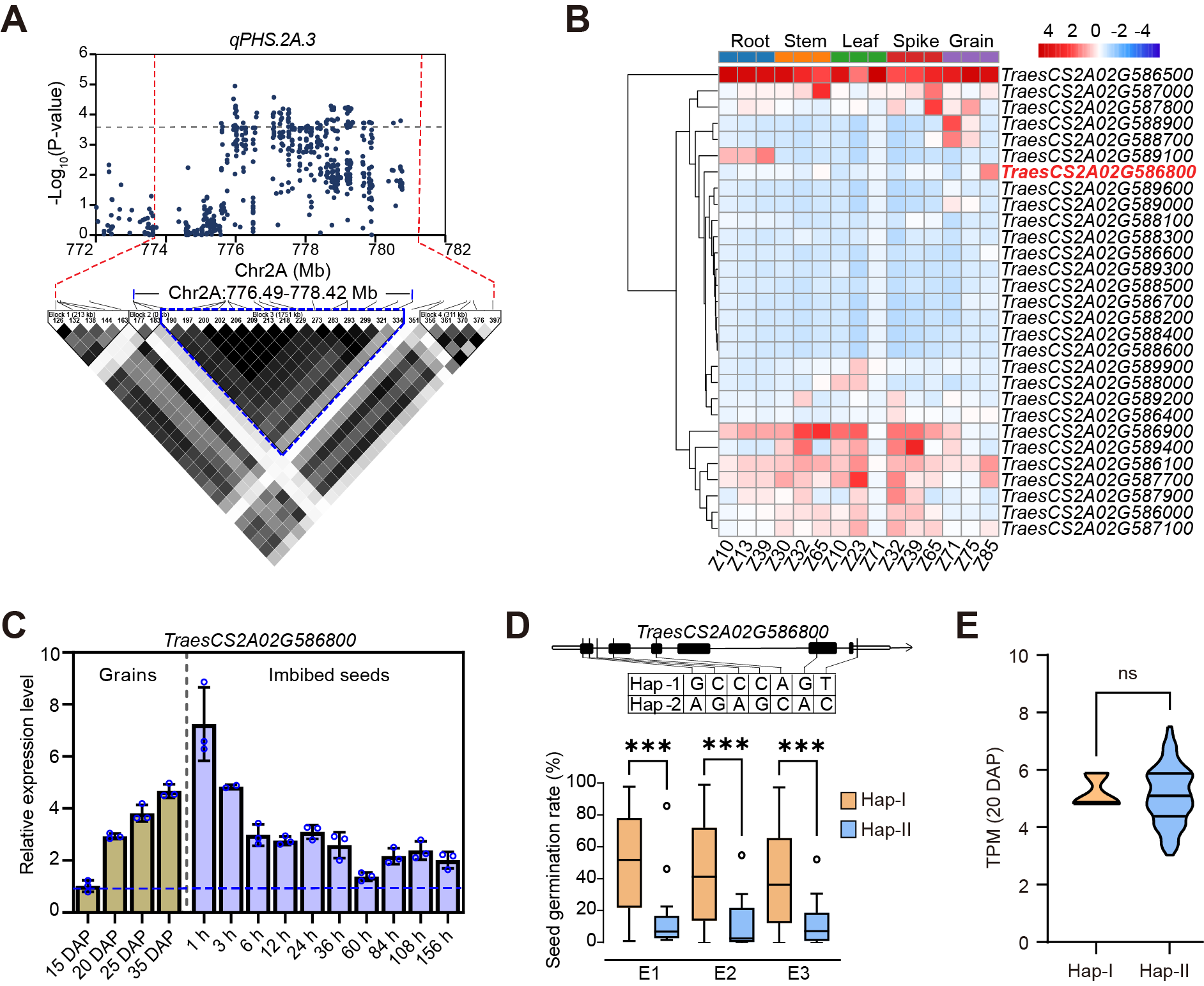

**Figure S5 *TaAIRP2-A1* is the candidate gene underlying *qPHS.2A.3***

(A) Local Manhattan plot (top) and linkage-disequilibrium heatmap (bottom) of *qPHS.2A.3* association peaks. The intensity of color from white to black represents the range of *r^2^* values from 0–1.

(B) Spatiotemporal expression analysis of 29 candidate genes located in the 1.98-Mb linkage-disequilibrium interval for *qPHS.2A.3*. A z-score normalization is performed on the expression levels across different tissues for each gene. *TaAIRP2-A1* (*TraesCS2A02G586800*) was marked in red.

(C) RT-qPCR of *TaAIRP2-A1* dynamics during grain development (brown bars) and seed imbibition (purple bars). Data are means ± S.D. from three independent biological replicates. *TaTubulin* was used as the internal control, and expression levels were normalized with grains at 15 DAP set to 1 (blue dashed line).

(D) Germination rates for *TaAIRP2-A1* haplotypes across environments. The box denotes the 25^th^, median, and 75^th^ percentiles, and the whiskers indicate the 1.5× interquartile range. ***, *P* < 0.001 (Wilcoxon rank‐sum test).

(E) Violin plot comparing *TaAIRP2-A1* expression in grains between two haplotypes determined by RNA-seq data at 20 DAP from 102 wheat varieties. ns, *P* ≥ 0.05 (Wilcoxon rank‐sum test).


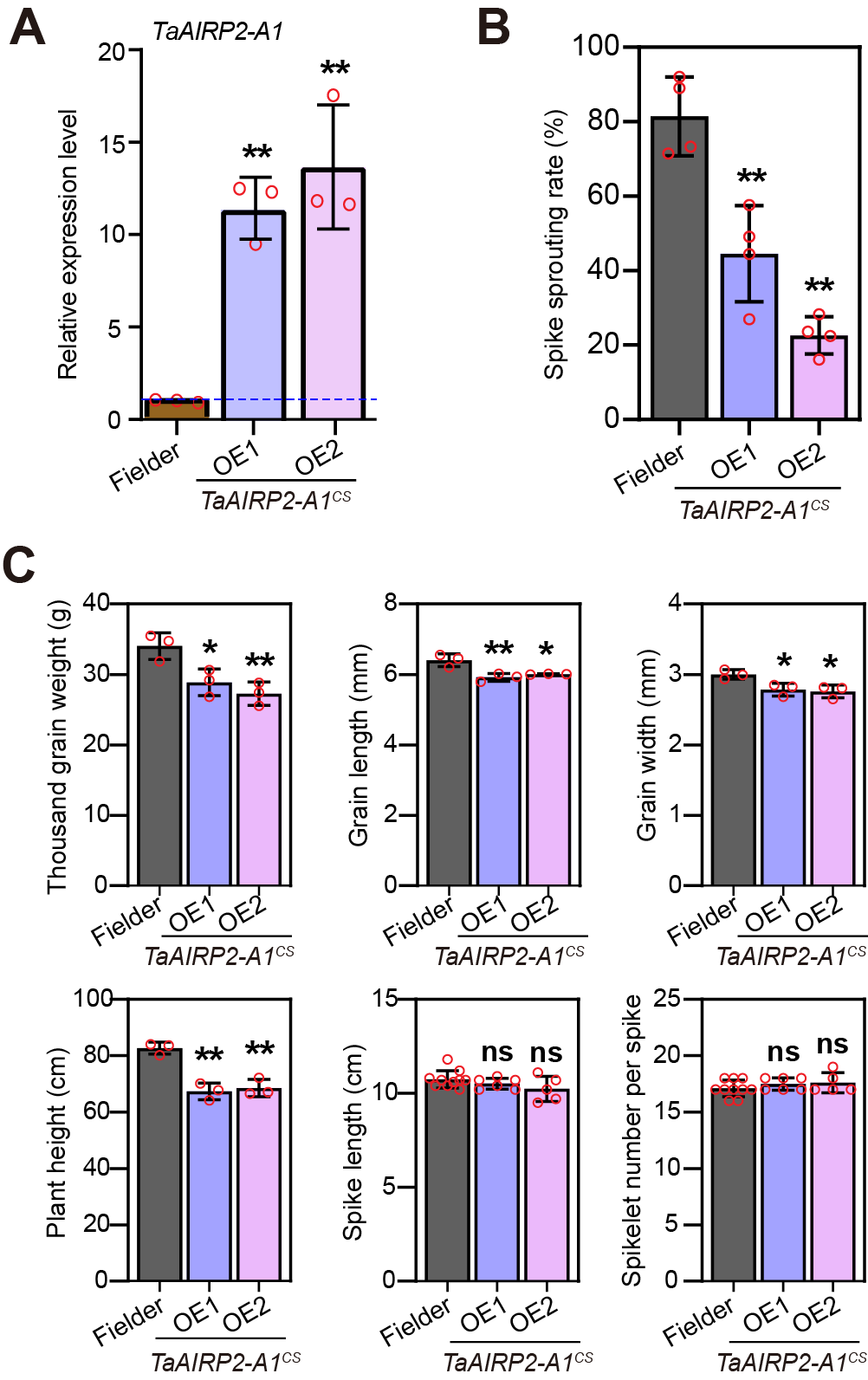


**Figure S6 *TaAIRP2-A1* enhances PHS-resistance but imposes growth penalties**

(A) RT-qPCR of *TaAIRP2-A1* in Fielder and *TaMYB7-A1* OE lines. *TaTubulin* was used as the internal control, and expression levels were normalized with Fielder set to 1 (blue dashed line). Data are the mean ± S.D. of three biological replicates. **, *P* < 0.01 (Student’s *t*-test).

(B) Spike sprouting rate at 7 days of wetting for Fielder and *TaAIRP2-A1* over-expression lines. Data represent means ± S.D. of three independent biological replicates. **, *P* < 0.01 (Student’s *t*-test).

(C) Agronomic traits for Fielder and *TaAIRP2-A1* over-expression lines. Data represent means ± S.D. from ≥ 3 individual plants per line. **, *P* < 0.01; *, *P* < 0.05; ns, *P* ≥ 0.05 (Student’s *t*-test).


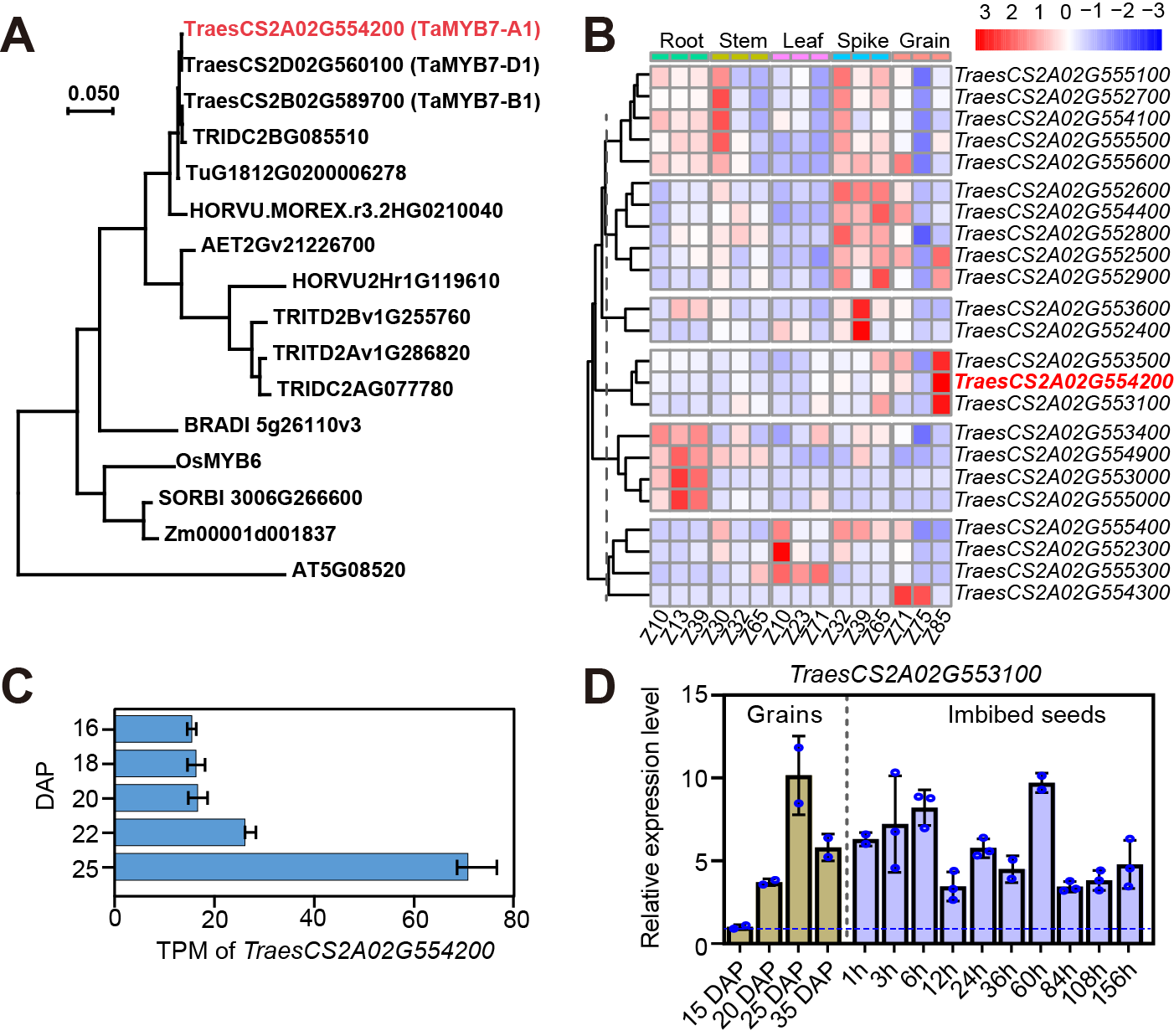


**Figure S7 *TaMYB7-A1* is the candidate gene underlying *qPHS.2A.2***

(A) Phylogenetic tree of TaMYB7-A1 and its homologous proteins. The homologous protein of TaMYB7-A1 in the *Grass* family were subjected for phylogenetic tree construction using a neighbor-joining algorithm by MEGA 4 after 1000 bootstrap. The homologous protein from *Arabidopsis thaliana* was used as an outgroup. The scale bar indicates the average number of amino acid substitutions per site.

(B) Heatmap of spatiotemporal expression analysis of 23 candidate genes located in the 2.02-Mb linkage disequilibrium interval for *qPHS.2A.2* in Chinese Spring. A z-score normalization is performed on the expression levels across samples for each gene. *TaMYB7-A1* was marked in red.

(C) Seed-specific expression profiles of *TaMYB7-A1* obtained from RNA-seq analysis of developing wheat seeds in Chinese Spring. The seed developmental stages are indicated by days after pollination (DAP).

(D) RT-qPCR of *TraesCS2A02G553100* dynamics during grain development (brown bars) and seed imbibition (purple bars). *TaTubulin* was used as the internal control, and expression levels were normalized with grains at 15 DAP set to 1 (blue dashed line). Data are represent mean ± S.D. of three biological replicates.


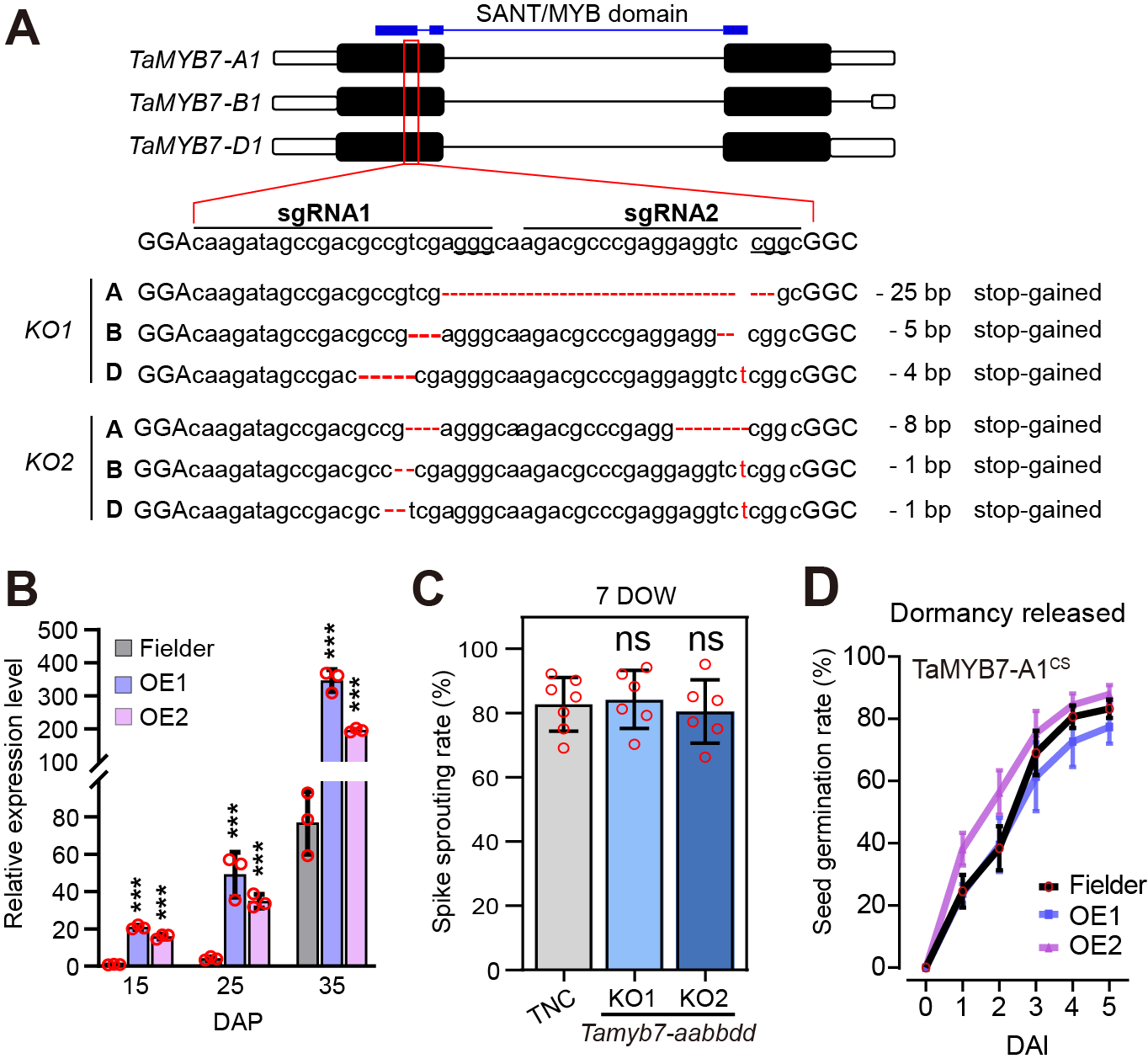


**Figure S8 *TaMYB7-A1* positively regulates wheat PHS-resistance**

(A) Schematic of *TaMYB7* homeolog genomic structures (top, with SMART predicted conserved SANT/MYB domain marked in blue) showing the target sites and PAMs for single guide RNAs used for mutagenesis by CRISPR-Cas9 in the Fielder background. Sequences of Fielder and two recovered mutant alleles (designated KO1 and KO2) are shown (bottom).

(B) RT-qPCR analysis of *TaMYB7-A1* expression in 15, 25, 35 days after pollination (DAP) grains from Fielder and *TaMYB7-A1^CS^* over-expression (OE) lines. Values represent means ± S.D. of three biological replicates. *TaTubulin* was used as the internal control, and expression levels were normalized with Fielder (15 DAP) set to 1. ***, *P* < 0.001 (Student’s *t*-test)..

(C) Spike sprouting rate (SSR) for TNC versus KO lines (7 DOW). Data represent mean ± S.D. from 5-9 spikes. **, *P* < 0.01; ns, *P* ≥ 0.05 (Student’s *t*-test).

(D) Germination time course for dormancy-released seeds from Fielder and *TaMYB7-A1* OE lines. Dissected grains were kept at room temperature for 60 days before used for analysis. Data represent mean ± S.D. of three biological replicates (with ~50 seeds for each replicate). No significant difference in means between Fielder and transgenic lines at each day after imbibition (DAI) as *P* ≥ 0.05 (Student’s *t*-test).


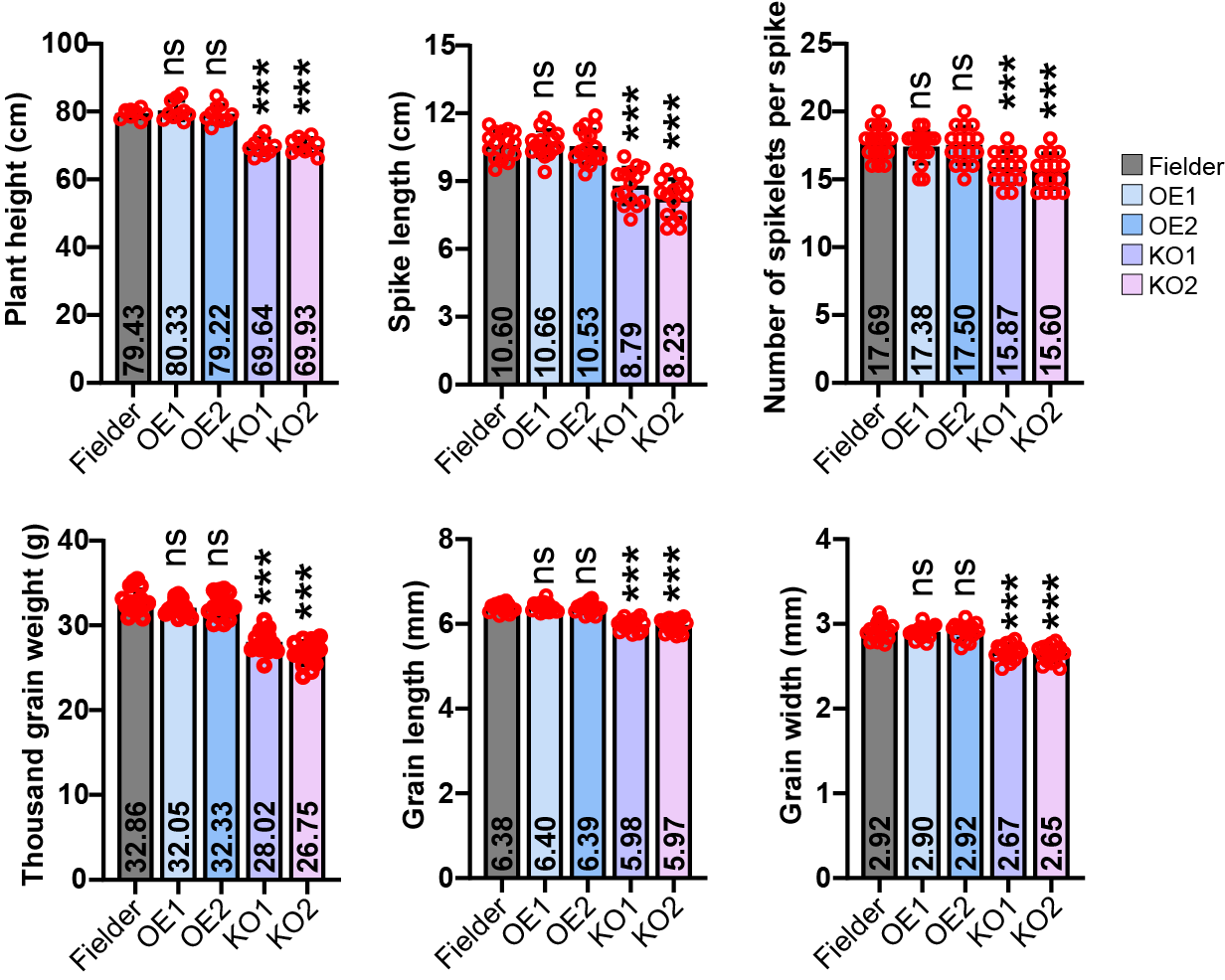


**Figure S9 Agronomic traits of *TaMYB7-A1* transgenic lines**

Data presented mean ± S.D.; each individual was considered as one biological replicate (*n* ≥ 15). *, *P* < 0.05; ***, *P* < 0.001; ns, *P* ≥ 0.05 (Student’s *t*-test).


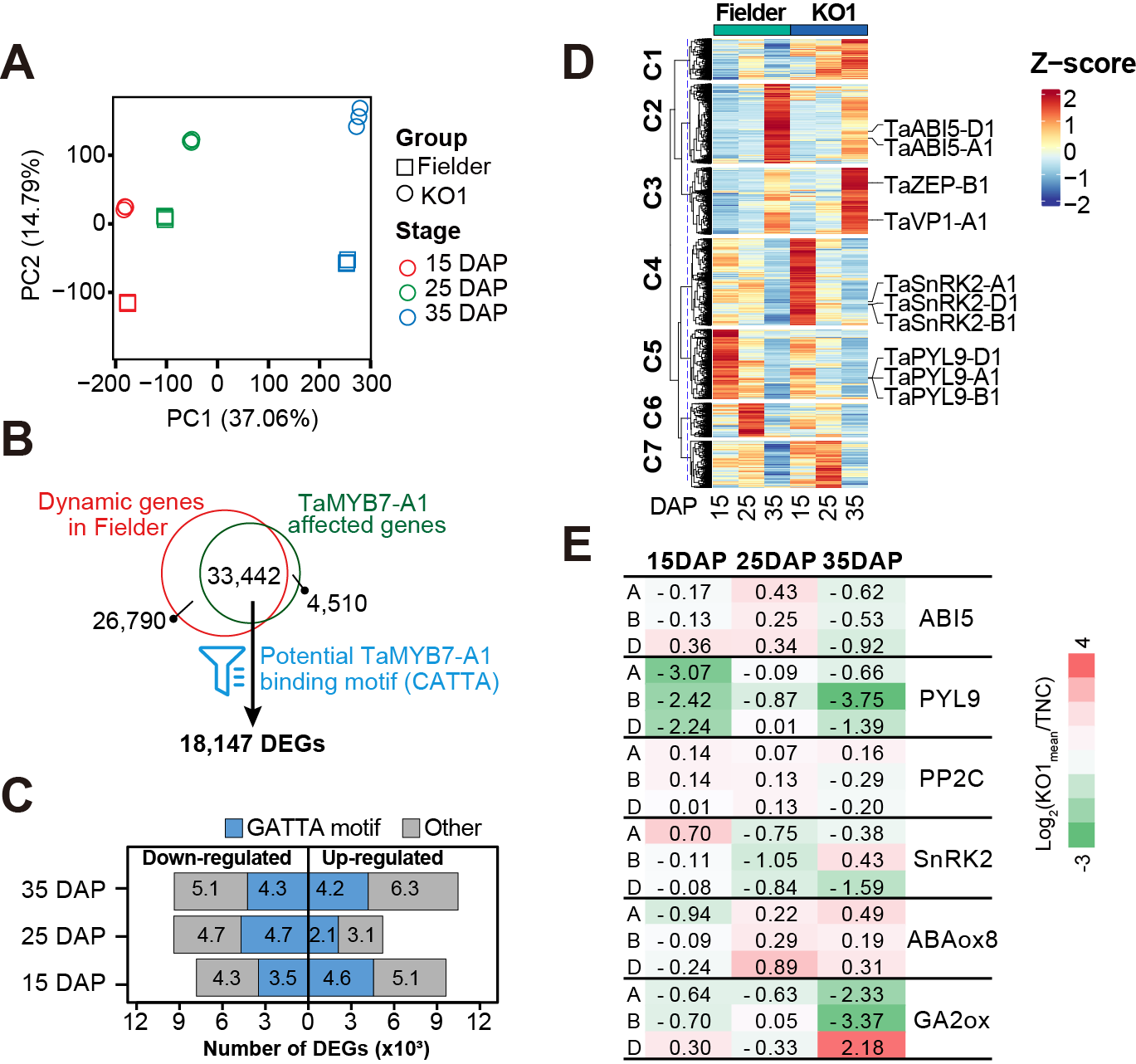


**Figure S10 RNA-seq overview and downstream genes regulated by TaMYB7-A1**

(A) Principal-component analysis of transcriptome from developing grains at 15, 25 and 35 days after pollination (DAP). Fielder and *TaMYB7* knockout line KO1 are represented by dots in different shape. Three biological replicates were sequenced each genotype.

(B) Venn diagram showing overlapping DEGs identified using three analytical approaches: temporal DEGs across seed developmental stages in Fielder (red), genotype-specific DEGs between KO1 and Fielder (green), and potential downstream targets of TaMYB7-A1 filtered by presence of motifs in accessible proximal promoter regions (cyan text).

(C) RNA-seq quantification of up- and down-regulated DEGs in 15, 25, and 35 DAP grains between *TaMYB7* KO line and Fielder. The number of DEGs with and without GATAA-binding motifs in their promoter region were shaded in blue and gray, respectively.

(D) Hierarchical clustering of differentially expressed genes (DEGs) between Fielder and knockout line KO1. Genes associated with the ABA signaling pathway are listed.

(E) Heatmap showing the average log_2_(fold change) value of ABA/GA related individual subgenome homologous genes in KO1 compared to Fielder.


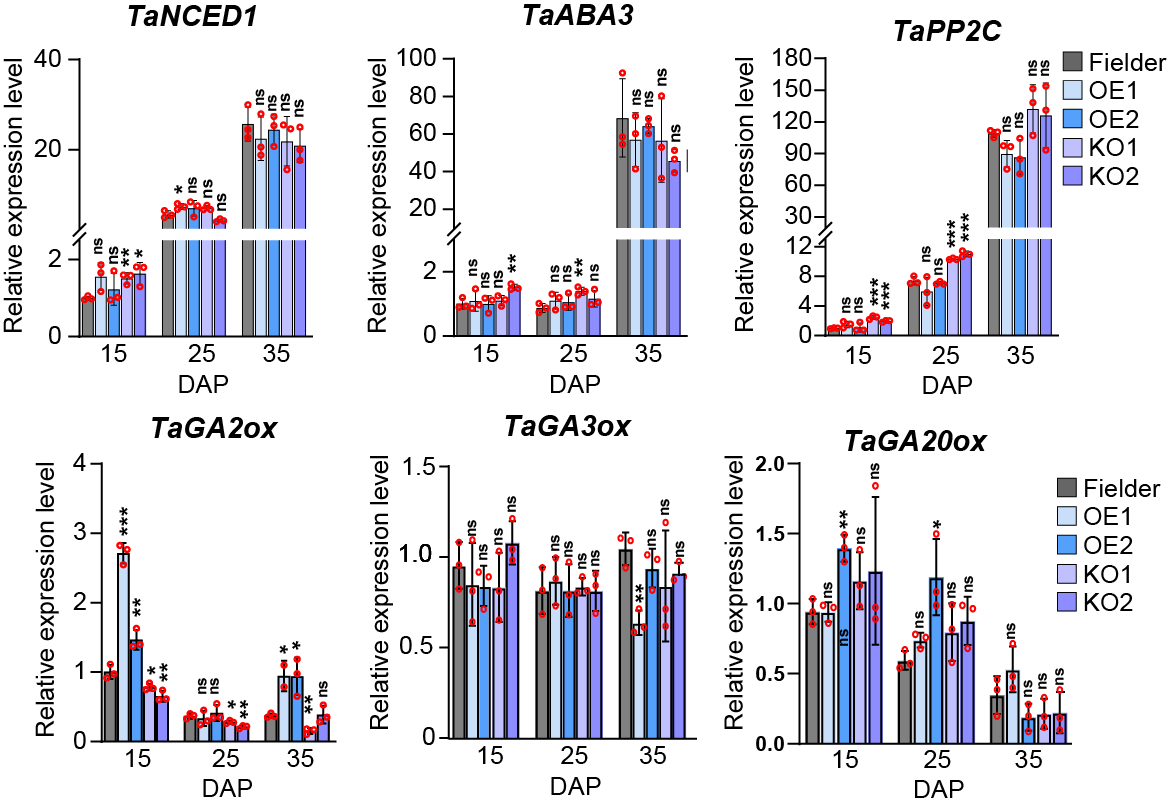


**Figure S11 Validation of expression changes of selected genes in Fielder and *TaMBY7* transgenic lines**

RT-qPCR were conducted using Fielder, *TaMYB7-A1* overexpression lines and *TaMYB7* knockout lines. *TaTubulin* was used as the internal control, and expression levels were normalized to Fielder at 15 DAP (set to 1). Data represent mean ± S.D. of three biological replicates. *, *P* < 0.05; **, *P* < 0.01; ***, *P* < 0.001; ns, *P* ≥ 0.05 (Student’s *t*-test).


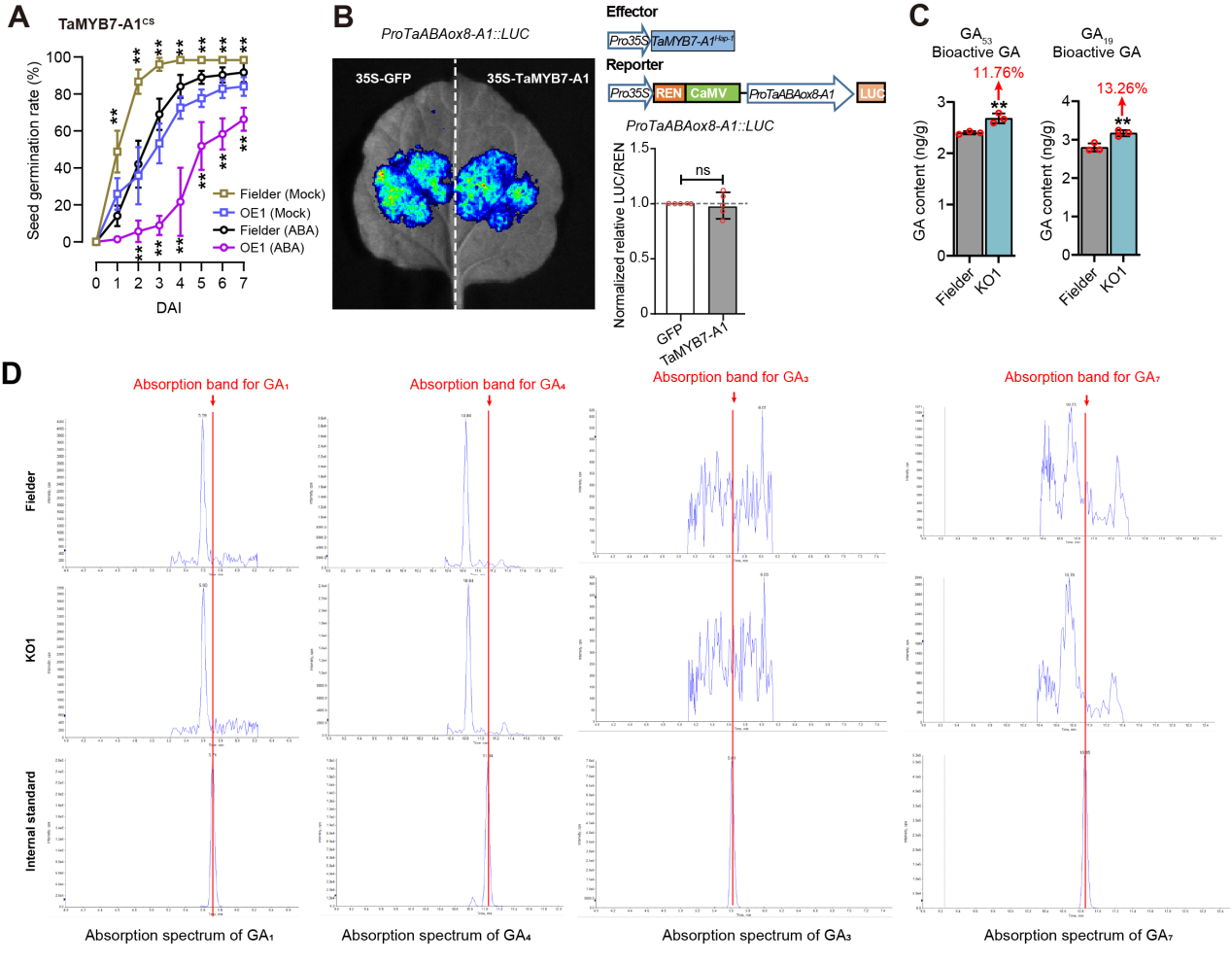


**Figure S12 TaMYB7-A1 affects ABA sensitivity and GA contents**

(A) Time-course of seed germination rate for Fielder and *TaMYB7-A1^CS^* OE line under 50 μM ABA treatment. Data represent mean ± S.D. of three biological replicates (50 seeds each). Color-coded asterisks indicate statistical significance between Fielder and OE1 within each treatment group (mock vs. mock; ABA vs. ABA) at each DAI (Student’s *t*-test). **, *P* < 0.01.

(B). Dual-luciferase reporter assays were performed in *N. benthamiana* leaves. The promoter regions of *TaABAox8-A1* were fused to firefly luciferase reporter and co-expressed with either TaMYB7-A1 (effector) or GFP (negative control). The relative LUC/REN ratios was shown. For each biological replicate, LUC/REN ratio were normalized to GFP control transfections (set to 1). Data are presented as mean ± S.D. of five independent transfections. ns, *P* ≥ 0.05 (Student’s *t*-test).

(C) GA_53_ and GA_19_ contents in 35 DAP grains from Fielder and KO1. Data represent mean ± S.D. of three biological replicates. **, *P* < 0.01 (Student’s *t*-test).

(D) Representative chromatograms of GA1, GA3, GA4 and GA7 analysis. Retention times are marked by vermilion vertical lines at the apex of standard peaks.


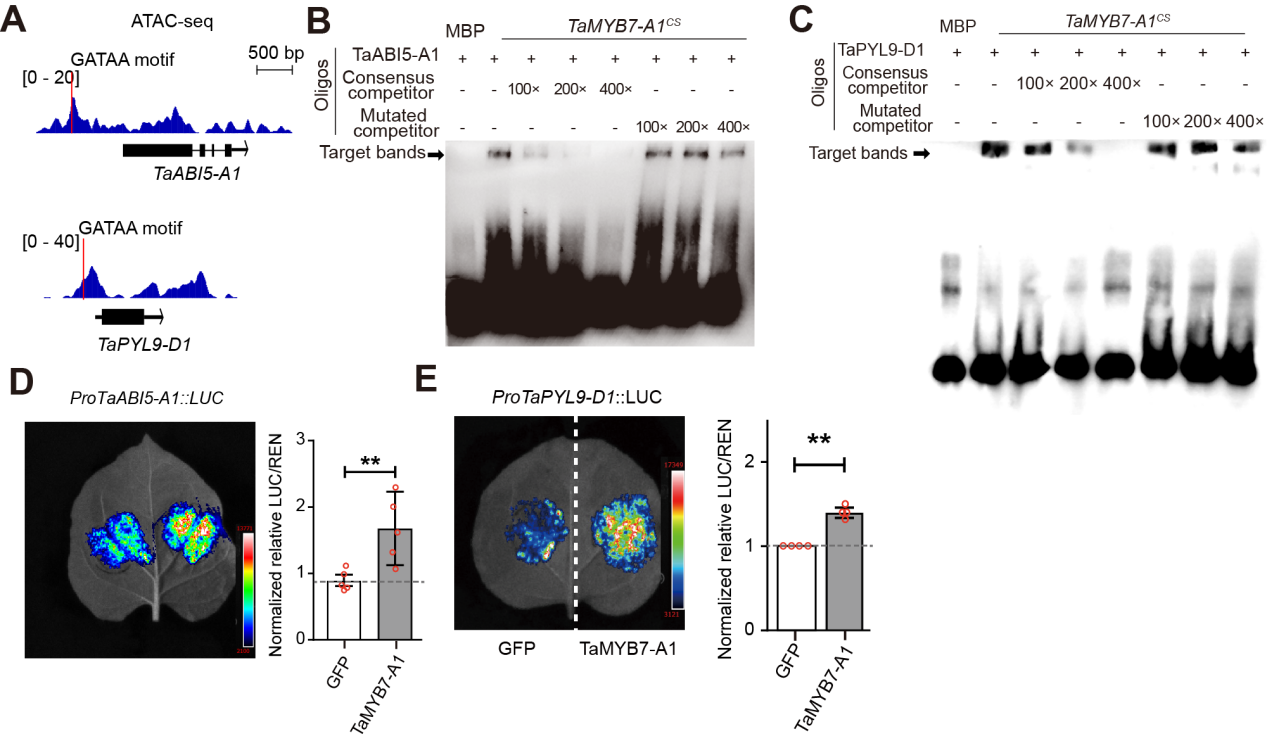


**Figure S13 TaMYB7-A1 activates the expression of *TaPYL9-D1***

(A) ATAC-seq profiling for *TaABI5-A1* and *TaPYL9-D1*. The ATAC-seq of 22 DAP embryo from Chinese Spring was used. The GATAA motif in their promoters is indicated by a red vertical line.

(B-C) EMSA confirming TaMYB7-A1 binding to the *TaABI5-A1* promoter (D) and *TaPYL9-D1* (E). ‘+’ and ‘-’ denote the presence/absence of probe or protein. The arrow marks protein-bound probe. Competitive binding used wild-type or mutant oligonucleotide at 100×, 200×, and 400× molar excess.

(D-E) Dual-luciferase reporter assay demonstrating TaMYB7-A1-mediated activation of the *TaABI5-A1* promoter (B) and *TaPYL9-D1* (C) promoter in *N. benthamiana* leaves. GFP served as negative control (dashed gray line). Data are the mean ± S.D. of four biological replicates, LUC/REN ratios were normalized to the GFP control set as 1. **, *P* < 0.01 (Student’s *t*-test).


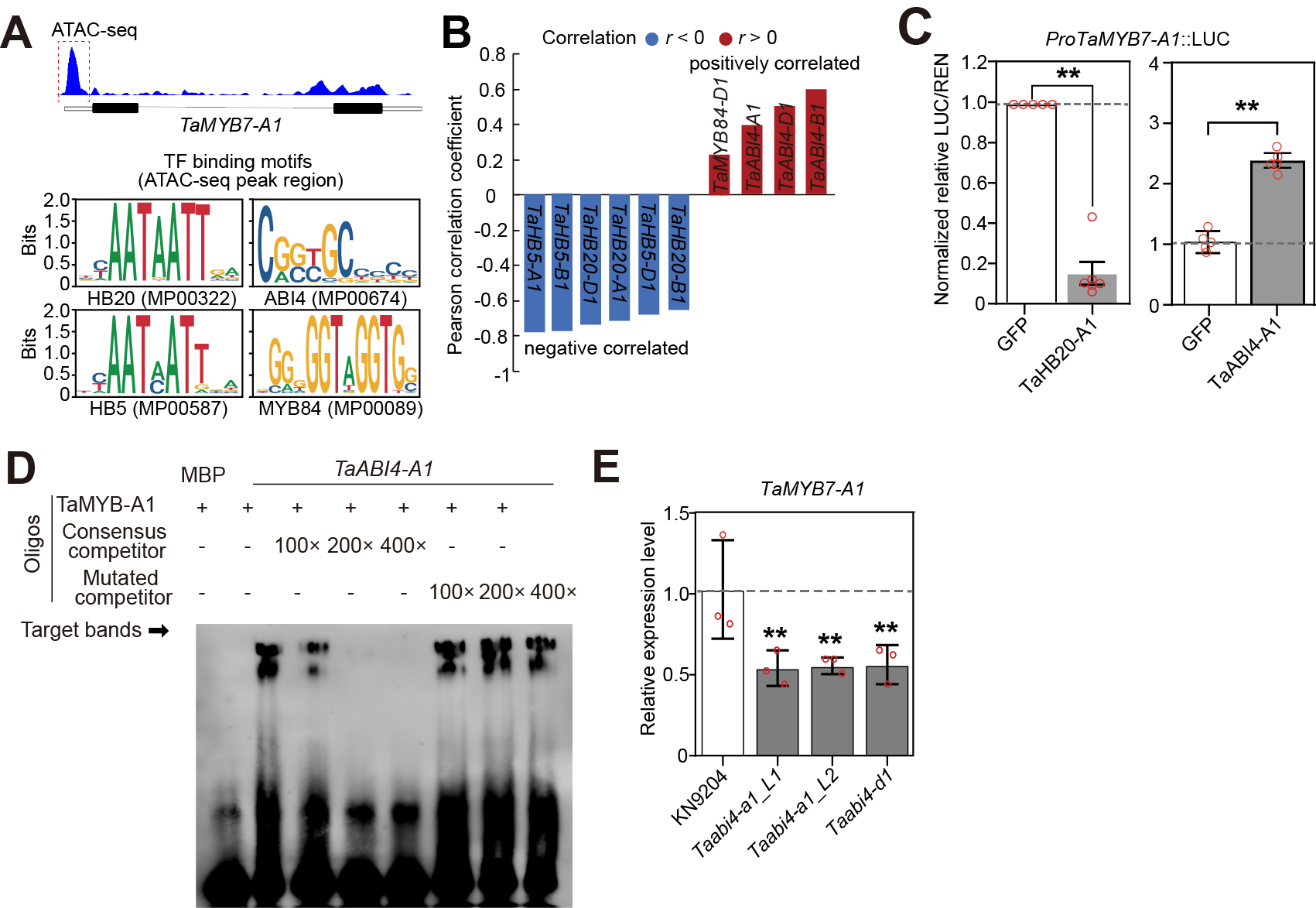


**Figure S14 TaABI4 activates the expression of *TaMYB7-A1***

1. Potential upstream regulators identified in the promoter accessible chromatin regions (pACRs, indicated by a red dashed box) of *TaMYB7-A1*. PlantTFdb was used to identify potential upstream regulators, and the identified motifs were showed bellow.

(B) Pearson correlation coefficient between the expression levels of the indicate genes and that of *TaMYB7-A1* at various stages of grain development.

(C) Dual-luciferase reporter assays in *N. benthamiana* leaves. The promoter regions of *TaMYB7-A1* were fused to firefly luciferase reporter and co-expressed with either TaHB20-A1, TaABI4-A1 (effector) or GFP (negative control). LUC/REN ratios were normalized to the GFP control transfections (set to 1). Data are presented as mean ± S.D. of five independent transfections. **, *P* < 0.01 (Student’s *t*-test).

(D) EMSA confirming TaABI4-A1 binding to the *TaMYB7-A1* promoter . ‘+’ and ‘-’ denote the presence/absence of probe or protein. The arrow marks protein-bound probe. Competitive binding used wild-type or mutant oligonucleotide at 100×, 200×, and 400× molar excess.

(E) RT-qPCR of *TaMYB7-A1* expression in 35 DAP wheat grains from wild-type KN9204 and *TaABI4* mutant lines. The analysis included two *TaABI4-A1* mutants and one *TaABI4-D1* mutant. *TaTubulin* was used as the internal control, and expression levels were normalized with KN9204 set to 1. Data are the mean ± S.D. of three biological replicates. **, *P* < 0.01 (Student’s *t*-test).


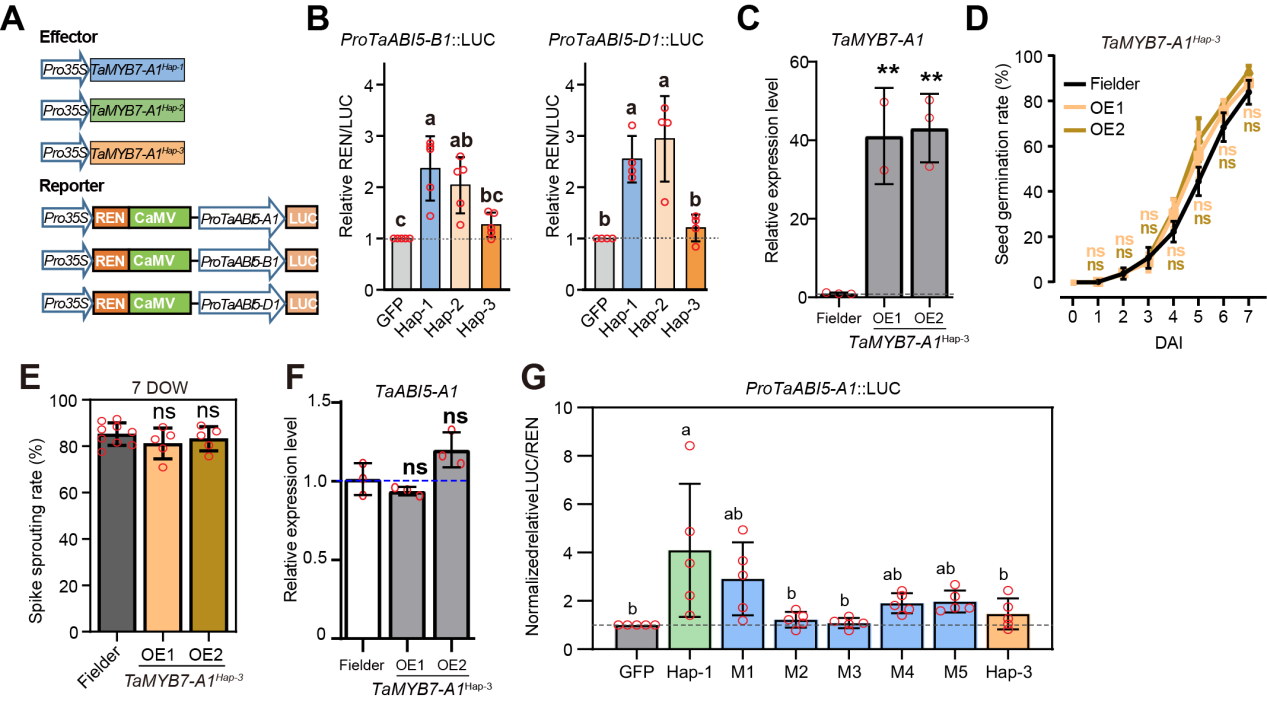


**Figure S15 *TaMYB7-A1^Hap-3^* fails to active downstream genes**

(A, B) Schematic diagram showing the vector design of effector and reporter for dual-luciferase reporter assays (b) of *TaABI5-A1* promoter activities in transiently transgenic *N. benthamiana* leaves by TaMYB7-A1 haplotypes (c). LUC/REN values were normalized with negative control GFP set to 1 (gray dashed line). Different letters indicating significant differences at *P* < 0.05 (Tukey’s HSD multiple comparisons test).

(C) RT-qPCR of *TaMYB7-A1* in Fielder and *TaMYB7-A1^Hap-3^* OE lines. *TaTubulin* was used as the internal control, and expression levels were normalized with Fielder set to 1. Data represent mean ± S.D. of three biological replicates. **, *P* < 0.01 (Student’s *t*-test).

(D) Seed germination rate for Fielder and *TaMYB7-A1^Hap-3^* OE lines. Data represent mean ± S.D. of three biological replicates (~50 grains each replicate). Student’s *t*-test were used to determine the statistical significance for differences at each time point. ns, *P* ≥ 0.05.

(E) Spike sprouting rate (SSR) for Fielder and *TaMYB7-A1^Hap-3^* OE lines. Data represent mean ± S.D. from 5-9 spikes. ns, *P* ≥ 0.05 (Student’s *t*-test).

(F) RT-qPCR of *TaABI5-A1* in Fielder and *TaMYB7-A1^Hap-3^* OE lines. *TaTubulin* was as the internal control, and expression levels were normalized to Fielder (set to 1). Data represent mean ± S.D. of three biological replicates. ns, *P* ≥ 0.05 (Student’s *t*-test).

(G) The bar graph comparing the activation activity of different TaMYB7-A1 haplotypes or mutated *TaMYB7-A1^Hap-1^* proteins on the expression of *TaABI5-A1* in *N. benthamiana* leaves. Data represent mean ± S.D. of five biological replicates. Different letters indicating a significant difference in means of LUC/REN at *P* < 0.05 (Tukey’s HSD multiple comparisons test).


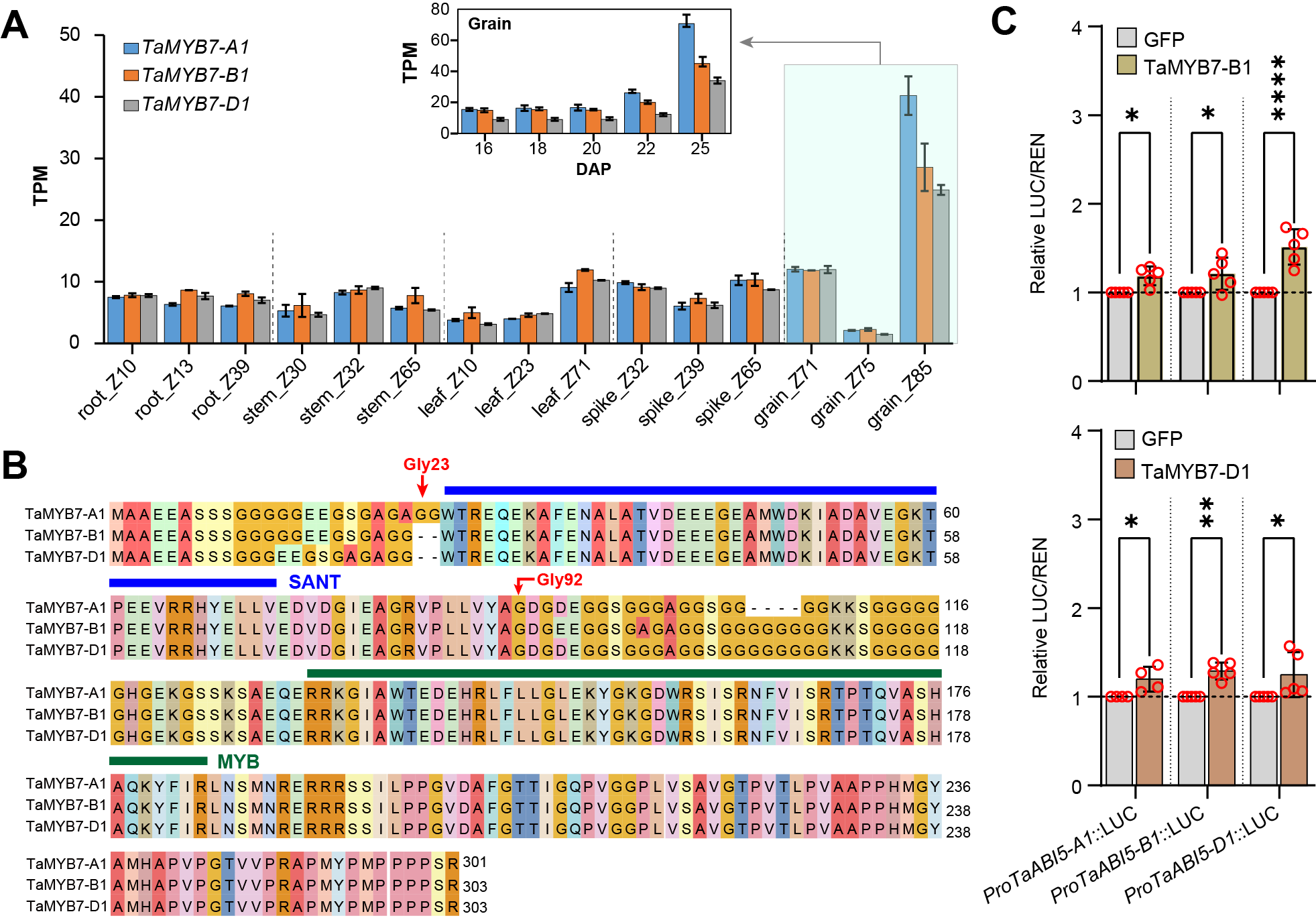


**Figure S16 Sequence and expression characterization of *TaMYB7-A1*, *TaMYB7-B1* and *TaMYB7-D1***

(A) Tissue-specific expression profiles of *TaMYB7-A1/B1/D1* (outer panel; obtained from Wheat Multi-Omics Database, http://202.194.139.32/expression/wheat.html), with seed-specific expression data (inner panel; our unpublished RNA-seq data of developing wheat seeds, see Supplemental Table 10 for the raw data of *TaMYB7-A1/B1/D1*). The seed developmental stages are indicated by days after pollination (DAP).

(B) Multiple sequence alignment of TaMYB7-A1, TaMYB7-B1, and TaMYB7-D1 protein variants. Blue and green bars above the sequences indicate conserved functional domains. Dashes represent amino acid gaps in the alignment. The conserved SANT, MYB domain and the critical amino acids, Gly23 and Gly92 is indicated.

(C) Dual-luciferase reporter assays in *N. benthamiana* leaves. The promoter regions of *TaABI5-A1/B1/D1* were fused to firefly luciferase reporter and co-expressed with either TaMYB7-B1, TaMYB7-D1 (effector) or GFP (negative control). LUC/REN ratios were normalized to the GFP control transfections (set to 1). *, *P* < 0.05; **, *P* < 0.01; ****, *P* < 0.0001 (Student’s *t*-test).


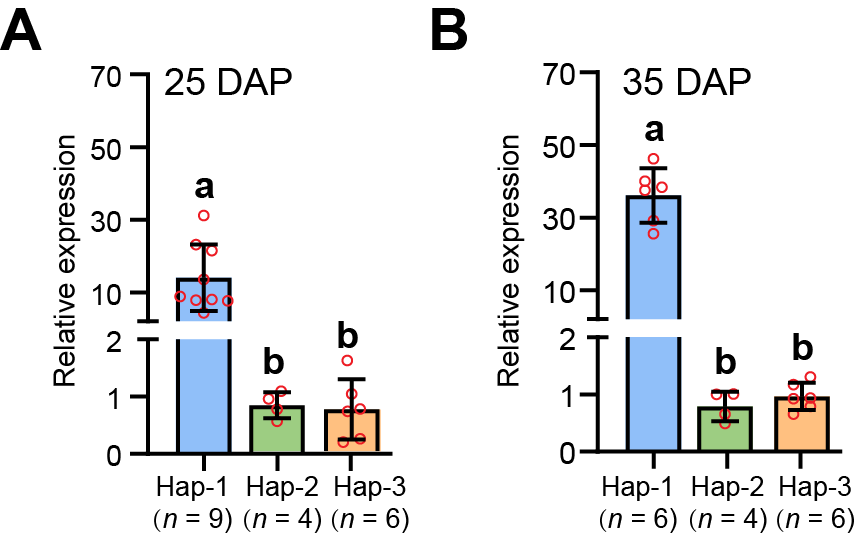


**Figure S17 Expression differences among *TaMYB7-A1* haplotypes in developing grains**

RT-qPCR of *TaMYB7-A1* expression among accessions with different *TaMYB7-A1* haplotypes in grains at 25 DAP (19 wheat accession) (A) and 35 DAP (16 wheat accession) (B). Data represent means ± S.D. of accessions, relative expression was normalized to SN21 (an accession of Hap-2). The means from three independent biological replicates of each accession were indicated by a dot. Different letters indicate statistical significance among haplotypes at *P* < 0.05 (Tukey’s HSD multiple comparisons test).


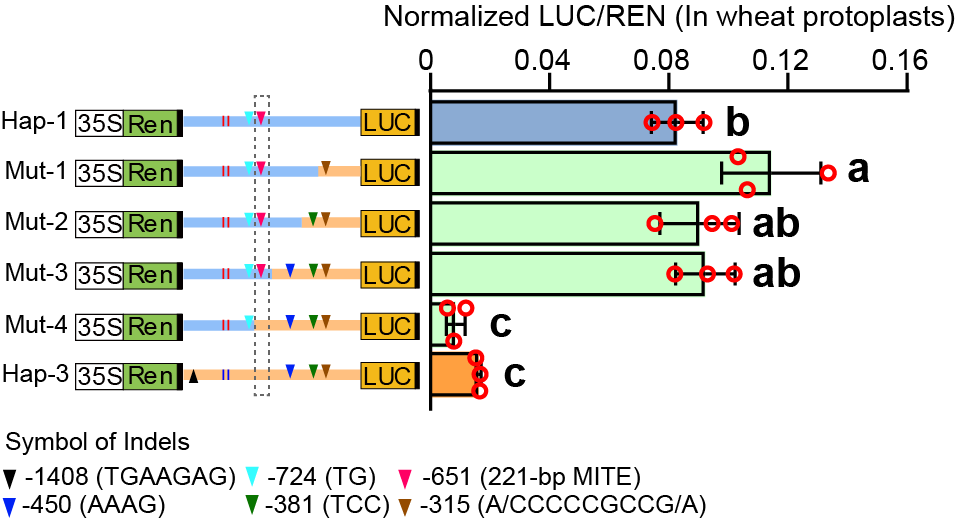


**Figure S18 A MITE insertion boosts T*aMYB7-A1^Hap-1^* expression**

Dual-luciferase reporter assays for *TaMYB7-A1* promoter activity in wheat protoplasts. Inverted triangles in different colors indicate InDels distributed in the *TaMYB7-A1* promoter region. Promoter fragments from Hap-1 and Hap-3 are indicated in the schematics in blue and orange boxes, respectively. The 221-bp MITE was the sole difference between Mut-3 and Mut-4, and this region was indicated by a dashed frame. Data represent mean ± S.D of three biological replicates. Different letters indicate significance among different promoter combinations at *P* < 0.05 (Tukey’s HSD multiple comparisons test).


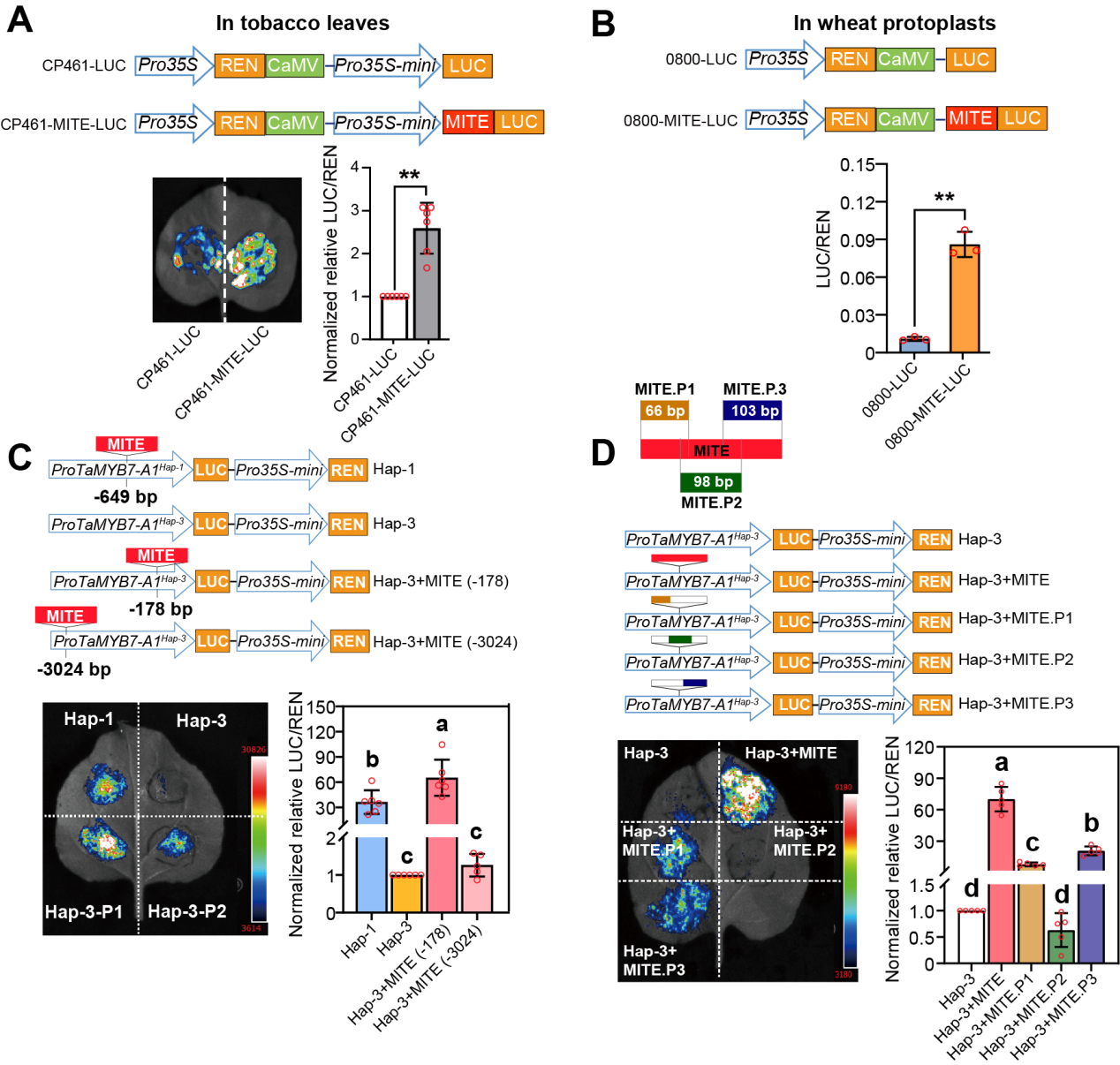


**Figure S19 A MITE transposon insertion in the *TaMYB7-A1^Hap-1^* promoter enhances its expression**

(A) Dual-luciferase reporter assays in *N. benthamiana* leaves to test the activation activity of MITE. The CP461-LUC, empty vector control; CP461-MITE, a MITE inserted upstream of LUC in CP461-LUC. Data are the mean ± S.D. of six biological replicates. LUC/REN ratios were normalized to empty vector control (set to 1). **, *P* < 0.01 (Student's *t*‐test).

(B) Dual-luciferase reporter assays in wheat protoplasts to test the activation activity of MITE. The 0800-LUC, empty vector control; 0800-MITE-LUC, a MITE inserted upstream of Luc in 0800-LUC. Data are the mean ± S.D. of three biological replicates. **, *P* < 0.01 (Student's *t*‐test).

(C) Dual-luciferase reporter assays in *N. benthamiana* leaves to test the effect of MITE insertion positions on LUC activity. Different letters indicate significance at *P* < 0.05 (Tukey’s HSD multiple-comparisons test).

(D) Dual-luciferase reporter assays in *N. benthamiana* leaves using series of MITE truncation for exploring the key segments for its enhancer activity. Different letters indicate significance at *P* < 0.05 (Tukey’s HSD multiple-comparisons test).


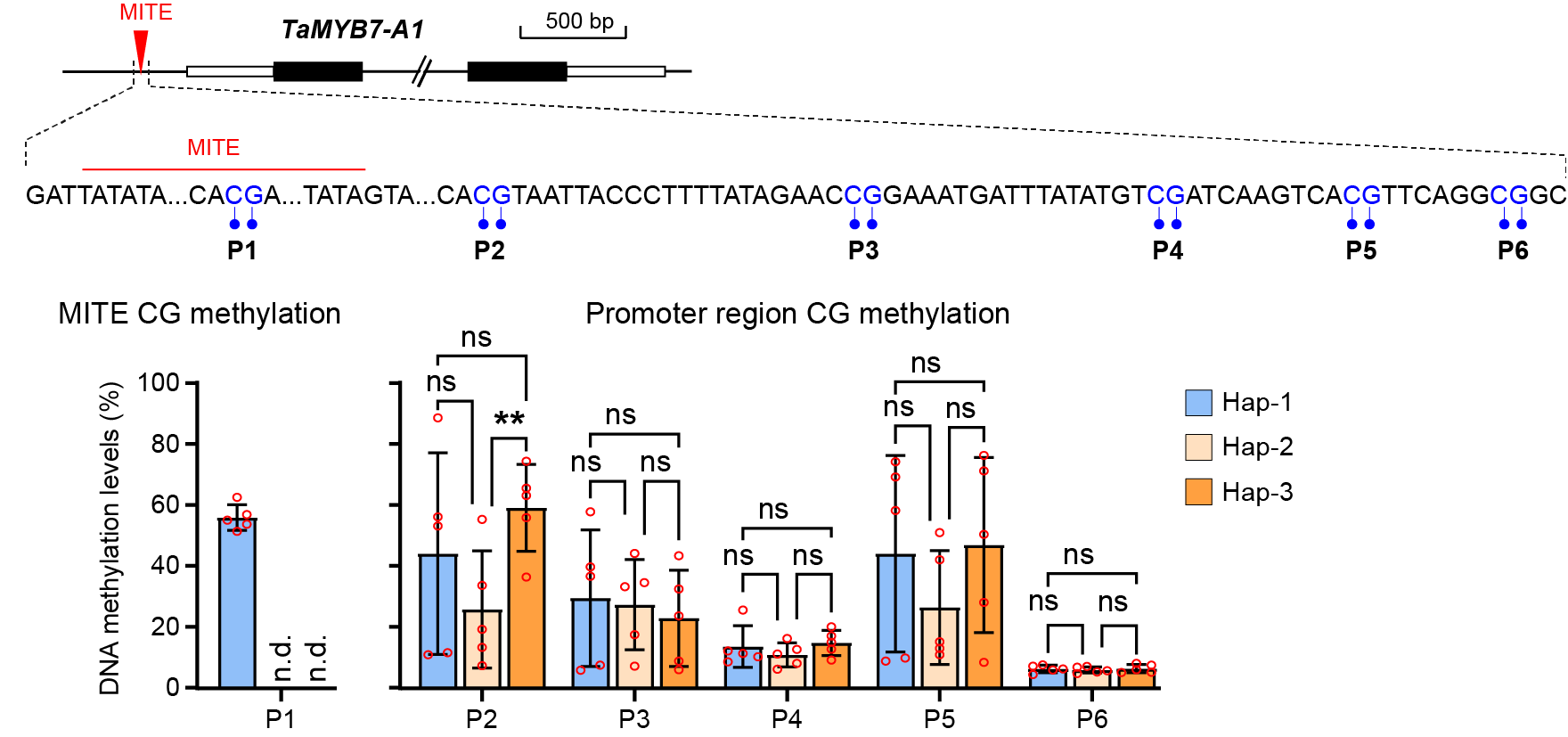


**Figure S20 Differential CG-methylation patterns in *TaMYB7-A1* haplotypes**

Bar graphs depict DNA methylation levels (%) determined by bisulfite sequencing (BS-seq) at six CG island loci (P1-P6) across *TaMYB7-A1* haplotypes (Hap-1, Hap-2, Hap-3). Locus P1 contains the MITE insertion specific to Hap-1. Error bars indicate S.D. of five wheat accessions. **, *P* < 0.01; ns, *P* ≥ 0.05 (Student's *t*‐test).

**Figure S21 TaAZF1 acts as a potential upstream regulator of *TaMYB7-A1***

(A) Sequence logos of over-represented motifs identified in MITE region by PlantTFdb.

(B) Pearson correlation coefficients between expression pattern of the indicated genes and *TaMYB7-A1*. The expression level of indicated genes and *TaMYB7-A1* across grain development stages in Chinese Spring were used for analysis.

(C) Expression dynamics of *TaMYB7-A1* and *TaAZF1* homologs genes across grain development in Chinese Spring. The transcript levels of each gene across stages were normalized to its maximum value (set as 100%) for cross-comparison.

(D) Dual-luciferase reporter assays in *N. benthamiana* leaves. Chemiluminescence from an *N. benthamiana* leaf transiently co-expressing *ProTaMYB7-A1::*LUC reporters, the TaAZF1-A1 test effector or negative control GFP.

(E) Yeast two‐hybrid assay of the physical interaction between TaAZF1 and TaABI4.


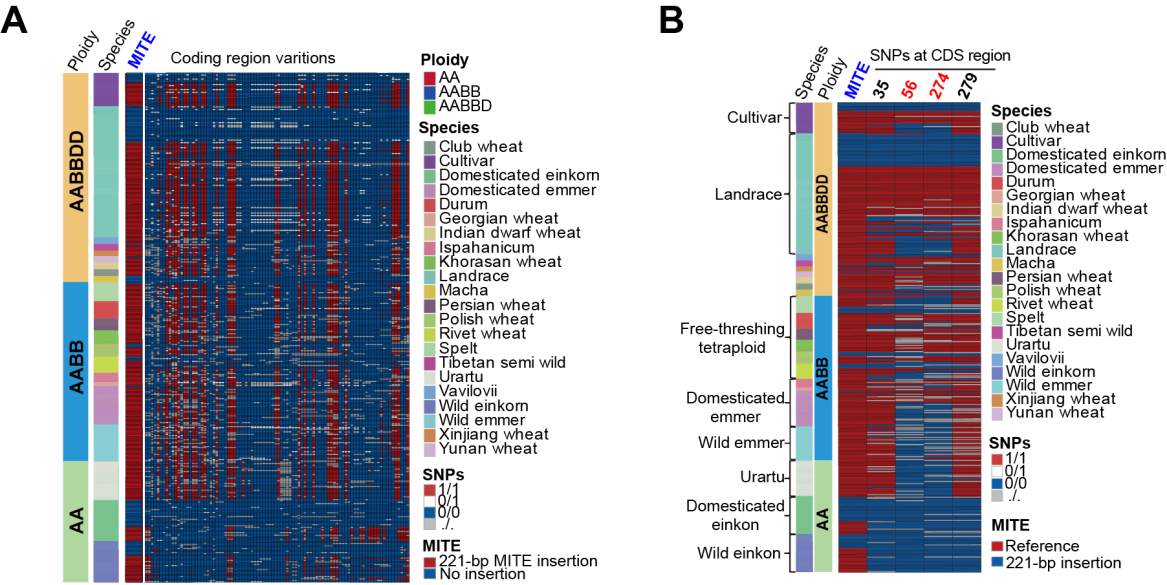


**Figure S22 *TaMYB7-A1^Hap-1^* originated from wild einkorn introgression**

Distribution of the MITE transposon, coding region variants (A) and key exon variants (B) in diploid, tetraploid and hexaploid wheat accessions. The MITE in promoter region is marked in blue and key SNPs causing amino acid substitutions/deletions are in red.


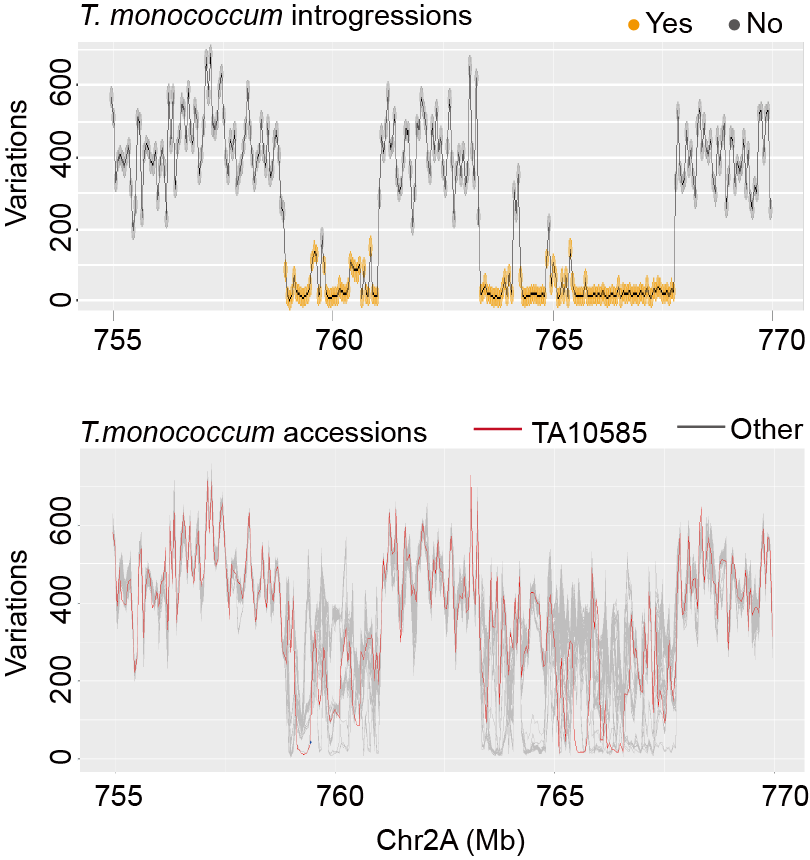


**Figure S23** Sequence-similarity analysis of the genomic introgression region (upper panel) and the most likely einkorn-wheat donor (lower panel).


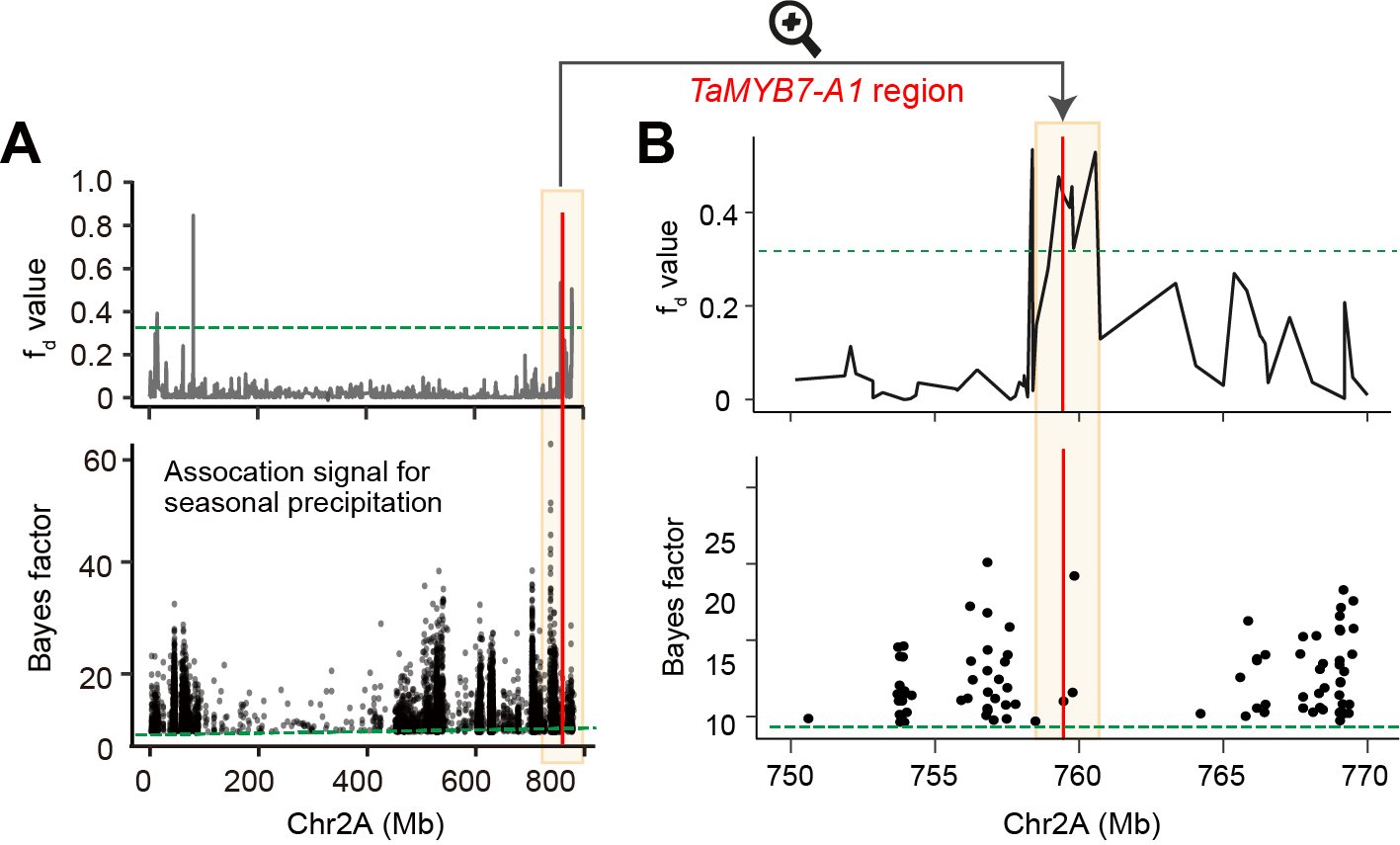


**Figure S24 Overlap between introgression and seasonal precipitation associated chromosome region**

Genomic profiling of wild einkorn-derived introgression signal (upper panel, wild einkorn to hexaploid) and seasonal precipitation association signals (bottom panel) on chromosome 2A (A) and *TaMYB7-A1* region (B). *TaMYB7-A1* is located in the overlapping genomic region of introgression signal and association signals with seasonal precipitation.


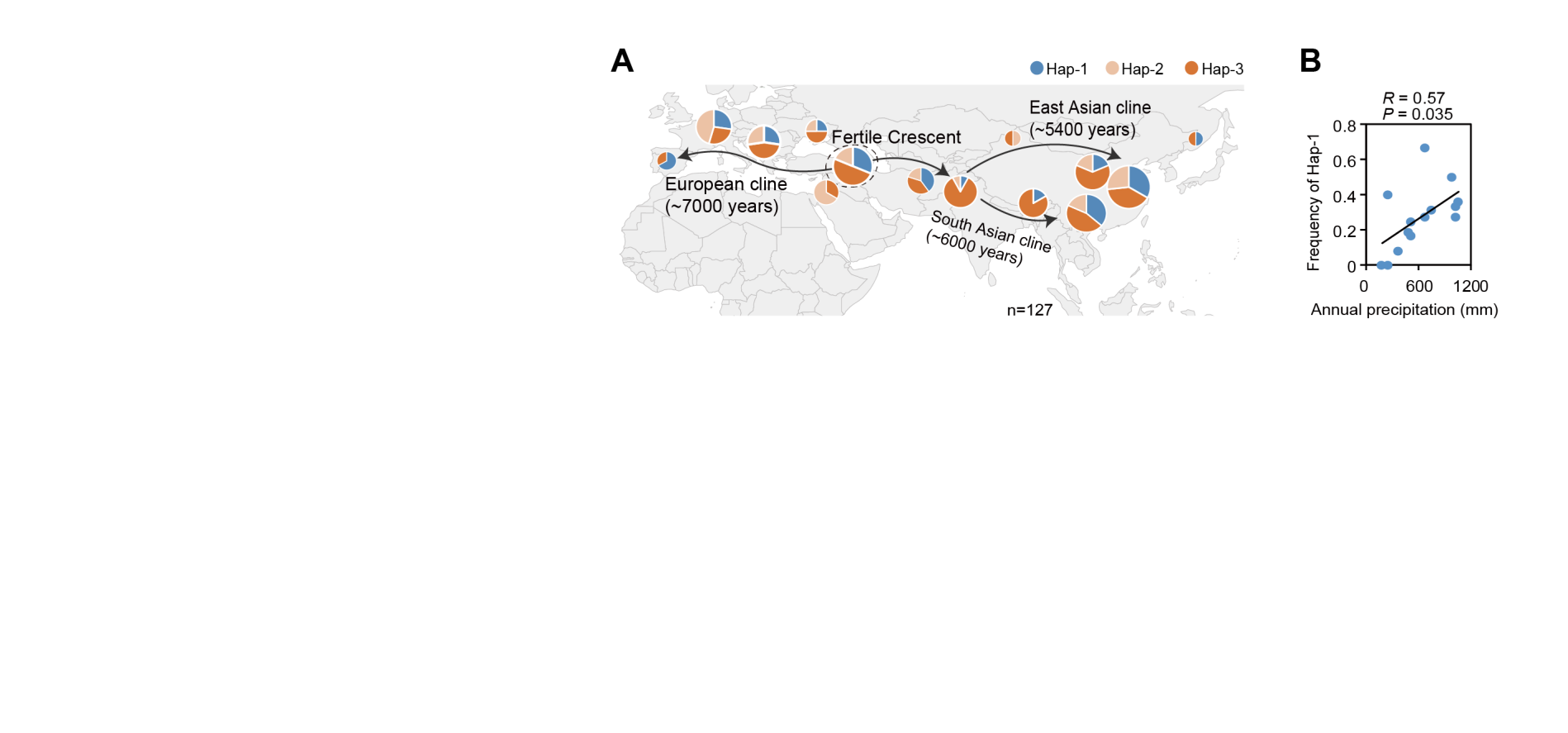


**Figure S25 *TaMYB7-A1* haplotype frequencies changes during the globally spreading of wheat**

Pie plots showing the change of *TaMYB7-A1* allele frequency during the propagation of landraces (A) and their correlation with annual precipitation at each location (B). Pearson correlation was used to calculated the regression trend.


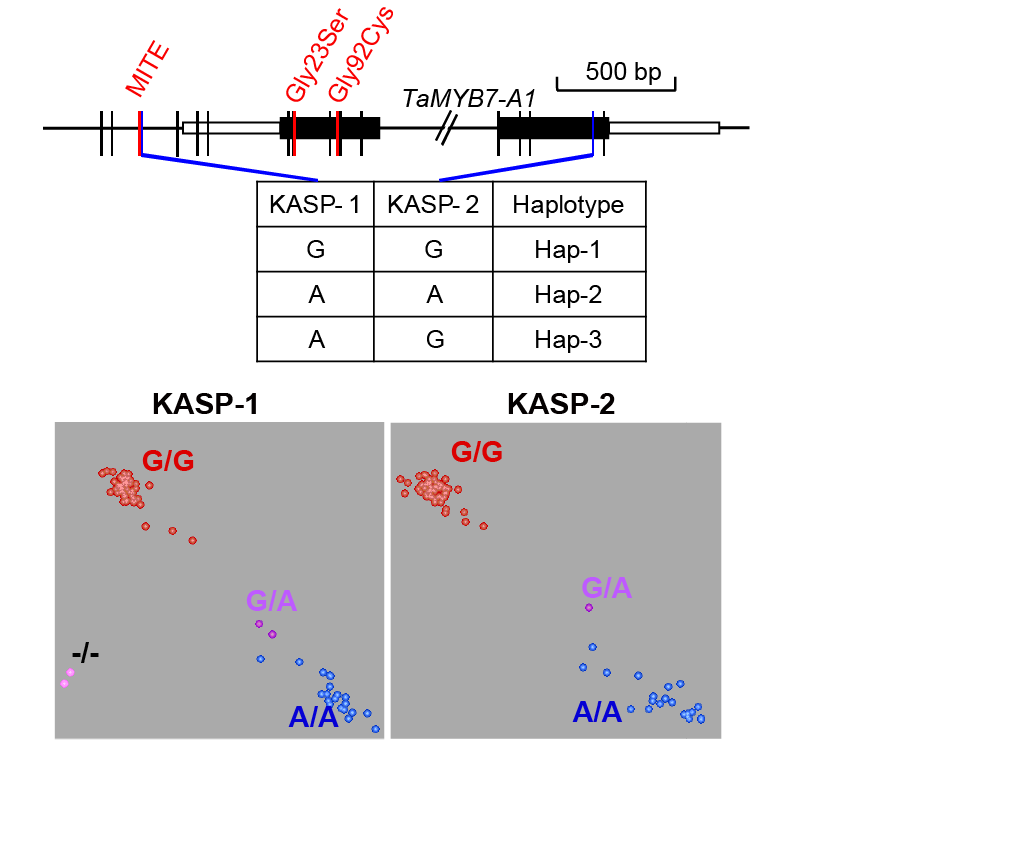


**Figure S26 Development of KASP markers for *TaMYB7-A1***

Gene structure diagrams illustrate the polymorphisms on *TaMYB7-A1*, and two KASP markers were developed to distinguish three *TaMYB7-A1* haplotypes.


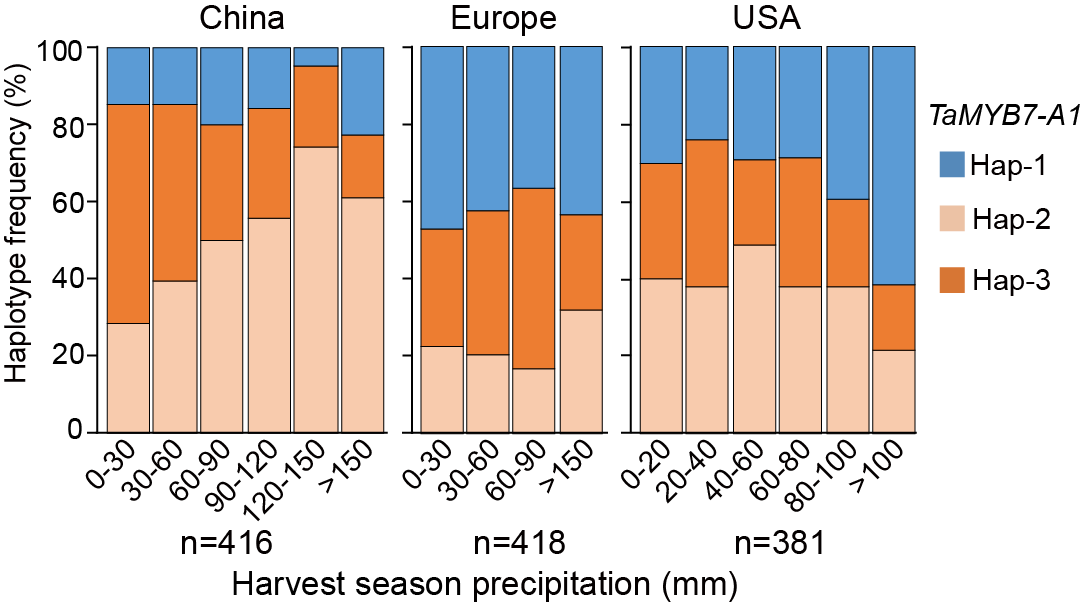


**Figure S27 Frequencies of *TaMYB7-A1* haplotypes in wheat varieties grouped by harvest-season precipitation**

*TaMYB7-A1* allele frequency in wheat varieties bred in locations with different seasonal precipitation in China, Europe and USA.


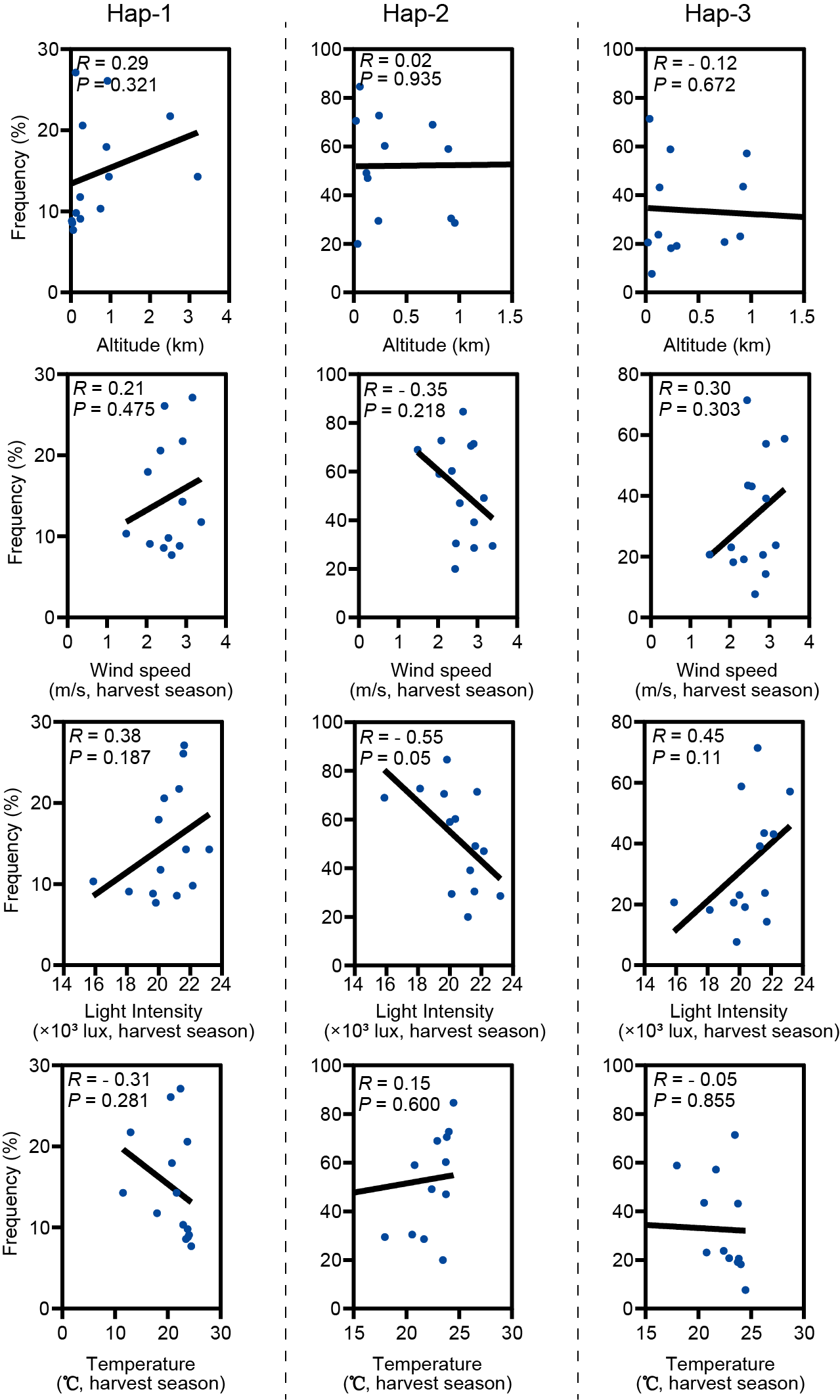


**Figure S28 Correlation between *TaMYB7-A1* alleles and environmental factors in wheat cultivars**

Scatter plots show the relationships between *TaMYB7-A1* alleles variation and four key environmental factors: altitude, wind speed, solar radiation, and growing-season temperature. Each data point represents an area; regression lines indicating significant correlations (*P* < 0.05, generalized linear models).


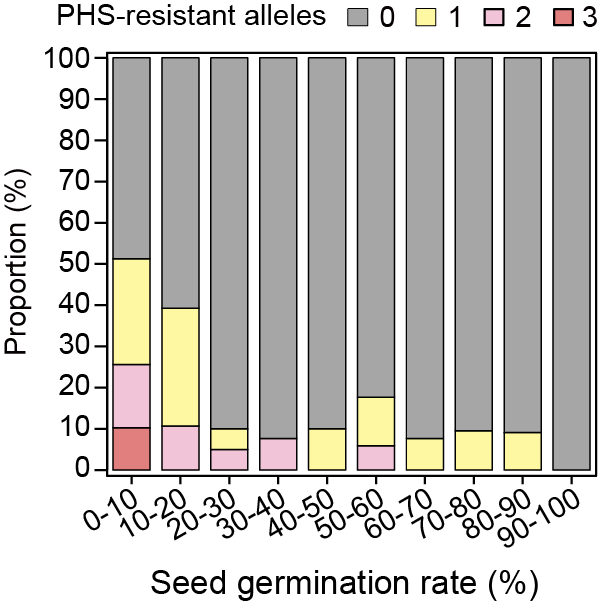


**Figure S29 Breeding selection and environmental correlation shape *TaMYB10-D* allele distribution in European wheat**

The proportion of accessions carrying different PHS-resistant alleles number of *TaMYB10-D1*, *TaAIRP2-A1*, and *TaMYB7-A1* across groups with varying seed germination rates.


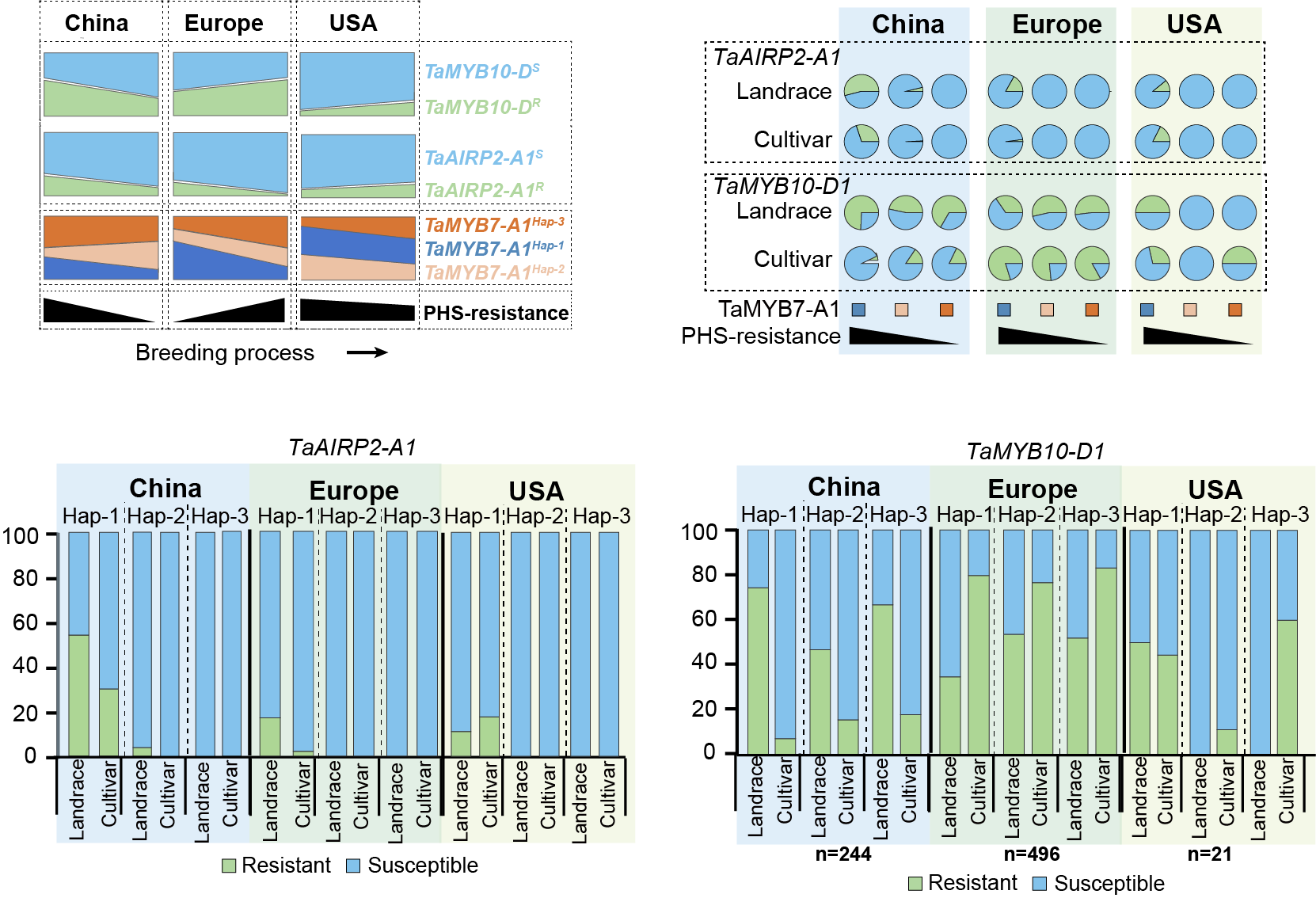


**Figure S30 Regional allele frequencies reveal linked selection between PHS-resistant and -susceptible haplotypes of *TaMYB7-A1* and *TaMYB10-D1*.**

Allele frequency of *TaMYB10-D1* for accessions carrying each *TaMYB7-A1* allele in China (n = 244), Europe (n = 496) and USA (n = 21). The PHS-resistant and PHS-susceptible allele for each gene is marked in green and blue, respectively.


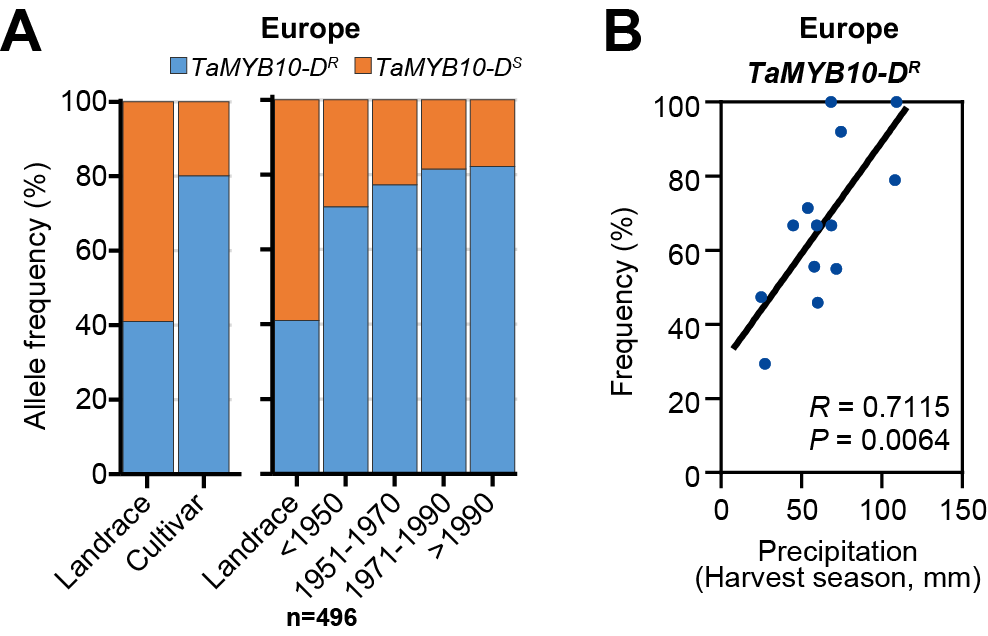


**Figure S31 Breeding selection and environmental correlation shape *TaMYB10-D* allele distribution in European wheat**

(A) Historical shifts in TaMYB10-D1 allele frequency during Europe wheat breeding. *TaMYB10-D^R^*, PHS-resistant allele; *TaMYB10-D^S^*, PHS-susceptible allele.

(B) Correlation of *TaMYB10-D^R^* allele with harvest-season precipitation across European wheat regions. Each dot represents a country; the trend lines represent the regression trend calculated by a general-linear model.


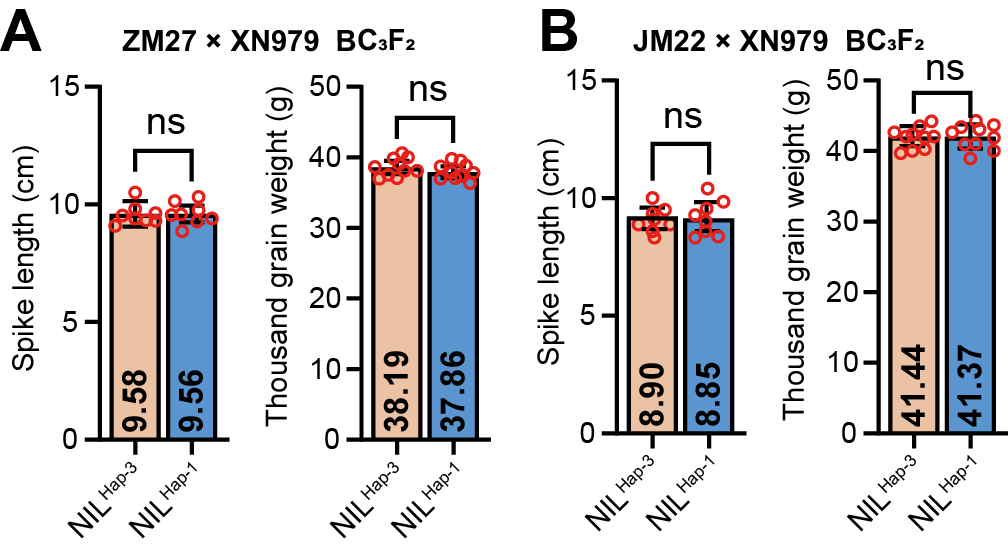


**Figure S32. TaMYB7-A1^Hap-1^ confers PHS-resistance without compromising agronomic traits**

(A-B) In the ZM27(a) and JM22(b) genetic backgrounds, the plant height and thousand grain weight (*n* ≥ 8) for NIL^Hap-3^ (in blue) and NIL^Hap-1^ (in orange). Data represent mean ± S.D. **, *P* < 0.01; ns, *P* ≥ 0.05 (Student’s *t*-test).
